# Safety of ibuprofen in infants younger than six months: A retrospective cohort study

**DOI:** 10.1371/journal.pone.0199493

**Published:** 2018-06-28

**Authors:** Paul Walsh, Stephen J. Rothenberg, Heejung Bang

**Affiliations:** 1 Pediatric Emergency Medicine, Sutter Medical Center Sacramento, Sacramento, CA, United States of America; 2 Instituto Nacional de Salud Pública, Centro de Investigación en Salud Poblacional, Cuernavaca, Morelos, Mexico; 3 Division of Biostatistics, Department of Public Health Sciences, University of California Davis, Davis, CA, United States of America; University of Manitoba, CANADA

## Abstract

**Objective:**

We hypothesized (1) that gastrointestinal (GI) and renal adverse events (AE) would occur more often in infants first prescribed ibuprofen before rather than after six months of age and (2) that ibuprofen would be associated with more adverse effects than acetaminophen in infants younger than six months.

**Methods:**

We created two partly overlapping retrospective cohorts of infants aged less than six months when California Medicaid first paid for ibuprofen or acetaminophen between 2004 and 2010. In the first cohort we compared the incidence rate ratio (RR) of GI and renal AE between those infants first prescribed ibuprofen before six months of age with those first prescribed ibuprofen after six months of age. In the second cohort we compared the RR of GI and renal AE between infants younger than six months prescribed ibuprofen (+/-acetaminophen) with those prescribed only acetaminophen.

**Results:**

We identified 41,669 prescriptions for ibuprofen and 176,991 prescriptions for acetaminophen in 180,333 eligible infants (median age 2.1 months). We did not observe higher RR of any AE in infants first prescribed ibuprofen before rather than after six months of age. Most infants prescribed ibuprofen were also prescribed acetaminophen. Any GI (adjusted (a)RR 1.25, 95% CI 1.13–1.38) and moderate or severe GI AE (aRR 1.24, 95% CI 1.09–1.40) were more common in infants younger than six months who were prescribed ibuprofen versus acetaminophen alone. Severe GI (aRR 0.63, 95% CI 0.27–1.45) and renal AE (aRR 1.84 95% CI 0.66–5.19) were not different between the ibuprofen (+/-acetaminophen) and acetaminophen-only groups.

**Conclusions:**

GI and renal AEs were not higher in infants younger than six months who were prescribed ibuprofen compared with those aged six to 12 months. AEs were increased in infants younger than six months who were prescribed ibuprofen compared with infants who were prescribed acetaminophen alone.

## Introduction

Ibuprofen, a nonsteroidal anti-inflammatory drug (NSAID), is an effective antipyretic and analgesic in children. Its mechanism of action is predominantly peripheral cyclooxygenase (COX) inhibition.[1] This distinguishes ibuprofen from acetaminophen which predominantly inhibits central COX-2 activity.[2, 3] Ibuprofen and acetaminophen are thus complementary and are often used concurrently in clinical practice.[4]

Ibuprofen use in the United States is widely confined to infants over six months of age because of safety concerns. The primary safety concerns about using ibuprofen in infants younger than six months of age are: adverse gastrointestinal (GI) effects,[5] risk of renal failure (RF),[6–8] increased risk of necrotizing fasciitis,[9, 10] and Reye’s Syndrome.[11] These concerns persist despite ibuprofen use in the neonatal intensive care unit[12, 13] and reassuring data and clinical experience between six and 24 months of age.[14] There is a dearth of information demonstrating harm from using ibuprofen in infants under six months of age and little commercial incentive to gather such data. A 1999 submission to the FDA by a pharmaceutical company requesting over-the-counter (OTC) labeling for infants less than six months of age was rejected as having too few infants (n = 319) in this age group 9S1 file Letter from FDA (S1 File).[15] Many US prescribers interpret lack of OTC labeling as meaning that ibuprofen is unsafe for infants younger than six months. In sharp contrast the British National Formulary provides specific dosing guidance for infants aged 1 to 3 months.[16]

We hypothesized that infants younger than six months of age prescribed ibuprofen (with or without concomitant acetaminophen use) would have a higher incidence rate of GI adverse events, renal failure or impairment, necrotizing fasciitis, and Reye’s syndrome than:

1. infants who were between six months and 12 months of age when first prescribed ibuprofen,
2. infants younger than six months who were prescribed acetaminophen alone, at 14-day and six-month follow-up.

## Methods

### Patients and data sources

The State of California Committee for Protection of Human Subjects (State of California IRB) and the California Department of Health Care Services (DHCS) Pharmacy Branch both approved this study. The requirement for individual patient consent was waived by the State of California Committee for Protection of Human Subjects. The data was de-identified by a DHCS analyst. Individual visit information and some protected health information variables including location within the state were required for this research. Because of the potential for re-identification of at least some individuals by combining the data with other publicly available sources the State of California Committee for Protection of Human Subjects has mandated destruction of the dataset following completion of the research. These data can be obtained by direct application to both DHCS and State of California Committee for Protection of Human Subjects subject to both agencies’ approval. These are third party data (i.e. not gathered or owned by the authors). Others would be able to access these data in the same manner as we did. The authors did not have any special access privileges that others would not.

Data was obtained from the Medicaid Management Information System/Decision Support System (MMIS/DSS). This database contains details of paid pharmacy and medical provider claims. The database includes paid prescriptions, and up to two diagnosis codes per paid medical provider encounter.

We used this database to create a cohort of infants for whom DHCS had paid for at least one prescription for either acetaminophen or ibuprofen before six months of age between 2004 and 2010. We followed the cohort for six months up to 12 months of age following first ibuprofen exposure. If we had no evidence of visits six months following the visit when the drug was prescribed, we censored the case at the last known visit.

Diagnoses were based *on International Classification of Disease Clinical Modification* 9 (ICD-9-CM) codes in encounter diagnosis fields. ICD-9-CM and drug coding in California Medicaid is generally of high quality,[17] and a similar methodology to ours has been used in pediatric drug safety studies of stimulants.[18, 19]

### Study outcomes

#### Gastrointestinal outcomes

We categorized medical visits for gastrointestinal (GI) symptoms after the prescription of ibuprofen as mild, moderate, and severe. GI adverse events were classified as mild if confined to vomiting without diarrhea; moderate when abdominal pain, gastritis, duodenitis, and/or ulcer without bleeding was diagnosed; and severe if bleeding or obstruction occurred. If more than one potential GI adverse event was diagnosed during a visit, we assigned the more severe categorization. The ICD-9-CM codes used to identify GI adverse events are shown in Table 1.

**Table 1:** ICD-9-CM codes for severity of gastrointestinal adverse events.

Diagnosis of gastrointestinal adverse events	ICD-9-CM codes
Mild	787.1, 789, 789.66, 789.65, 787.0, 787.01, 787.03, 536.2, 787.9
Moderate	533, 533.3, 533.30, 533.31, 534, 534.9, 534.90, 534.91, 530.2, 530.20, 535, 535.0, 535.00, 535.4, 535.40, 535.5, 535.50, 535.6, 535.60, 532, 532.3, 532.30, 532.9, 532.90, 538, 534, 534.9, 534.90, 533.9
Severe	533.0, 533.00, 533.01, 533.10, 533.11, 533.2, 533.2, 533.20, 533.21, 533.31, 533.91, 789.4, 790.01, 535.01, 535.21, 535.51, 535.61, 569.83, 533.1, 530.4, 531.20, 531.21, 531.2, 531.1, 531.10, 531.11 532.20, 532.21, 532.2, 532.1, 531.20, 532.91, 532.0, 532.00, 532.11, 530.21, 530.4, 530.82, 534.91, 533.9, 578

#### Renal outcomes

The degree of renal failure or impairment is not specified in the MMIS/DSS database although acute as distinct from chronic or unspecified onset can be coded. Renal impairment and failure are a matter of degree of elevation in the serum creatinine, but the actual values were not stored in the MMIS/DSS database. Both renal failure and impairment were rarely diagnosed in infants, so we combined them. Chronic renal failure was identified using ICD-9-CM codes 585.x, acute renal failure 584.(x). When ICD-9-CM code 586 (renal failure duration not specified) was encountered, it was treated as acute if there was no previous diagnosis of renal failure.

#### Necrotizing fasciitis and Reye’s syndrome

We diagnosed necrotizing fasciitis based on the ICD-9-CM code 728.86 and Reye’s syndrome using the code 331.81.

### Statistical analyses

We used mean and standard deviation (SD) to describe reasonably normally/symmetrically distributed data and median and interquartile range (IQR) to describe severely non-normally/skewed distributed data. Outcomes were expressed as incidence per 10,000 infant days exposure, and rate and odds ratios with the 95% confidence interval (CI). Data management and statistical analysis were performed using Stata 14.1 (Statacorp LLP, College Station, TX).

#### Hypothesis 1

To test hypothesis 1, we compared the rate of adverse events between infants first prescribed ibuprofen before and after six months of age using survival analysis over 14 days and six months. Patients became at risk when the first prescription for ibuprofen was filled. The infant was defined as a new user when this first prescription for ibuprofen was filled. We compared the incidence rates of events in those who were prescribed ibuprofen (with or without acetaminophen) to those who were prescribed their first dose of ibuprofen when six months of age or older. We excluded those who were prescribed ibuprofen both before six months of age and between six and 12 months of age. We estimated the crude rate ratio (RR) of each outcome. We also estimated an adjusted rate ratio (aRR) using Mantel-Haenszel stratification to control for a prior history of the event being sought. We plotted Kaplan-Meier curves for 14-day follow-up to reflect short term adverse effects as well as for 6 months follow-up to account for possible subsequent use of left-over ibuprofen from a previous illness. We performed the Wilcoxon test for equality of survivor functions’ test because we anticipated that adverse events caused by the drug would tend to occur earlier rather than later.

#### Hypothesis 2

To test hypothesis 2, we used two methods to compare the effect of ibuprofen (with or without acetaminophen) and acetaminophen alone on the incidence rates of subsequent adverse events.

In the first method, survival analysis for recurrent events patients became at risk from the time the first prescription for an antipyretic was filled but, if first prescribed acetaminophen and subsequently prescribed ibuprofen, changed from the non-ibuprofen group to the ibuprofen group when the first prescription for ibuprofen was filled. We did this because co-prescription was very common. We estimated an adjusted RR for each outcome using Mantel-Haenszel stratification to control for prior medical history of the event being sought and age.[20, 21]

In the second method we performed fixed effects panel logistic regression to minimize time-invariant omitted variable bias by allowing subjects to serve as their own controls. We estimated these regressions at 14-day and six-month follow up for the whole sample, and at six-month follow up for infants prescribed only acetaminophen or ibuprofen but not both. Because of the small number of infants getting ibuprofen but not acetaminophen we were unable to provide reliable estimates of the effect of ibuprofen alone on renal function. We adjusted for prior medical history of the events being sought as independent variables[22] but avoided adjusting for time-varying variables.[23]

We performed separate analyses for 14-day and six-month follow up (up to one year of age) to account for short and longer term adverse events (e.g. possible subsequent use of left-over ibuprofen from a previous illness).

#### Variables used for adjustment

For both hypotheses if an outcome of interest occurred prior to the first prescription for ibuprofen then this event was classified as a prior medical history of the outcome and included as an independent variable in the adjusted analysis. Accordingly, we adjusted the analysis of renal outcomes using prior history of renal impairment. We adjusted the analysis of ‘severe’ and ‘moderate or severe’ gastrointestinal outcomes using prior history of ‘moderate or severe’ gastrointestinal diagnoses. For ‘mild, moderate, or severe’ gastrointestinal outcomes we adjusted for a prior history of any ‘mild, moderate, or severe’ gastrointestinal diagnoses.

We adjusted for age measured in months in addition to the dichotomous six-month age threshold imposed by study design. We did this because of the potential for differences in outcomes even within a six-month age range. The six-month threshold was chosen because this is the age limit where many prescribers avoid using ibuprofen citing the lack of OTC labeling for this age group.

## Results

We identified 180,333 infants for whom a caregiver filled a prescription for an antipyretic within the first six months of life. We had subsequent visit information for 178,336 (99%). Their median age at baseline was 2.1 months (IQR, 1.8, 3.0) and 90,032 (50.0%) were male. We identified 41,669 filled prescriptions for ibuprofen and 176,991 for acetaminophen in infants younger than six months. We identified 74,274 subsequent prescriptions for ibuprofen and 501,261 subsequent prescriptions for acetaminophen between six and 12 months of age. There were only 1,724 infants who were prescribed ibuprofen but never acetaminophen during the study period. The sample demographics are described in Table 2.

**Table 2:** Demographic description of the sample. SD; standard deviation, IQR; interquartile range.

N = 180,333	Ibuprofen	Acetaminophen	Full Sample
Number filling first prescription	41,669	176,991	180,333
Mean age months (SD)	3.8 (1.6)	2.5 (1.3)	2.5 (1.3)
Median age months (IQR)	4.2 (2.5–5.2)	2.1 (1.9–3.2)	2.1 (1.89–3.0)
0 Prescriptions	138,664 (77%)	1,720 (1%)	0 (0%)
1–3 Prescriptions	39,332 (22%)	138,336 (77%)	25,542 (84%)
>3 Prescriptions	2,337 (1%)	40,277 (22%)	4,994 (16%)

The adverse events observed in the entire sample are reported in **Table 3**. Not all these events were included in the analysis because of the cohort definitions needed to test our hypotheses. Renal events were rare; there were 172 episodes of renal impairment or failure in 49 infants (0.03%) of these, and 90 in 37 (0.02%) infants were coded as acute. There were five cases of necrotizing fasciitis in four patients; none had previously filled a prescription for ibuprofen. There were no cases of Reye’s syndrome. Consequently, we dropped Reye’s syndrome and necrotizing fasciitis from the rest of our analysis.

**Table 3:** Number of adverse events in the total sample. Total N 180,333.

Outcome	Number of events
GI adverse events—Mild, moderate, or severe	7,692
GI adverse events—Moderate or severe	4,439
GI adverse events—Severe	738
Any renal adverse events	172
Acute renal impairment/failure	90

### Hypothesis 1

We included 31,561 infants in this cohort. The mean age of those younger than 6 months was 4.5 months (SD 1.1), in those 6 months or older the mean was 8.8 months (SD 1.7) and 52.8% were male.

#### GI events

**Fig 1:**
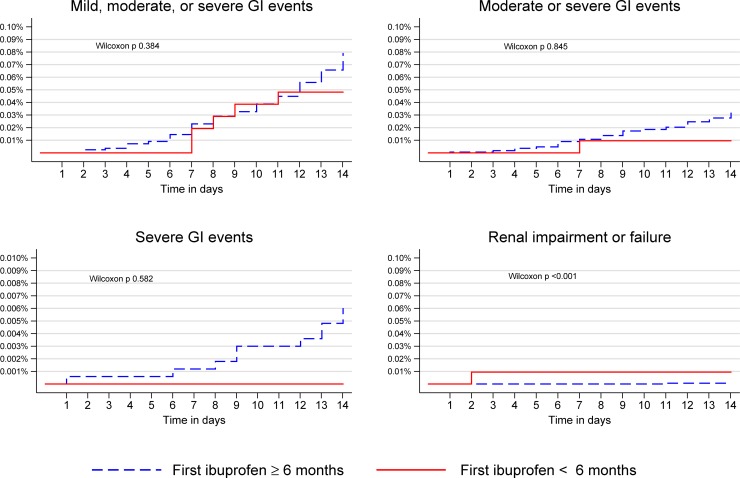
Kaplan-Meier failure curves for adverse events during 14-day follow-up after the first prescription of ibuprofen. GI; Gastrointestinal.

**Fig 2:**
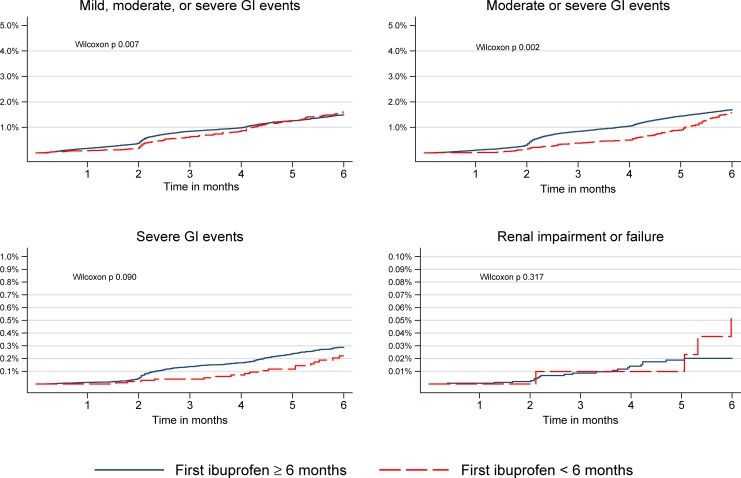
Kaplan-Meier failure curves for adverse events during six-month follow-up after the first prescription of ibuprofen. GI; Gastrointestinal.

We observed 1,069 adverse GI events among infants receiving ibuprofen, of these 637 were moderate, and 85 were severe. The timing and proportion of adverse effects are shown graphically in Figs 1 & 2. In adjusted analysis we found a lower incidence rate of any GI adverse events (aRR 0.58, 95% CI 0.50–0.66) and a similar incidence rate of severe GI events (aRR 0.72, 95% CI 0.47–1.09) when ibuprofen was first prescribed before (rather than after) six months of age; see Tables 4 and 5. We therefore surmise that adverse GI outcomes were not more frequent in infants who are prescribed ibuprofen before rather than after six months of age.

**Table 4:** Hypothesis 1 at 14-day follow up.

Outcome		Overall	Ibuprofen < 6 months		Ibuprofen ≥6 months		Crude RR	RR adjusted for age and past medical history
Sample N 31,561	N events	Incidence per 10,000 person-days	n events	Incidence per 10,000 person-days	n events	Incidence per 10,000 person-days		
**GI adverse events**								
Mild, moderate, or severe	535	10.90 (10.01 11.86)	188	10.83 (9.40 12.50)	347	10.94 (9.84 12.15)	0.99 (0.83 1.18)	1.00 (0.84 1.20)
Moderate or severe	224	4.56 (4.00 5.20)	71	4.09 (3.24 5.16)	153	4.82 (4.12 5.65)	0.85 (0.64 1.12)	0.91 (0.68 1.20)
Severe	42	0.85 (0.63 1.16)	13	0.75 (0.43 1.29)	29	0.91 (0.64 1.32)	0.82 (0.43 1.58)	0.83 (0.44 1.59)
**Renal adverse events**								
Any impairment or failure	22	0.45 (0.30 0.68)	15	0.86 (0.52 1.43)	7	0.22 (0.11 0.46)	3.92 (1.60 9.61)	1.32 (0.50 3.52)
Acute impairment or failure	10	0.20 (0.11 0.38)	7	0.40 (0.19 0.85)	3	0.09 (0.03 0.29)	4.27 (1.10 16.50)	2.38 (0.40 14.10)

Unadjusted and Mantel-Haenszel adjusted estimates of the rate ratio of an adverse event depending on whether ibuprofen was first prescribed before or after six months of age. 95% confidence intervals are in parentheses. Includes infants who were also prescribed acetaminophen. CI; confidence interval, RR; rate ratio.

**Table 5:** Hypothesis 1 at six-month follow up.

Outcome	Overall		Ibuprofen < 6 months		Ibuprofen ≥6 months		Crude RR	RR adjusted for age and past medical history
Sample N 31,561	N events	Incidence per 10,000 person-days	n events	Incidence per 10,000 person-days	n events	Incidence per 10,000 person-days		
**GI adverse events**								
Mild, moderate, or severe	1,069	2.88 (2.70 3.06)	434	2.39 (2.1 2.62)	635	3.36 (3.11 3.63)	0.71 (0.30 0.80)	0.72 (0.63 0.81)
Moderate or severe	637	1.72 (1.59 1.86)	277	1.52 (1.35 1.71)	360	1.9 (1.72 2.11)	0.8 (0.68 0.94)	0.84(0.72 0.98)
Severe	85	0.23 (0.19 0.28)	35	0.19 (0.14 0.27)	50	0.26(0.20 0.35)	0.72(0.47 1.12)	0.75(0.49 1.15)
**Renal adverse events**								
Any impairment or failure	36	0.1 (0.07 0.14)	24	0.13 (0.09 0.20)	12	0.06 (0.04 0.1)	2.08(1.04 4.16)	0.62 (0.28 1.37)
Acute impairment or failure	18	0.05 (0.03 0.08)	11	0.06 (0.03 0.11)	7	0.04 (0.02 0.08)	1.63(0.63 4.21)	0.81(0.24 2.67)

Unadjusted and Mantel-Haenszel adjusted estimates of the rate ratio of an adverse event depending on whether ibuprofen was first prescribed before or after six months of age. 95% confidence intervals are in parentheses. Includes infants who were also prescribed acetaminophen. CI; confidence interval, RR; rate ratio

#### Renal events

Renal impairment or failure occurred in 36 infants treated with ibuprofen. The timing and proportion of adverse effects are shown graphically in Figs 1 & 2. Ibuprofen use before six months of age was associated with increased renal adverse events in unadjusted analysis (RR 2.08, 95% CI 1.04–4.16). When adjusted for prior history of renal failure/impairment the direction of effect was reversed (aRR 0.42, 95% CI 0.17–1.08). This change in coefficient sign would be consistent with prescribers avoiding ibuprofen in infants with known renal failure (Table 5). We found no evidence that the age of ibuprofen administration is associated with adverse renal outcomes.

### Hypothesis 2

We had 167,523 infants with more than one visit in this cohort. Their mean age at cohort inception was 2.4 months (SD 1.3), and 49.8% were male.

#### GI events

We observed 5,773 GI adverse events among infants who received acetaminophen and/ or ibuprofen prior to six months of age; 3,397 of these were moderate or severe, and 570 were severe. There was little difference in most outcomes at 14-day follow up (Table 6). At six-month follow up there were higher rates of the combined endpoints of any (aRR 1.48, 95% CI 1.15–1.91); and moderate or severe (aRR 1.68, 95% CI 1.13–2.50), but not severe (aRR 0.63, 95% CI 0.27–1.45) GI events in the infants prescribed ibuprofen compared with those prescribed acetaminophens alone (Table 7). The effect sizes were broadly similar in the between and within subjects’ analyses in the full sample. In the subgroup who received acetaminophen or ibuprofen but not both, the within subject effects were higher, but due to the small numbers CIs were broad (Table 8). Overall, the data points to an increased incidence of mild and moderate GI adverse events in the ibuprofen treated group.

**Table 6:** Hypothesis 2 at 14-day follow up.

	Event Overall		Ibuprofen +/-acetaminophen		Acetaminophen only		Crude RR	Between subjects’ analysis Adjusted RR Age, PMHx	Within subjects’ analysis Adjusted OR Age, PMHx
Sample N 167,523	N events	Incidence ratio per 10,000 person-days	n	Incidence ratio per 10,000 person-days	n	Incidence ratio per 10,000 person-days			
**GI adverse events**									
Mild, moderate, or severe	2955	14.07 (13.52 14.53)	191	13.95 (11.88 15.78)	2764	14.04 (13.53 14.57)	0.98 (0.84 1.13)	1.02 (0.88 1.18)	1.53 (1.05 2.22)
Moderate, or severe	1,699	8.06 (7.69 8.45)	120	8.6 (7.19 10.29)	1579	8.02 (7.63 8.60)	1.07 (0.89 1.29)	1.00 (0.83 1.21)	1.51 (0.83 2.75)
Severe	291	1.38 (1.23 1.55)	19	1.36 (0.87 2.14)	272	1.38 (1.23 1.56)	0.99 (0.62 1.57)	0.90 (0.55 1.48)	0.42 (0.12 1.44)
**Renal adverse events**									
Any impairment	65	0.31 (0.24 0.39)	14	1.00 (0.59 1.69)	51	0.26 (0.20 0.34)	3.88 (2.14 7.00)	1.38 (0.47 4.01)	0.99 (0.38 2.64)
Acute impairment or failure	44	0.21 (0.16 0.28)	7	0.51 (0.24 1.05)	37	0.19 (0.13 0.26)	2.67 (1.19 5.99)	1.56 (0.31 7.81)	0.56 (0.03 9.86)

The analysis includes only those who received their first ibuprofen before six months of age and calculates the risk based on 14-day follow up only. CI; confidence interval, RR; rate ratio, OR; odds ratio, between-subjects’ analysis uses survival analysis with Mantel-Haenszel estimate of the RR, within subjects’ analysis uses a panel logistic regression fixed effects analysis, PMHx; past medical history.

**Table 7:** Hypothesis 2 at six-month follow up.

	Events overall		Ibuprofen +/- acetaminophen		Acetaminophen only		Crude RR	Between subjects’ analysis Adjusted RR Age, PMHx	Within subjects’ analysis Adjusted OR Age, PMHx
Sample N 167,523	N events	Incidence ratio per 10,000 person-days	n	Incidence ratio per 10,000 person-days	n	Incidence ratio per 10,000 person-days			
**GI adverse events**									
Mild, moderate, or severe	5,773	2.19 (2.14 2.25)	433	2.39 (2.18 2.68)	5340	2.22(2.16 2.28)	1.08 (0.98 1.19)	1.25 (1.13 1.38)	1.48 (1.15 1.91)
Moderate, or severe	3,397	1.31 (1.27 1.36)	277	1.72 (1.59 1.86)	3,120	1.53(1.36 1.72)	1.18 (1.04 1.33)	1.24 (1.09 1.40)	1.68 (1.13 2.50)
Severe	570	0.22 (0.20 0.24)	35	0.19 (0.14 0.27)	535	0.22(0.20 0.24)	0.87 (0.62 1.22)	0.96 (0.69 1.35)	0.63 (0.27 1.45)
**Renal adverse events**									
Any impairment	141	0.05 (0.04 0.06)	24	0.13 (0.09 0.20)	117	0.05 (0.04 0.06)	2.72 (1.78 4.23)	1.26 (0.57 2.79)	1.84 (0.66 5.19)
Acute impairment or failure	77	0.03 (0.02 0.04)	11	0.06 (0.03 0.11)	66	0.03 (0.02 0.03)	2.21 (1.17 4.19)	2.3 (0.83 6.42)	3.09 (0.50 19.08)

The analysis includes only those who received their first ibuprofen before six months of age. This table is based on six months follow up. CI; confidence interval, RR; rate ratio, OR; odds ratio, between-subjects’ analysis uses survival analysis with Mantel-Haenszel estimate of the RR, within subjects’ analysis uses a panel logistic regression fixed effects analysis, PMHx; past medical history.

**Table 8:** Hypothesis 2 at six-month follow up.

	Overall		Ibuprofen only		Acetaminophen only		Crude RR	Between subjects’ analysis Adjusted RR Age, PMHx	Within subjects’ analysis Adjusted OR Age, PMHx
Sample N 138,299	N events	Incidence ratio per 10,000 person-days	n	Incidence ratio per 10,000 person-days	n	Incidence ratio per 10,000 person-days			
**GI adverse** **events**									
Mild, moderate, or severe	4,666	2.16 (2.10 2.22)	58	2.87 (1.61 2.69)	4,608	2.16 (2.10 2.23)	0.962 (0.74 1.25)	1.25 (0.96 1.62)	3.71 (1.36 10.17)
Moderate, or severe	2,762	1.28 (1.23 1.33)	50	1.79 (1.36 2.37)	2712	1.27 (1.23 1.32)	1.41 (1.07 1.87)	1.40 (1.07 1.84)	4.31 (1.11 16.70)
Severe	477	0.22 (0.20 0.24)	6	0.22 (0.10 0.48)	471	0.22 (0.20 0.24)	0.97 (0.44 2.18)	1.07 (0.50 2.26)	0.95 (0.09 10.37)
**Renal adverse events**									
Any impairment	105	0.05 (0.04 0.06)	0	0	105	0.05 (0.04 0.06)	0	0	Not estimable
Acute impairment or failure	61	0.03 (0.02 0.04)	0	0	61	0.03 (0.02 0.04)	0	0	Not estimable

The analysis includes only those who received their first ibuprofen before six months of age and excludes infants who received acetaminophen in addition to ibuprofen. This table is based on six months follow up. CI; confidence interval, RR; rate ratio, OR odds ratio, between-subjects’ analysis uses survival analysis with Mantel-Haenszel estimate of the RR, within subjects’ analysis uses a panel logistic regression fixed effects analysis, PMHx; past medical history.

#### Renal events

Renal impairment occurred in 141 infants who received ibuprofen or acetaminophen before six months of age. At both 14-day (Table 6) and six-month (Table 7) follow up we observed increased unadjusted (RR 2.72, 95% CI 1.78–4.23) but not adjusted (for age and prior renal impairment) (aRR 1.84, 95% CI 0.66–5.19) analyses showed increased incidence ratios of renal adverse effects. In the subgroup who received acetaminophen or ibuprofen but not both we observed very few outcomes (Table 8).

## Discussion

Our work shows that ibuprofen is not inherently associated with more side effects in children younger than six months compared with those older than six months, but that compared to acetaminophen alone ibuprofen is associated with more adverse effects in infants younger than six months. We did not see this effect in severe adverse events, possibly because we studied only prescription-based rather than parent-initiated OTC treatment. Our data for isolated ibuprofen prescription rather than ibuprofen and acetaminophen co-prescription is limited. While the between-patient analyses showed little difference between the ibuprofen alone and ibuprofen-plus-acetaminophen groups, the within subject point estimates were higher in the ibuprofen alone group (Table 8). This could be a result of the small numbers receiving ibuprofen alone, but it could also reflect the use of fewer doses of ibuprofen when it was prescribed with acetaminophen than when it was prescribed alone.

We observed a lower RR of GI adverse events in infants younger than six months compared with those older than six months of age. This could reflect additional caution in prescribing ibuprofen in the younger age group rather than ibuprofen being safer in infants younger than six months of age. While permitting a (conceptual) two-sided null would allow us to accept that ibuprofen is safer in younger rather than older infants, we feel (a conceptual (all reported p-values here are two-sided) one-sided null) concluding that ibuprofen is not less safe between these groups is more robust to potential differential levels of physician caution in prescribing between the two age groups.

Our results are similar to those of most but not all other studies and support the findings of a recent comprehensive review on the topic by Ziesenitz *et al*.[24] In *Lesko and Mitchell* a comparison of ibuprofen to acetaminophen for fever in older children, protocol violations led to 319 infants younger than six months receiving ibuprofen. None had serious adverse effects.[14] In the Children’s Analgesic Medicine Project (CAMP), a prospective observational study of 41,810 children aged 1 month to 18 years; with fever or pain, 14,281 were less than 2 years old and received either ibuprofen or acetaminophen. No cases of anaphylaxis, Reye’s syndrome, GI bleeding, or necrotizing fasciitis were reported in either cohort.[25] None of the 4 deaths observed was attributed to ibuprofen according to the authors. Unfortunately, the CAMP study does not provide subgroup information for infants younger than six months and our attempts to obtain this information from the study sponsor failed. In the Boston Fever study (n = 83,915 of whom 27,065 were less than 24 months) there were only four cases of GI bleeding and none of Reye’s syndrome.[14] Our results are not directly comparable but adverse events are similarly low.

Our results conflict in part with those of the CAMP study and Boston Fever Study with respect to adverse renal effects.[14, 25] Neither study reported a single case of renal adverse events from ibuprofen use. Similarly, *Lesko and Mitchell* also found no difference in serial blood urea nitrogen or serum creatinine levels in 288 children (median age 22 months in the ibuprofen group) treated with ibuprofen and/or acetaminophen for fever.[26] A study using high dose ibuprofen in children with cystic fibrosis similarly reported no untoward renal events.[27]

Other authors have found associations with NSAID use and renal injury in children with dehydration and odds ratios comparable to the unadjusted rate ratio we observed.[28] A retrospective study of 1,015 children younger than 18 years with acute kidney injury attributed 2.7% of cases to NSAID use.[29] Previous studies and meta analyses arguing for ibuprofen’s safety have typically been prospective whereas those arguing against ibuprofen’s safety have typically been retrospective or a case-control design.[30] [28] NSAID induced nephropathy presents as one or a combination of: renal vascular constriction, interstitial nephritis, minimal change glomerulonephropathy, or papillary necrosis.[31] These complications are usually associated with prolonged NSAID use.[31] Adjusting for pre-existing renal insufficiency, we found no increase in renal adverse events with ibuprofen use.

The association between NSAIDs and necrotizing fasciitis, particularly in association with varicella, is controversial with even large studies being inconclusive due to the rarity of the combination.[32] We had only five cases of infants in our dataset who developed necrotizing fasciitis; none followed documented ibuprofen exposure.

The association between Reye’s Syndrome and aspirin[11, 33] and the decline of Reye’s syndrome following FDA advice to avoid aspirin use for febrile illnesses in children[34] has led to fears that COX-2 inhibiting NSAIDS may also pose a risk. However, there is little evidence associating ibuprofen to Reye’s syndrome and we found no cases.

We assume that the prescription writers considered the patients’ known risks for ibuprofen use. This assumption seems to be supported by the apparently slightly protective effect for ibuprofen (Table 4). This is important because it may mean our data are supportive of prescription rather than OTC use of ibuprofen in this age group.

### Limitations

Our findings best describe the common clinical scenario where infants receive both acetaminophen and ibuprofen. Our methodology could not detect if parents chose to give only one agent instead of both prescribed, nor could we detect any other element of compliance beyond noting that the prescription was filled. Our methodology is based on passive reporting–parents had to attend their physician for a symptom. There is no reason to believe that this would bias our results in favor of finding safety over harm because the same method was applied to those older than and younger than six months. We speculate that parents may be more likely to bring a younger infant to the doctor for a given set of symptoms. If true, this may bias our results against finding safety in the younger age group. Clinically undetected/undiagnosed/unrecorded events will not be detected by our design.

Ibuprofen is available OTC and parents could ignore the FDA mandated OTC ibuprofen labeling specifically warning against use in infants younger than six months. In addition, without a prescription there is a financial barrier, whereas there is no financial barrier for *Medi-Cal* patients to obtain a prescription; this makes additional unrecorded ibuprofen exposure less likely. Even nominal copays dramatically decrease Medicaid prescription fulfillment rates.[35] In our practice, we have observed enormous resistance among parents to being denied their right to a prescription for antipyretics. However, some parents may purchase OTC ibuprofen (generally at modest cost) out-of-pocket to avoid opportunity costs of a doctor’s visit and inconvenience. Ibuprofen obtained OTC carries an instruction label to not administer it to infants younger than six months and is without dosing instruction for these infants. There is no such age restriction for acetaminophen.

Our estimate of prescription numbers is semi-quantitative as it describes filled prescriptions for the drug availability rather than proven use. Because serious side effects are rare, very large numbers of children need to be studied to detect with high statistical power those that do occur; prospective methods involving medication bottle measurements (especially, randomized controlled trials) are neither practical nor financially feasible. Therefore, given the usual well-known indications of antipyresis and analgesia our findings should be taken to apply only to short-term use. Diagnosis of GI adverse events was clinical; consequently, misattribution could occur if the physician knew, as he/she generally would, that the child had previously been prescribed ibuprofen. This is particularly the case for the mild category of GI events. This category of event could easily capture patients presenting in the early stages of gastroenteritis where vomiting but not diarrhea has occurred. This bias would tend to cause excessive attribution of symptoms to ibuprofen. The moderate and severe categories of GI event are much less susceptible to such bias and are clinically much more important.

It is also possible that infants receiving a prescription for ibuprofen are judged sicker than those who do not, thereby increasing their risk of subsequent medical attendance. It is unclear that this would increase the incidence rate of the outcomes we specifically sought.

Administrative datasets are not designed for research. Clinical details especially were very limited. Some of the potential adverse events probably reflected other illnesses and were unrelated to prior ibuprofen use, potentially biasing our results against finding safety. Finally, our results should neither be extrapolated beyond the general pediatric population, nor to other NSAIDs, even other propionic acids.[36]

The primary advantage of our methodology was ensuring a sample size large enough to detect rare but important outcomes in real world settings.[37] Medicaid data has previously successfully been used to study drug safety outcomes.[18, 19, 38] The sample sizes required to detect these adverse events with precise confidence intervals make a prospective study infeasible. As a result, researchers must choose between noisy imperfect estimates of ibuprofen safety or none.

## Conclusions

In conclusion, we did not observe significantly increased adverse GI or renal events in infants younger than six months compared with those older than six months who were prescribed ibuprofen. We did observe increased rates of some potential adverse events in infants younger than six months who were prescribed both acetaminophen and ibuprofen, compared with infants who were prescribed acetaminophen alone.

## Supporting information

S1 File Letter from the food and drug administration refusing over-the-counter labelling for ibuprofen for children younger than six months of age. (PDF)

## References

[1] Davies N. Clinical pharmacokinetics of ibuprofen. Clinical Pharmacokinetics. 1998;34(2):101–54. doi: 10.2165/00003088-199834020-00002 9515184 10.2165/00003088-199834020-00002

[2] Engström Ruud L, Wilhelms DB, Eskilsson A, Vasilache AM, Elander L, Engblom D, et al Acetaminophen reduces lipopolysaccharide-induced fever by inhibiting cyclooxygenase-2. Neuropharmacology. 2013;71(0):124–9. doi: 10.1016/j.neuropharm.2013.03.012 23545161 10.1016/j.neuropharm.2013.03.012

[3] Vane RJ, Flower JR. Inhibition of prostaglandin synthetase in brain explains the anti-pyretic activity of paracetamol (4-acetamidophenol). Nature. 1972;240(5381):410–1. 4564318 10.1038/240410a0

[4] Sullivan JE, Farrar HC. Section on Clinical Pharmacology and Therapeutics, and Committee on Drugs. Fever and antipyretic use in children. Pediatrics. 2011;127(3):580–7. doi: 10.1542/peds.2010-3852 21357332 10.1542/peds.2010-3852

[5] Berezin SH, Bostwick HE, Halata MS, Feerick J, Newman LJ, Medow MS. Gastrointestinal bleeding in children following ingestion of low-dose ibuprofen. Journal of Pediatric Gastroenterology and Nutrition. 2007;44(4):506–8 doi: 10.1097/MPG.0b013e31802d4add 17414151 10.1097/MPG.0b013e31802d4add

[6] Moghal NE, Hegde S, Eastham KM. Ibuprofen and acute renal failure in a toddler. Archives of Disease in Childhood. 2004;89(3):276–7. doi: 10.1136/adc.2002.024141 14977711 10.1136/adc.2002.024141 PMC1719837

[7] Ulinski T, Guigonis V, Dunan O, Bensman A. Acute renal failure after treatment with non-steroidal anti-inflammatory drugs. European Journal of Pediatrics. 2004;163(3):148–50. doi: 10.1007/s00431-003-1392-7 14745553 10.1007/s00431-003-1392-7

[8] Wattad A, Feehan T, Shepard FM, Youngberg G. A unique complication of nonsteroidal anti-inflammatory drug use. Pediatrics. 1994;93(4):693–693. 8134235

[9] Solomon L. Activation of latent infection by indomethacin: A report of three cases. BMJ. 1966;1(5493):961–2. 20790925 10.1136/bmj.1.5493.961 PMC1844861

[10] Holder EP, Moore PT, Browne BA. Nonsteroidal anti-inflammatory drugs and necrotising fasciitis: An update. Drug Safety. 1997;17(6):369–73. 9429836 10.2165/00002018-199717060-00003

[11] Hurwitz ES, Barrett MJ, Bregman D, Gunn WJ, Schonberger LB, Fairweather WR, et al Public health service study on Reye's syndrome and medications report of the pilot phase. New England Journal of Medicine. 1985;313(14):849–57. doi: 10.1056/NEJM198510033131403 4033715 10.1056/NEJM198510033131403

[12] Gokmen T, Erdeve O, Altug N, Oguz SS, Uras N, Dilmen U. Efficacy and safety of oral versus intravenous ibuprofen in very low birth weight preterm infants with patent ductus arteriosus. J Pediatr. 2011;158(4):549–54.e1. doi: 10.1016/j.jpeds.2010.10.008 21094951 10.1016/j.jpeds.2010.10.008

[13] Van Overmeire B, Smets K, Lecoutere D, Van de Broek H, Weyler J, De Groote K, et al A Comparison of ibuprofen and indomethacin for closure of patent ductus arteriosus. New England Journal of Medicine. 2000;343(10):674–81. doi: 10.1056/NEJM200009073431001 10974130 10.1056/NEJM200009073431001

[14] Lesko SM, Mitchell AA. The safety of acetaminophen and ibuprofen among children younger than two years old. Pediatrics. 1999;104(4):e39 doi: 10.1542/peds.104.4.e3 10506264 10.1542/peds.104.4.e39

[15] Katz L. Letter to McNeill Healthcare. Letter. From Linda Katz, Federal Drug Administration Rockville, MD. to Vivian Chester, Mc Neill Healthcare, Fort Washington, PA 1999. available from the FDA at https://www.accessdata.fda.gov/drugsatfda_docs/nda/98/20-603S001_Ibuprofen_corres.pdf, https://www.accessdata.fda.gov/drugsatfda_docs/nda/98/20-603S001_Ibuprofen_medr_Pr1.pdf, and https://www.accessdata.fda.gov/drugsatfda_docs/nda/9820-603S001_Ibuprofen_medr_Pr2.pdf (Accessed 5-15-2018) [combined PDF of these is included in the appendices.]

[16] Joint Formulary Committee. British National Formulary for Children. London: BMJ Group and Pharmaceutical Press. United Kingdom: British Medical Journal Group Ltd.; 2012 p. 503.

[17] Leonard CE, Brensinger CM, Nam YH, Bilker WB, Barosso GM, Mangaali MJ, et al The quality of Medicaid and Medicare data obtained from CMS and its contractors: implications for pharmacoepidemiology. BMC Health Serv Res. 2017;17(1):304 doi: 10.1186/s12913-017-2247-7 28446159 10.1186/s12913-017-2247-7 PMC5406992

[18] Winterstein AG, Gerhard T, Kubilis P, Saidi A, Linden S, Crystal S, et al Cardiovascular safety of central nervous system stimulants in children and adolescents: population based cohort study. BMJ. 2012;345:e4627 doi: 10.1136/bmj.e4627 22809800 10.1136/bmj.e4627 PMC3399772

[19] Chen CY, Bussing R, Hartzema AG, Shuster JJ, Segal R, Winterstein AG. Stimulant use following the publicity of cardiovascular safety and the introduction of patient medication guides. Pharmacoepidemiol Drug Saf. 2016;25(6):678–86. doi: 10.1002/pds.3894 26597624 10.1002/pds.3894

[20] Clayton D, Hills H. Analysis of follow-up studies. Stata Technical Bulletin. 1995; 5: 219–27.

[21] Clayton D, Hills M. Analysis of follow-up studies with Stata 5.0. Stata Technical Bulletin Reprints. 1997;7:253–68.

[22] Statacorp LLC. Stata Longitudinal-Data/Panel-Data Reference Manual. College Station, TX: Statacorp LLC; 1985–2017. p. 563.

[23] Schisterman EF, Cole SR, Platt RW. Overadjustment bias and unnecessary adjustment in epidemiologic studies. Epidemiology. 2009;20(4):488–95. doi: 10.1097/EDE.0b013e3181a819a1 19525685 10.1097/EDE.0b013e3181a819a1 PMC2744485

[24] Ziesenitz VC, Zutter A, Erb TO, van den Anker JN. Efficacy and Safety of Ibuprofen in Infants Aged Between 3 and 6 Months. Paediatr Drugs. 2017 doi: 10.1007/s40272-017-0235-3 28516288 10.1007/s40272-017-0235-3

[25] Ashraf E; Ford L. LGCS. Safety profile of ibuprofen suspension in young children. InflammoPharmacology. 1999;7(3):219–25. doi: 10.1007/s10787-999-0005-0 17638093 10.1007/s10787-999-0005-0

[26] Lesko SM, Mitchell AA. Renal Function After Short-term Ibuprofen Use in Infants and Children. Pediatrics. 1997;100(6):954–7. doi: 10.1542/peds.100.6.954 9374563 10.1542/peds.100.6.954

[27] Lahiri T, Guillet A, Diehl S, Ferguson M. High-dose ibuprofen is not associated with increased biomarkers of kidney injury in patients with cystic fibrosis. Pediatr Pulmonol. 2014;49(2):148–53. doi: 10.1002/ppul.22795 23532925 10.1002/ppul.22795

[28] Balestracci A, Ezquer M, Elmo ME, Molini A, Thorel C, Torrents M, et al Ibuprofen-associated acute kidney injury in dehydrated children with acute gastroenteritis. Pediatr Nephrol. 2015 doi: 10.1007/s00467-015-3105-7 25895445 10.1007/s00467-015-3105-7

[29] Misurac JM, Knoderer CA, Leiser JD, Nailescu C, Wilson AC, Andreoli SP. Nonsteroidal anti-inflammatory drugs are an important cause of acute kidney injury in children. J Pediatr. 2013;162(6):1153–9, 9.e1. doi: 10.1016/j.jpeds.2012.11.069 23360563 10.1016/j.jpeds.2012.11.069

[30] Pierce CA, Voss B. Efficacy and safety of ibuprofen and acetaminophen in children and adults: a meta-analysis and qualitative review. Ann Pharmacother. 2010;44(3):489–506. Epub 2010/02/11. doi: 10.1345/aph.1M332 20150507 10.1345/aph.1M332

[31] Henrich WL, Agodoa LE, Barrett B, Bennett WM, Blantz RC, Buckalew VM Jr, et al Analgesics and the kidney: Summary and recommendations to the Scientific Advisory Board of the National Kidney Foundation from an ad hoc Committee of the National Kidney Foundation. American Journal of Kidney Diseases. 1996;27(1):162–5. doi: 10.1016/S0272-6386(96)90046-3 8546133 10.1016/s0272-6386(96)90046-3

[32] Choo PW, Donahue JG, Platt R. Ibuprofen and skin and soft tissue superinfections in children with varicella. Ann Epidemiol. 1997;7(7):440–5. doi: 10.1016/S1047-2797(97)00040-9 9349910 10.1016/s1047-2797(97)00040-9

[33] Hurwitz ES, Barrett MJ, Bregman D, Gunn WJ, Pinsky P, Schonberger LB, et al Public Health Service study of Reye's syndrome and medications. Report of the main study. JAMA. 1987;257(14):1905–11. 3820509

[34] Belay ED, Bresee JS, Holman RC, Khan AS, Shahriari A, Schonberger LB. Reye's Syndrome in the United States from 1981 through 1997. New England Journal of Medicine. 1999;340(18):1377–82. doi: 10.1056/NEJM199905063401801 10228187 10.1056/NEJM199905063401801

[35] Hartung DM, Carlson MJ, Kraemer DF, Haxby DG, Ketchum KL, Greenlick MR. Impact of a Medicaid copayment policy on prescription drug and health services utilization in a fee-for-service Medicaid population. Med Care. 2008;46(6):565–72. doi: 10.1097/MLR.0b013e3181734a77 18520310 10.1097/MLR.0b013e3181734a77

[36] Skúladóttir HM, Andrésdóttir MB, Hardarson S, Árnadóttir M. The acute flank pain syndrome: a common presentation of acute renal failure in young males in Iceland. NDT Plus. 2010;3(5):510–1. doi: 10.1093/ndtplus/sfq117 25984076 10.1093/ndtplus/sfq117 PMC4421712

[37] Crystal S, Akincigil A, Bilder S, Walkup JT. Studying prescription drug use and outcomes with medicaid claims data: strengths, limitations, and strategies. Med Care. 2007;45(10 Supl 2):S58–65. doi: 10.1097/MLR.0b013e31805371bf 17909385 10.1097/MLR.0b013e31805371bf PMC2486436

[38] Sauer B, Shinogle J, Xu W, Samore M, Nebeker J, Liu Z, et al. Medicare Prescription Drug Data Development: Methods for improving patient safety and pharmacovigilance using observational Data. Effective Health Care Research Report No. 6. Rockville MD: Agency for Healthcare Research and Quality. 2008. Report No. 6. Available at https://effectivehealthcare.ahrq.gov/topics/patient-safety-databases-prescriptions/research/. Accessed 5/15/2018.

