# Supplementary material for: Safety of ibuprofen in infants younger than six months: A retrospective cohort study
Source: PLoS One. 2018 Jun 28;13(6):e0199493. doi: 10.1371/journal.pone.0199493 (PMC6023220; doi:10.1371/journal.pone.0199493)
Supplement: S1 File — (PDF) [file pone.0199493.s001.pdf]

20603S003

NDA 20-603/S-003

APR 1 5 1999

McNeil Consumer Healthcare
Attention: Vivian A. Chester
7050 Camp Hill Road
Fort Washington, PA 19034-2299

Dear Ms. Chester:

Please refer to your supplemental new drug application dated and received June 15, 1998, submitted under section 505(b) of the Federal Food, Drug, and Cosmetic Act for Infant's Motrin (ibuprofen oral suspension) Concentrated Drops, 50 mg/1.25 mL.

We acknowledge receipt of your correspondences dated April 14 and 15, 1999.

This supplemental new drug application provides for the the expanded use of Infant's Motrin (ibuprofen oral suspension) Concentrated Drops, 50 mg/1.25 mL to include dosing instructions for children 6 months to 23 months of age.

The user fee goal (10 months) for this supplemental new drug application is April 15, 1999.

We have completed the review of this supplemental new drug application and have concluded that adequate information has been presented to demonstrate that the drug product is safe and effective for use as recommended in the enclosed agreed upon labeling text. Accordingly, the supplemental new drug application is approved effective on the date of this letter.

The final printed labeling (FPL) must be identical to the enclosed labeling text. Marketing the product with FPL that is not identical to the approved labeling text may render the product misbranded and an unapproved new drug.

Please submit 20 copies of the FPL as soon as it is available, in no case more than 30 days after it is printed. Please individually mount ten of the copies on heavy-weight paper or similar material. For administrative purposes, this submission should be designated "FPL for approved supplement NDA 20-603/S-003." Approval of this submission by FDA is not required before the labeling is used.

If additional information relating to the safety or effectiveness of this drug product becomes available, revision of the labeling may be required.

Please submit three copies of the introductory promotional materials that you propose to use for

this product. All proposed materials should be submitted in draft or mock-up form, not final print. Please submit one copy to the Division of Over-the-Counter Drug Products and two copies of both the promotional materials and the labeling directly to:

Food and Drug Administration
Division of Drug Marketing, Advertising, and Communications, HFD-40
5600 Fishers Lane
Rockville, Maryland 20857

If a letter communicating important information about this drug product (i.e., a "Dear Health Care Practitioner" letter) is issued to physicians and others responsible for patient care, we request that you submit a copy of the letter to this NDA and a copy to the following address:

MEDWATCH, HF-2
FDA
5600 Fishers Lane
Rockville, MD 20857

As of April 1, 1999, all applications for new active ingredients, new dosage forms, new indications, new routes of administration, and new dosing regimens are required to contain an assessment of the safety and effectiveness of the product in pediatric patients unless this requirement is waived or deferred (63 *FR* 66632). We note that you have fulfilled the pediatric study requirement at this time.

Please submit one market package of the drug product when it is available.

We remind you that you must comply with the requirements for an approved NDA set forth under 21 CFR 314.80 and 314.81.

If you have any questions regarding this application, please contact Kerry Rothschild, Esq., Regulatory Project Manager, at (301) 827-2222.

Sincerely yours,

[signature]

Linda M. Katz, M.D., M.P.H.
Deputy Director 4/15/99
Division of Over-the-Counter Drug Products
Office of Drug Evaluation V
Center for Drug Evaluation and Research

Enclosure

FINAL PRINTED LABELING HAS NOT BEEN SUBMITTED TO THE FDA

DRAFT LABELING IS **NO LONGER** BEING SUPPLIED SO AS TO ENSURE ONLY CORRECT AND CURRENT INFORMATION IS DISSEMINATED TO THE PUBLIC.

THIS SECTION WAS DETERMINED **NOT** TO BE RELEASABLE

6 pages

Draft Labeling

Copy

MEDICAL OFFICER REVIEW
Division of Over-The-Counter Drug Products

NDA #: 20-603, SE5-~~005~~ 003
NAME: Children's MOTRIN® (ibuprofen oral suspension) Drops, 50 mg/1.25 mL
SPONSOR: McNeil Consumer Products Company
7050 Camp Hill Road
Fort Washington, PA 19034-2299
Tel.:(215)233-7000
TYPE OF SUBMISSION: Commercial Pharmaceutical
DATE OF SUBMISSION: June 15, 1998 **CDER:** June 15, 1998
DATE OF REVIEW: March 15, 1999
REVIEWER: Rosemarie Neuner, MD, MPH
CSO: Mr. Kerry Rothschild, JD

Introduction

Ibuprofen is a propionic acid derivative that belongs to the nonsteroidal anti-inflammatory class of drugs (NSAIDs). A suspension formulation of ibuprofen (100 mg/5 mL) has been marketed in the United States since 1989 by McNeil Consumer Products for use in children (age 6 months and older) as a prescription drug, under the trade names, Pedia-Profen and Children's MOTRIN® Suspension. On June 10, 1995 Children's MOTRIN® (ibuprofen oral suspension) Drops, 50 mg/1.25 mL was approved by the U.S. Food and Drug Administration for marketing as an over-the-counter (OTC) drug product for the temporary relief of fever and pain in children 2-3 years of age. In June 1998, the sponsor of this product, McNeil Consumer Products, submitted a request to the agency for a pediatric exclusivity claim which was subsequently granted. The sponsor has now submitted this efficacy supplement for agency review in which they request the lowering of the currently approved group age range from two to three years of age down to two months of age for this product.

In support of this change in the product's dosing age range, the sponsor has submitted the results of a new subgroup analysis of data generated from 27,000 children less than 2 years of age who participated in the actual use drug -safety trial, the Boston University Fever Study, which evaluated the safety profile of Children's MOTRIN® as an antipyretic agent. (Note: This was the pivotal safety study that supported the approval for the sponsor's NDA 20-516 Children's MOTRIN® Ibuprofen Oral Suspension 100 mg/5 mL in 1995. It also served as the supportive safety study in the approval of the sponsor's other NDA 20-603 Children's MOTRIN® Ibuprofen Drops 50 mg/1.25 mL in 1996.) In addition, the sponsor has included the results of 21 clinical trials where children 2 years old and younger participated as study subjects, in addition to 4 published pharmacokinetic (PK) studies involving children ages 2 months to 2 years. The results from these PK studies are discussed in the PK section of this SNDA review by Dr. E. Dennis Bashaw, FDA Division of Pharmacokinetics (HFD-880).

Since prescription ibuprofen is currently approved for use in infants age 6 months and older, the major regulatory issue to be answered by this application is it

safe for OTC ibuprofen to be used in the pediatric age group 2 months and older at the doses proposed by the sponsor of this supplement. This review will therefore concentrate on the drug's safety profile in this targeted age group.

Efficacy

In support of ibuprofen's efficacy in the targeted pediatric age group of 2 months to 2 years, the sponsor performed an extensive search of the worldwide literature. This search yielded 23 articles and 6 abstracts which described the results of 21 randomized, controlled antipyretic (16) and analgesia (5) trials which evaluated ibuprofen in children ages less than 2 years of age. A complete listing of these articles and abstracts, and their trial summaries written by the sponsor can be found in the following sponsor's tables, Tables 8-10 and 8-12, in Attachment I.

A total of 2,032 febrile children between the ages of 2 months to 13 years participated in the antipyretic studies. (See the following sponsor's table, Table 8-10, found in Appendix I.) Of these 16 studies, 2 were placebo-controlled trials. The other 14 studies compared ibuprofen to active controls such as acetaminophen or aspirin. Five (5) out of the 16 studies were single-dose studies while the remaining 11 trials were multi-dose studies of ibuprofen. These 16 trials tested doses of ibuprofen in the range of 0.5 mg/kg to 10 mg/kg. All 16 studies showed that ibuprofen at the doses tested, with the exception of the lowest dosing range, was an efficacious antipyretic agent in the populations tested. (Refer to Table 8-10 found in Attachment I at the end of this review.)

A total of 504 children between the ages of 6 months to 14 years participated in the 4 postoperative and 1 otitis media analgesic studies. (See the following sponsor's table, Table 8-12, located in Attachment I.) Two out of the 5 trials were placebo-controlled studies, 2 were placebo- and active- controlled studies, and 1 study evaluated ibuprofen as a single-agent with codeine used for rescue pain. Three of the 5 studies evaluated multi-doses while 2 were single-dose trials. The dose range of ibuprofen used in these analgesia studies ranged from 5 mg/kg to 13 mg/kg. All 5 studies showed that ibuprofen at the doses tested was comparable to acetaminophen or more efficacious than placebo in the control of pain in the patients studied.

The sponsor created the following 2 tables, Sponsor's Tables 1 and 2, below, to show how many studies in this collection included study subjects from the targeted pediatric age group. With the exception of one randomized, double-blind, actively controlled antipyretic trial which compared ibuprofen 7.5 mg/kg to acetaminophen 10 mg/kg in 154 children aged 6-months to 5-years, all of the remaining studies used descriptive statistics (i.e., mean, standard deviation, and range) in discussing the age of the subjects who participated in the studies. Thus, it is impossible to know how many children less than 2 years of age actually participated in these studies.

Sponsor's Table 1 - Age of the Youngest Patient Included in Published Efficacy Antipyretic Trials

Age	Number of Studies	Literature Reference [one study]
2-3 months	6	[8] [19,49] [20] [25] [26] [27]
4-11 months	9	[6] [7] [10,13] [11,17] [12] [15,22] [14,16,24] [18,21] [23]
12-23 months	1	[9]

Sponsor's Table 2 - Age of the Youngest Patient Included in Published Efficacy Analgesic Trials

Age	Number of Studies	Literature Reference [one study]
2-3 months	0	None
4-11 months	1	[30]
12-23 months	4	[28] [29,33] [31] [32]

Since these studies are not proprietary studies, and their actual case reports and data sets were not included in this submission for review, they can only be considered supportive of the already established efficacy profile of ibuprofen in children ≤ 2 years old. As previously discussed, ibuprofen as a prescription drug is considered to be efficacious in the sponsor's requested pediatric target age group for this submission. Thus, these studies are being included in this review at this time for completeness and reference.

Medical Reviewer's Comments: All 21 studies showed that ibuprofen was an efficacious agent for the indications studied when compared to placebo and other recognized antipyretic and analgesic agents. Thus, these studies can be used in support of ibuprofen's already recognized effectiveness as an antipyretic and analgesic agent in the sponsor's requested targeted age group.

APPEARS THIS WAY ON ORIGINAL

Safety

As discussed in the preceding introduction, the focus of this review is to determine whether ibuprofen is safe to be used as an OTC agent in the sponsor's requested targeted pediatric age group of 2-months to 2-years. In support of this product's safety profile the sponsor has submitted for review the following safety data for children less than 2-years of age:

1. The Boston University Fever Study subgroup analysis of children less than 2-years of age.
2. McNeil CPC controlled clinical trial data on subjects ≤ 2-years of age enrolled on or after November 17, 1993 and treated with ibuprofen.
3. McNeil CPC Spontaneous Reporting System for McNeil CPC ibuprofen products in children ≤ 2-years of age for the time period November 17, 1993 through October 2, 1997, including serious reports in the published literature.
4. FDA Spontaneous Reporting System for all ibuprofen products in children ≤ 2-years of age for the time period November 1, 1993 through August 25, 1997. (Note: Adverse events reported through McNeil Spontaneous Reporting System are not included here.)
5. Published randomized controlled clinical trials and human pharmacokinetic studies of ibuprofen products for the years 1966 through October 1997 that reported including children ≤ 2-years of age.
6. AAPCC TESS ibuprofen data from the years 1994 through 1996 for children ≤ 2-years of age. (The 1997 report was not yet available.)

The sponsor has compiled the following summary table, Table 8-13, which outlines the total number of serious adverse events that have been reported to have occurred in children < 2-years of age since November 1993 from the above submitted safety data base. (See sponsor's table, Table 8-13, below.)

The cornerstone of this safety data base for children (see sponsor's table, Table 8-13, below) is generated from the actual use safety study, the Boston Fever Study. Although this study was reviewed by the agency in support of a regulatory action for NDA 20-516 Children's MOTRIN® Ibuprofen Oral Suspension 100 mg/5 mL in 1995, the sponsor has submitted for review a new subcohort analysis of the 27,065 children < 2 years of age who participated in this study which compares the incidence of adverse events that occurred during the trial in this group to that of subjects age ≥ 2 years. This study will be discussed first followed by reviews of the other safety data as listed above.

**APPEARS THIS WAY
ON ORIGINAL**

Table 8-13. Summary of Ibuprofen Safety Data For Children Less Than Two Years of Age (data since November 1993, except where noted otherwise)

**Boston University Fever Study (children < two years of age)**	(Total Patients)	27,065
Total Ibuprofen Exposures		17,938
Hospitalizations for events of primary interest		3
Other hospitalizations (excluding deaths)		258
Deaths		0
**McNeil CPC Controlled Clinical Studies (children < two years of age)**		
Antipyretic Study	(Total Patients)	1
Total Ibuprofen Exposures[1]		1
Reports of AEs with serious outcomes (excluding deaths)[2]		0
Deaths		0
**Commercial Marketing Experience**		
• Dosage Units (50 mg) Shipped: OTC Children's Motrin Ibuprofen Products (all ages)		
• Total Prescriptions Filled: Pediatric Ibuprofen Suspension and Drops (all ages)		
• Total Physician Recommendations for Pediatric Ibuprofen Products (estimate for children < two years of age)		
McNeil CPC Drug Safety Reporting System (pediatric ibuprofen products) (children < two years of age)		
Total Ibuprofen Reports		314
Reports of AEs with serious outcomes (excluding deaths)[2]		7
Deaths		2
FDA Spontaneous Reporting System (pediatric, adult and unknown ibuprofen products) (children < two years of age)		
Total Ibuprofen Reports (excludes McNeil CPC reports)		72
Reports of AEs with serious outcomes (excluding deaths)[2]		20
Deaths		5
AAPCC TESS Database (pediatric, adult and unknown ibuprofen products) (children < two years of age)		
Total Ibuprofen Exposure Cases (1994 through 1996)		17,635
Moderate or major outcomes (excluding deaths)[3]		29
Deaths		0
**Published Randomized Controlled Studies (inclusive of, but not limited to, children < two years of age[4]) (first study published in 1976)**		
Antipyretic Studies	(Total Patients)	2032
Total Ibuprofen Exposures		974
Serious AEs (excluding deaths)[5]		0
Deaths		0
Analgesic Studies	(Total Patients)	504
Total Ibuprofen Exposures		213
Serious AEs (excluding deaths)[5]		0
Deaths		0
Pharmacokinetic/Pharmacodynamic Studies	(Total Patients)	340
Total Ibuprofen Exposures		194
Serious AEs (excluding deaths)[5]		0
Deaths		0

1 One child less than two years of age was unintentionally enrolled in a McNeil CPC controlled trial of ibuprofen for which enrollment was planned for children two to 11 years of age (Protocol No. 95-516).

2 A serious outcome is defined as an adverse event that is life threatening (immediate risk of death from the reaction), requires inpatient hospitalization, prolonged hospitalization, or is permanently or severely disabling. Outcomes of death, congenital anomaly, or cancer are also considered serious.

3 Generally, a moderate outcome involves a patient who exhibits signs or symptoms as a result of the exposure which are pronounced, prolonged, or of a systemic nature; usually some form of treatment is required. Symptoms are not life-threatening and the patient has no residual disability or disfigurement. A major outcome generally involves a patient who exhibits signs or symptoms as a result of the exposure which are life-threatening or result in significant residual disability or disfigurement.

4 While overall safety information was reported, such information for children less than two years of age was not specified.

5 Serious as defined by the investigator.

1. The Boston Fever Study Subcohort Analysis of Children Less Than 2 Years of Age.

This was a 4-week, multicenter, double-blind, randomized, acetaminophen-controlled antipyretic study conducted by the Slone Epidemiology Unit of Boston University in office-based pediatric population from the continental United States. The study's objective was to assess the risk of serious adverse events such as gastrointestinal bleeding, acute renal failure, anaphylaxis and Reye syndrome associated with the use of ibuprofen in febrile children. Children between 6 months to 12 years of age weighing between 7-50 kg were recruited after presenting for a pediatric evaluation of an acute febrile illness to any one of the 1,735 pediatricians or family practitioners who participated in the trial. In order to be eligible for study entry, the children had to be able to take the study medication by mouth, and have a parent/guardian able to administer the study medication while observing and caring for them. Children who were dehydrated, unable to take medication by mouth, or with histories of hypersensitivity to acetaminophen or NSAIDS, renal or hepatic diseases, bleeding disorders, anemia, neoplasia, endocrine or metabolic problems, or peptic ulcer disease were ineligible for study entry. A total of 84,192 patients were entered into the trial out of which 83,915 patients were randomized and received 1 of the following 3 treatments: 5 mg/kg ibuprofen, 10 mg/kg ibuprofen, or 12 mg/kg acetaminophen.

Out of the total of 83,915 children entered into the study, 27,065 were < 2 years and 56,850 were ≥ 2 years of age. Demographically, the 2 age groups on comparison as well as the 3 randomized treatment groups were very similar in make up as shown in the following 2 tables, Sponsor's Tables 3 and 4, below.

Sponsor's Table 3 - Demographic Characteristics of All Participants

Characteristic	Age: < 2 years (n =27,065)	Age: ≥ 2 years (n = 56,850)
Median Age (Months)	13	59
Median Weight (kg)	10	18
Sex, %Male<br>% Female	54<br>46	50<br>50
Race, %White<br>% African-American<br>%Hispanic	81<br>7.2<br>7.2	82<br>7.3<br>6.6

Sponsor's Table 4 - Demographic Characteristics of 27,065 Participants ≤ 2 Years Old According to Treatment Group

Characteristic	Acetaminophen	Ibuprofen (5 mg/kg)	Ibuprofen (10 mg/kg)
Total Number	9,127	9,159	8,779
Median Age (Months)	14	13	13
Median Weight (kg)	10	10	10
Sex, % Male	54	54	55
Race, % White	82	81	80
% African-American	7.3	6.8	7.4
% Hispanic	6.7	7.2	7.7

Three-hundred nineteen (319) children (1.1%) ≤ 6 months of age were entered into the study despite an entry age requirement of being at least 6 months or older. Table 5 below lists the numbers of infants ≤ 6 months who participated in the study. (Note: In the official study report the sponsor states that because age was not routinely confirmed, children ≤ 6 months of age were only included in the analysis of the study data if their reported weight was between the 5th and 95th percentile for month of reported age.).

Table 5 - Age Distribution For 319 Children Younger than 6-Months of the 27,065 Participants ≤ 2 Years Old at Enrollment

Age in Months	Number	Percent
1	4	0.015%
2	13	0.048%
3	27	0.010%
4	76	0.281%
5	199	0.735%

The 2 age groups differed in the reported causes of their fevers as shown in Sponsor's Table 6. Although upper respiratory tract infection was the most commonly reported cause of fever for both age groups, in children < 2 years of age, otitis media was more common (p<0.001) as compared to children ≥ 2 years of age, who were more commonly afflicted with pharyngitis and lower respiratory tract infections (p<0.001 for

both comparisons).

Sponsor's Table 6 - Cause of Fever Among All Participants

Illness (%)	Age	
	< 2 years (n =27,065)	≥ 2 years (n = 56,850)
Upper Respiratory Infection	43	42
Otitis Media	48[1]	27
Pharyngitis	19	40[2]
Lower Respiratory Infection	6.3	8.8[3]
Gastrointestinal Infection	3.0	3.2

[1]Statistically significant difference at (p<0.001).
[2]Statistically significant difference at (p<0.001).
[3]Statistically significant difference at (p<0.001).

Sponsor's Table 7 shown immediately below demonstrates that there were no differences in the causes of fever in the 27,065 participants ≤ 2 years of age when examined by randomized antipyretic treatment group.

Sponsor's Table 7 - Cause of Fever Among 27,065 Participants ≤ 2 Years Old According to Treatment Group

Illness (%)	Acetaminophen	Ibuprofen (5 mg/kg)	Ibuprofen (10 mg/kg)
Upper Respiratory Infection	43	43	43
Otitis Media	48	48	48
Pharyngitis	20	19	20
Lower Respiratory Infection	6.5	6.2	6.2
Gastrointestinal Infection	3.0	3.3	2.8

The following 2 tables, Sponsor's Tables 8 and 9, show by age and randomized treatment group the numbers and percentages of children who were randomized, but did not receive study medications. The tables also show that the median number of doses and the median duration of treatment by those who did receive medications was very similar for the subcohort and the original cohort groups, as well as all 3 treatment groups ≤ 2 years of age.

Sponsor's Table 8 - Study Medication Use Among All Participants

Exposure	Age < 2 years (n =27,065)	Age ≥ 2 years (n = 56,850)
Treated, %	96.1	95.1
Not Treated, %	3.9	4.9
Doses Received (Median)	6-10	6-10
Duration in Days (Median)	3	3

Sponsor's Table 9 - Study Medication Use Among 27,065 Participants ≤ 2 Years Old According to Treatment Group

Exposure	Acetaminophen	Ibuprofen (5 mg/kg)	Ibuprofen (10 mg/kg)
Treated, %	96.1	96.1	96.0
Not Treated, %	3.9	3.9	4.0
Doses Received (Median)	6-10	6-10	6-10
Duration in Days (Median)	3	3	3
Dose (mg/kg) (Median)	12	4.8	9.6

Study Outcomes:

Although no deaths were reported to have occurred during the duration of the study, 2 children did die during the follow-up period. Both deaths were unrelated to the study medications. The first case involved a 15-month-old black male randomized to the acetaminophen treatment group who died as a result of injuries sustained in a motor vehicle accident. The second case involved an 11-year-old male randomized to ibuprofen 5 mg/kg who died due to complications of meningitis.

The original objective of this study was to assess the risk associated with the use of ibuprofen in febrile children for the occurrence of serious adverse events. The objective of the subcohort analysis was to describe the risk of serious adverse clinical events following the use of ibuprofen in a study subcohort of children < 2 years of age. The original analysis of the entire study cohort found that only 795 (1%) participants out of the 83,915 randomized to receive study medications were hospitalized for any reason during the 4 weeks following study entry. In the subcohort analysis, 385 out of the 27,065 children < 2 years of age and 410 out of 56,850 children ≥ 2 years of age were hospitalized for any reason. (See Sponsor's Table 10, below.)

As part of the statistical analysis of this new subcohort examination, absolute risk and relative risk for the development of serious outcomes were designated to be calculated for comparison purposes by both age and treatment groups for "any" as well as for specifically predesignated adverse events that are of a safety concern in pediatric populations exposed to ibuprofen (i.e., GI bleeding, acute renal failure, anaphylaxis, or Reye Syndrome.) In the < 2 years of age subcohort, the absolute risk for hospitalization due to any reason was found to be 1.4% (95% confidence interval, 1.3-1.6%) vs 0.72% (95% CI, 0.65-0.79%) for children ≥ 2 years of age. (Refer to Sponsor's Table 10 below.) The relative risk for hospitalization due to any reason in the < 2 years of age subcohort as compared to the subcohort ≥ 2 years was found to be 2.0 (95% CI, 1.7-2.3). (See Sponsor's Table 10.)

Sponsor's Table 10 - Risk of Hospitalization for Any Reason According to Age

Age	Total Number	No.Hospitalized	Absolute Risk (95% CI[1])	Relative Risk[2] (95% CI)
<2 yrs.	27,065	385	1.4% (1.3-1.6%)	2.0 (1.7-2.3)
≥2 yrs.	56,850	410	0.72% (0.65-0.79%)	1.0 (--)

[1]Confidence interval.
[2]Risk of hospitalization among children < 2 years of age compared to the risk of hospitalization among children ≥ 2 years of age.
[3]Reference category.

Only 2 out of the 319 infants < 6 months of age who were included in the study were hospitalized. The first case involved an infant hospitalized for the treatment of a viral infection who had been assigned to ibuprofen 5 mg/kg. The other case involved an infant hospitalized with pneumonia who had been assigned to the ibuprofen 10 mg/kg treatment group.

As part of the new "sub" subcohort analysis, the absolute risk of hospitalization for any reason for the 319 infants < 6 months old regardless of antipyretic treatment was 0.63% (95% CI, 0.08-2.2%). When compared to the risk of hospitalization in children ≥ 6 months of age, no significant difference was shown (p=0.8) between these 2 age groups. No significant difference (p=0.5) was also found when comparing the risk of hospitalization for any reason according to assigned antipyretic treatment in infants < 6 months of age.

The following table, Sponsor's Table 11, shows that when comparing the risk for hospitalization for any reason by treatment group assignment according to age, children < 2 years of age treated with ibuprofen (relative risk: 2.1 [95% CI, 1.8-2.5]) and acetaminophen (relative risk - 1.7 [95% CI, 1.8-2.5]) were at a significantly higher risk than children ≥ 2 years old (ibuprofen - relative risk: 1.0 [95% CI]; acetaminophen - relative risk - 1.0 [95%, CI]). (Refer to Sponsor's Table 11 below.) No increase in the risk for hospitalization was noted on comparison of within age groups according to treatment as shown in the next table, Sponsor's Table 12, as shown below. (See the

following table, Sponsor's Table 12.)

Sponsor's Table 11 - Risk of Hospitalization for Any Reason According to Antipyretic Assignment and Age

Antipyretic	Age	Total Number	No. Hospitalized	Absolute Risk/100,000 (95% CI[1])	Rel. Risk[2] 95% CI
Ibuprofen	<2 yrs.	17,938	261	1.5% (1.3-1.6%)	2.1 (1.8-2.5)
	≥2yrs.	37,847	262	0.69% (0.61-0.78%)	1.0[3] (—)
Acetaminophen	<2 yrs.	9,127	124	1.4% (1.1-1.6%)	1.7 (1.4-2.2)
	≥2yrs.	19,003	148	0.78 (0.66-0.91%)	1.0[3] (—)

[1]Confidence Interval.
[2]Risk of hospitalization among children < 2 years of age compared to the risk of hospitalization with among children randomized to ≥ 2 years of age.
[3]Reference category.

Sponsor's Table 12 - Risk of Hospitalization for Any Reason According to Age and Antipyretic Assignment

Age	Antipyretic	Total Number	Number Hospitalized	Absolute Risk (95% CI[1])	Relative Risk[2] (95% CI)
<2 years	Ibuprofen	17,938	261	1.5% (1.3-1.6%)	1.1 (0.87-1.3)
	Acetaminophen	9,127	124	1.4% (1.1-1.6%)	1.0[3] (—)
≥2 years	Ibuprofen	37,847	262	0.69% (0.61-0.78%)	0.89 (0.73-1.1)
	Acetaminophen	19,003	148	0.78 (0.66-0.91%)	1.0[3] (---)

[1]Confidence Interval.
[2]Risk of hospitalization among children randomized to ibuprofen compared to the risk of hospitalization among children randomized to acetaminophen.
[3]Reference category.

As stated earlier, one of the original aims of the Boston Fever Study was to assess the risk for the occurrence of GI bleeding, acute renal failure, anaphylaxis and Reye Syndrome in the pediatric population studied. In the original cohort of 83,915 patients that were entered into the study, there were only 4 reported cases of GI bleeding, and no cases of acute renal failure, anaphylaxis, or Reye Syndrome. Sponsor's Table 13 (see below) shows the distribution and the absolute risk by age group for a hospitalization due to acute GI bleeding in the subcohort analysis. In children < 2 years of age, this risk was found to be 11 per 100,000 (95% CI, 2.2 to 32 per 100,000). Since these numbers were so low, there was insufficient data to show a significant difference (Fisher's exact test, p=0.1) when compared with the risk for acute GI bleeding in children ≥ 2 years of age.

Sponsor's Table 13 - Risk of Hospitalization With Acute Gastrointestinal (GI) Bleeding According to Age

Age	Total Number	No.Hospitalized	Absolute Risk per 100,000	95% CI[1]
<2 years	27,065	3	11	2.2-32
≥2 years	56,850	1	1.8	0.05-9.8

[1]Confidence Interval

As seen in Sponsor's Table 14 (below), all of the GI bleeds occurred in children treated with ibuprofen. Although the highest absolute risk of hospitalization due to an acute GI bleed was found to be associated with children < 2 years of age treated with ibuprofen (17 per 100,000 [95% CI, 3.5-49 per 100,000]), the sponsor reported the risk for the two ibuprofen treatment groups within that age group was similar. However, it was not found to be significantly increased (p=0.6) when compared to the risk associated with children < 2 years of age who were treated with acetaminophen (0 per 9,127 [95% CI, 0-33 per 100,000]). (Refer to Sponsor's Table 14.) In children ≥ 2 years of age, the risk of a hospitalization due to acute GI bleeding in the ibuprofen treated group was 2.6 per 100,000 (95% CI, 0.05-15 per 100,000), and in the acetaminophen treated group it was 0 per 19,003 (95% CI, 0-16 per 100,000). On comparison of the 2 age groups, the risk for hospitalization due to an ibuprofen-induced acute GI bleed was not found to be significantly different (p=0.1).

APPEARS THIS WAY ON ORIGINAL

Sponsor's Table 14 - Risk of Hospitalization with Acute GI bleeding According to Age and Antipyretic

Age	Antipyretic	Total Number	Number Hospitalized	Absolute Risk per 100,000	95% CI
<2 yrs.	Ibuprofen<br>Acetaminophen	17,938<br>9,127	3<br>0	17<br>--	3.5-49<br>0-33
≥2 yrs.	Ibuprofen<br>Acetaminophen	37,847<br>19,003	1<br>0	2.6<br>--	0.05-15<br>0-16

None of the 3 cases of acute GI bleed that occurred in the subcohort study population of < 2 years of age died. The first case (Subject ID 78468989) occurred in a 19-month-old male with a history of Hirschsprung's disease, status post colostomy and Swenson pull-through, and enterocolitis who was randomized to the ibuprofen 50 mg/5 mL treatment group when he presented with a fever due to otitis media. In addition, he also received a course of an unknown antibiotic. This subject received 3 doses of ibuprofen over the next 2-days. On the third day he was hospitalized for evaluation of abdominal pain and vomiting. Records state that his vomitus appeared to look like coffee grounds, and his stool was guaiac positive. He was treated with enemas and stool softeners for a possible bowel obstruction, and improved without further recurrence of GI bleeding during the 9 months of post-study follow up.

The second case (Subject ID 43135762) of acute GI bleed occurred in a 19-month-old male randomized to the ibuprofen 100 mg/5 mL treatment group who hospitalized the day after receiving just 1 dose of the study medication due to guaiac positive diarrhea associated with persistent vomiting. His stool assay was positive for rotavirus antigen. He improved after treatment with IV fluids, antibiotics, and acetaminophen without further episodes of bleeding during the 20 months of post-study follow up.

The last case (Subject ID 85496241) of acute GI bleeding occurred in a 8-month-old female randomized to the ibuprofen 100 mg/5 mL treatment group who had a fever due to a persistent case of otitis media which was treated with Augmentin. She was admitted on the third study day, 48 hours after receiving 2 doses of the study medication over a 24-hour period for evaluation of hematochezia and guaiac positive stools associated with dehydration, vomiting and otitis media. The subject improved with IV hydration and antibiotics and the treating physician attributed the hematochezia to the study medication. There were no reports of the hematochezia recurring during the 2 week post-study follow up.

Although there were no reported cases of acute renal failure, anaphylaxis or Reye syndrome which occurred during this study, the sponsor did calculate the observed risk for both the original study cohort population as well as that of the new subcohort analysis. Since there were no reported cases of these 3 specific adverse

events during the study, only the upper-bound of the 95% confidence interval (CI)could be calculated. In children < 2 years of age, the upper bound of the 95% CI for the risk of hospitalization due to acute renal failure, anaphylaxis, or Reye Syndrome was found regardless of the treatment group was 11 per 100,000; in children ≥ 2 years of age the upper bound for these events was 5.1 per 100,000. (Refer to Sponsor's Table 13.) In children < 2 years of age, the upper bound of the 95% CI for the risk of hospitalization due to these events treated with acetaminophen was found to be 0 per 9,127(95% CI, 0-33 per 100,000); in children < 2 years of age treated with ibuprofen the upper bound for these events was 0 per 17,938 (95% CI, 0-17 per 100,000). (Refer to Sponsor's Table 14.) In infants < 6 months of age, the observed risk of hospitalization for each of the above specific adverse events regardless treatment was 0 per 319 (95% CI, 0-0.94); among infants who received treatment with acetaminophen the observed risk was 0 per 112 (95% CI, 0 to 2.7%); among infants who received treatment with ibuprofen the observed risk was 0 per 207 (95% CI, 0 to 1.5%). (Note: The differences noted in the upper bound of the 95% CI for the infant population is due to its small sample size.)

In view of the fact that there were no cases of acute renal failure which occurred during this trial, the sponsor decided to look at changes in subjects' serum creatinine levels as another means of possibly determining the nephrotoxicity of ibuprofen in the pediatric population. Since the original protocol did not require the measurement and collection of entry and exit serum creatinines, they did a post hoc analysis from lab data collected from 222 (28%) out of the 795 children who were hospitalized while participating in the study. (Note: Only serum creatinines obtained within the first 24-hours of admission were used in this analysis.) The mean creatinine level on admission was 0.48 mg/dL, and 9% of them were higher than 0.7 mg/dL which is the upper limit of normal for children. No significant difference in mean serum creatinine levels was noted when compared by treatment group. Only 112 (29%) out of the 385 children < 2 years of age who were admitted during this study had serum creatinine levels available for analysis. The following table, Sponsor's Table 15 shown below, lists the distribution, mean and range for the serum creatinines collected for data analysis in this age group. (See Sponsor's Table 15.) On cross-treatment group comparison, the difference in mean serum creatinine between the acetaminophen group (0.34 mg/dL) and the ibuprofen treatment group (0.42 mg/dL) was found to be statistically significant (p=0.03) via calculation of an unpaired student's t-test, but when analysis of covariance is used to calculate the p-value taking into account subjects' ages, weight, sex and dehydration, no significant difference was found. Comparison of the prevalence of serum creatinines > 0.07 mg/dL in the acetaminophen and ibuprofen treatment groups, was not found to be significantly different (p=0.32). (See Sponsor's Table 15 below.) (Note: The sponsor reports that although they repeated this analysis with lower thresholds set for an "elevated" serum creatinine, the numbers of cases increased in both treatment groups but the difference was still not statistically significant. Although this data was not included in the submission for review, it needs to be mentioned to document the scope of the sponsor's post hoc analysis.)

Sponsor's Table 15 - Serum Creatinine Among Hospitalized Children < 2 Years Old

	Acetaminophen	Ibuprofen
**Total Number**	29	83
**Serum Creatinine (mg/dL)**<br>**Mean**<br>**(SEM)**<br>**Range**	 0.34<br>(0.025)<br>0.1-0.7	 0.42<br>(0.023)<br>0.1-1.4
**Serum Creatinine >0.7 mg/dL**<br>**Number**<br>**(%)**	 0<br>(0)	 5<br>(6)

The following table, Sponsor's Table 16, lists the mean serum creatinines by treatment group for the subcohort of children < 2 years of age. The sponsor states that they did not do a subanalysis of mean serum creatinines in the subgroup infant population < 6 months of age because too few of these subjects were hospitalized.

Sponsor's Table 16 - Mean Serum Creatinine Among Hospitalized Children < 2 Years Old By Age and Treatment Group

Age	Mean Creatinine	(No.)	Mean Creatinine	(No.)	Mean Creatinine	(No.)
All	0.34	(29)	0.43	(46)	0.40	(73)
12-23 mos.	0.37	(17)	0.44	(25)	0.43	(21)
<12 mos.	0.32	(12)	0.43	(21)	0.36	(16)

The sponsor also looked at the risk for hospitalizations associated with other adverse events or conditions that may be of potential risk in this younger pediatric age group. They looked at asthma, bronchiolitis, and vomiting/gastritis since these occurred in at least 5 or more subjects in the subcohort population. Sponsor's Table 17, below, shows that there were 32 children < 2 years of age and 36 children ≥ 2 years of age who were hospitalized due to asthma while participating in the trial. The relative risk for hospitalization with asthma in children < 2 years of age was found to be 1.9 (95% CI 1.2 to 3.0) when compared to that in children ≥ 2 years of age.

Sponsor's Table 17 - Risk of Hospitalization with Asthma According to Age

Age	Total Number	No.Hospitalized	Absolute Risk/100,000 (95%CI)	Relative Risk[2] (95% CI)
<2 yrs.	27,065	32	120 (81-70)	1.9 (1.2-3.0)
≥2 yrs.	56,850	410	63 (44-88)	1.0[3] (–)

[1]Confidence interval.
[2]Risk of hospitalization with asthma among children < 2 years of age compared to the risk of hospitalization with asthma among children ≥ 2 years of age.
[3]Reference category.

The following table, Sponsor's Table 18 below, lists in a table the associated absolute and relative risks for the 2 age groups by treatment for hospitalization with asthma. This table shows that regardless of the antipyretic treatment, the risk of hospitalization is inversely related to the child's age.

Sponsor's Table 18 - Risk of Hospitalization with Asthma According to Antipyretic Assignment and Age

Age	Antipyretic	Total Number	Number Hospitalized	Absolute Risk/100,000 (95% CI[1])	Relative Risk[2] 95% CI
<2 yrs.	Ibuprofen	17,938	20	110 (68-170)	1.8 (1.0-3.2)
	Acetaminophen	9,127	24	63 (41-94)	1.0[3] (—)
≥2 yrs.	Ibuprofen	37,847	12	130 (70-230)	2.0 (0.9-4.6)
	Acetaminophen	19,003	12	63 (33-110)	1.0[3] (—)

[1]Confidence Interval.
[2]Risk of hospitalization with asthma among children randomized to ibuprofen compared to the risk of hospitalization with asthma among children randomized to acetaminophen.
[3]Reference category.

Sponsor's Table 19 below, shows the distribution of children hospitalized by age and treatment group for the risk of hospitalization due to asthma. The data in this table demonstrates that treatment with either antipyretic agent was not associated with the risk of hospitalization in either age group. (Refer to Sponsor's Table 19 shown below.)

Sponsor's Table 19 - Risk of Hospitalization with Asthma According to Age and Antipyretic Assignment.

Age	Antipyretic	Total Number	Number Hospitalized	Absolute Risk/100,000 (95% CI[1])	Relative Risk[2] 95% CI
<2 years	Ibuprofen	17,938	20	110 (68-170)	0.9 (0.4-1.7)
	Acetaminophen	9,127	12	130 (70-230)	1.0[3] (—)
≥2 years	Ibuprofen	37,847	24	63 (41-94)	1.0 (0.5-2.0)
	Acetaminophen	19,003	12	63 (33-110)	1.0[3] (—)

[1]Confidence Interval.
[2]Risk of hospitalization with asthma among children randomized to ibuprofen compared to the risk of hospitalization with asthma among children randomized to acetaminophen.
[3]Reference category.

Since it can be difficult to discern between asthma and bronchiolitis in very young children, the sponsor looked at the 37 hospitalized cases of bronchiolitis which occurred during the study. The following 2 tables, Sponsor's Tables 20 and 21, show the study data describing the risk associated with hospitalizations due to bronchiolitis in both subcohort age groups by age as well as treatment group.

Sponsor's Table 20 - Risk of Hospitalization With Bronchiolitis According to Age

Age	Total Number	No.Hospitalized	Absolute Risk per 100,000	95% CI[1]
<2 years	27,065	33	120	84-170
≥2 years	56,850	4	7	2-18

[1]Confidence Interval

Sponsor's Table 21, below, shows that on comparison of the 2 treatment groups, the risk for hospitalization due to bronchiolitis did not vary.

Sponsor's Table 21 - Risk of Hospitalization with Bronchiolitis Among Participants <2 Years of Age According to Antipyretic Assignment

Antipyretic	Total Number	Number Hospitalized	Absolute Risk/100,000 (95% CI[1])	Relative Risk[2] (95% CI)
Ibuprofen	17,938<br>9,127	21	120<br>(72-180)	0.9<br>(0.4-1.8)
Acetaminophen	9,127	21	130<br>(70-230)	1.0[3]<br>(—)

[1]Confidence Interval.
[2]Risk of hospitalization with bronchiolitis among children randomized to ibuprofen compared to the risk of hospitalization with bronchiolitis among children randomized to acetaminophen.
[3]Reference category.

The sponsor also looked at the number of cases who were hospitalized due to vomiting/gastritis during the study. Sponsor's Table 22, below, shows the numbers of children and the associated risks for hospitalization due to vomiting/gastritis for both subcohort populations. On comparison between age groups, the risk for hospitalization due to vomiting/gastritis did not vary.

Sponsor's Table 22 - Risk of Hospitalization With Vomiting/Gastritis According to Age

Age	Total Number	No.Hospitalized	Absolute Risk/100,000 (95% CI[1])	Relative Risk[2] (95% CI)
<2 years	27,065	9	33<br>(15-63)	1.1<br>(0.5-2.5)
≥2 years	56,850	17	30<br>(17-48)	1.0[3]<br>(—)

[1]Confidence Interval.
[2]Risk of hospitalization with vomiting/gastritis among children randomized to ibuprofen compared to the risk of hospitalization with vomiting/gastritis among children randomized to acetaminophen.
[3]Reference category.

The last table, Sponsor's Table 23, below, demonstrates that the risk for hospitalization due to vomiting/gastritis did not increase with treatment with either acetaminophen or ibuprofen, nor was it shown to vary with age or antipyretic treatment.

Sponsor's Table 23 - Risk of Hospitalization with Vomiting/Gastritis According to Antipyretic Assignment and Age

Antipyretic	Age	Total Number	No. Hospitalized	Absolute Risk/100,000 (95% CI[1])	Rel. Risk 95% CI
Ibuprofen	<2 yrs.	17,938	7	39 (16-80)	1.1 (0.5-2.9)
	≥2yrs.	37,847	13	34 (18-59)	1.0[3] (—)
Acetaminophen	<2 yrs.	9,127	2	22 (2.6-79)	NA[4]
	≥2yrs.	19,003	4	21 (5.8-54)	1.0[3] (—)

[1]Confidence Interval.
[2]Risk of hospitalization with vomiting/gastritis among children < 2 years of age compared to the risk of hospitalization with vomiting/gastritis among children randomized to ≥ 2 years of age.
[3]Reference category.
[4]Relative risk not calculated because the number hospitalized in at least one group was < 5.

Medical Reviewer's Comments: There are many methodological problems associated with this subcohort analysis of the Boston Fever Study. The original study was unable to accomplish one of its aims which was to assess the risk associated with the use of ibuprofen in a pediatric population for developing GI bleeds, acute renal failure, anaphylaxis and Reye syndrome. It is unclear if this was due to problems failing to measure or capture these adverse events or if the design introduced selection bias based on having health care providers "select" good candidates (i.e., children who were not too sick and had intelligent caretakers.) Since the new subcohort analysis was a post hoc analysis of the original trial data, the validity of its findings are subject to the same issue.

Some of the laboratory data subanalyses performed in this submission did not make good sense to this reviewer such as using the serum creatinines as surrogate markers for more significant problems were not validated.

The original protocol also had an age entry criteria of > 6 months, but the subanalysis reveals that 319 infants ≤ 5 months old were entered into the study. These enrollments constitute trial violations and thus, both the subcohort and "sub-subcohort" infant analysis which draw on this data for support technically should be discounted.

Despite these methodological flaws, the study's size does provide some useful information. Thus, based on the above study data reviewed, and the paucity of adverse events that actually occurred in such a large pediatric population (subcohort population

of n=27,065), it is fairly obvious that ibuprofen at the 2 doses tested is safe to be used in an OTC pediatric population < 2 years of age. The real question posed to this reviewer is at what age is it no longer safe to be used as an OTC product? Unfortunately, there is no answer to that question based on the data submitted in this SNDA. Sponsor's Table 5, demonstrates numerically how few infants between the ages of 2 and 5 months actually participated (as protocol violations no less) in the study (n=319), with the percentage of infants < 6 months of age enrolled in the study comes to only < 1.2% of the total subcohort population. Thus, it is the opinion of this medical reviewer that this study fails to generate sufficient support for a pediatric OTC claim in children < 6 months of age.

2. McNeil CPC controlled clinical trial data on subjects ≤ 2-years of age enrolled on or after November 17, 1993 and treated with ibuprofen.

Since the above listed date, sponsor states in this submission that they have not conducted any clinical trials in children ≤ 2 years of age. One 19-month-old child was inadvertently randomized to the ibuprofen suspension 7.5 mg/kg treatment group of a 2-arm, single-dose, randomized, investigator-blinded antipyresis trial that compared ibuprofen to acetaminophen 12.5 mg/kg. The child reportedly did not experience any adverse effects from this exposure.

Medical Reviewer's Comments: Noted.

3. McNeil CPC Spontaneous Reporting System for McNeil CPC ibuprofen products in children ≤ 2-years of age for the time period November 17, 1993 through October 2, 1997, including serious reports in the published literature.

A search of the sponsor's own CPC Spontaneous Reporting System (SRS) for both serious and nonserious adverse event reports in children ≤ 2 years of age who ingested either the prescription or OTC formulations of Children's Motrin® yielded 9 serious and 305 nonserious reports from health care professionals and consumers. A total of 18 and 361 adverse events were generated by COSTART terminology respectively for serious and nonserious adverse events. Two (2) out of the 9 serious cases resulted in the deaths of the children due to Invasive Group A streptococcal infection post-varicella infection (1) and renal failure (1). The 7 remaining serious cases resulted in the hospitalizations of the children involved due to the following adverse events: drug-induced anaphylaxis (1), dehydration (1), anemia (1), and sepsis syndrome secondary to varicella lesions (4). The sponsor has provided the following summary table, Table 8-40, which describes and lists these 9 serious cases in tabular format in children ≤ 2 years of age. Table 8-41, lists all of the 361 nonserious adverse event reports by body system. The sponsor reported in this submission that out of the original 305 nonserious reports received by them in this age group, 272 reports were associated with their (OTC) Children's Motrin® Suspension formulation, 14 reports were

Table 8-40. AE Reports with Serious Outcomes in Children Less Than Two Years of Age Received by McNeil CPC from November 17, 1993 through October 2, 1997 for Motrin® Ibuprofen Products, Children's Motrin® Ibuprofen Products, and Unknown Pediatric Ibuprofen Products

Case No.	Product Form[1]	Date Received	Age	Sex	AE	COSTART Term	Daily Dose	Duration of Drug	Outcome
1	MOS	04/06/95	8 mo	Female	Cellulitis<br>Anemia	Cellulitis,<br>Anemia	Unknown	Unknown	Hospitalization
2	CMS	01/27/97	11 mo	Male	Lips and eyes swelled<br>Increase in number of hives<br>Trouble breathing	Edema face<br>Urticaria<br>Dyspnea	Unknown	1 dose	Hospitalization
3	MOS	09/23/97	11 mo	Male	Renal failure	Kidney failure	10 mg/kg per dose	Unknown	Hospitalization[2]
4	MOS	10/26/94	1 yr	Male	Creatine phosphokinase increased<br>Convulsion<br>Septic shock<br>Gastrointestinal hemorrhage	Creatine phosphokinase increased<br>Convulsion<br>Sepsis<br>Gastrointestinal hemorrhage	100 mg, q4h	6-8 months	Hospitalization
5	MOS	02/23/95	1 yr	Female	Dehydration	Dehydration	Unknown	Unknown	Hospitalization
6	MOS	02/23/95	1 yr	Male	Cellulitis face<br>Bilateral otitis media	Cellulitis<br>Otitis media	Unknown	Unknown	Hospitalization
7	MOS	02/23/95	1 yr	Female	Infection	Infection	Unknown	Unknown	Hospitalization
8	CMS	02/09/96	15 mo	Male	Hemoglobin and hematocrit decreased	Hypochromic anemia	10mg/kg, q8h	8 days	Hospitalization
9	MOS	02/23/95	1.5 yr	Male	Sepsis<br>Meningitis<br>Cardiac arrest	Sepsis<br>Meningitis<br>Heart arrest	Unknown	Unknown	Death

[1] MOS = Prescription Motrin® ibuprofen suspension, CMS = OTC Children's Motrin® ibuprofen suspension.
[2] The infant's renal function recovered. The physician reported that the infant died some time later due to unknown complications unrelated to the reported event.

Table 8-41. Body System Summary for AE Reports with Nonserious Outcomes For Children Less Than Two Years of Age Received by McNeil CPC from November 17, 1993 through October 2, 1997 for Motrin® Ibuprofen Products, Children's Motrin® Ibuprofen Products, and Unknown Pediatric Ibuprofen Products

Body System<br>Adverse Event	Number
**Body as a whole**	87
Asthenia	2
Edema face	8
Hypothermia	3
Lab test abnormal	2
Malaise	2
No drug effect	21
Overdose	1
Accidental Overdose	40
Pain	1
Abdominal pain	7
**Cardiovascular system**	2
Tachycardia	1
Peripheral vascular disease	1
**Digestive system**	62
Anorexia	1
Constipation	4
Diarrhea	14
Dyspepsia	3
Dysphagia	2
Eructation	1
Flatulence	3
Hemorrhagic gastritis	1
Glossitis	2
Gastrointestinal hemorrhage	1
Nausea	1
Stomatitis ulcer	1
Abnormal stools	5
Vomiting	23
**Hemic and lymphatic system**	2
Ecchymosis	1
Eosinophilia	1
**Metabolic and nutritive disorder**	2
Peripheral edema	1
Hyperglycemia	1
**Musculoskeletal system**	1
Arthralgia	1
**Nervous system**	119
Confusion	1
Convulsion	1
Dizziness	2
Abnormal dreams	1
Emotional lability	2
Abnormal gait	1
Hallucinations	1
Hyperkinesia	13
Insomnia	30
Nervousness	36
Restlessness	12
Screaming syndrome	6
Somnolence	10
Stupor	1
Tremor	2

Table 8-41. Body System Summary for AE Reports with Nonserious Outcomes For Children Less Than Two Years of Age Received by McNeil CPC from November 17, 1993 through October 2, 1997 for Motrin® Ibuprofen Products, Children's Motrin® Ibuprofen Products, and Unknown Pediatric Ibuprofen Products

Body System<br>Adverse Event	Number
**Respiratory system**	10
Burning of the throat	1
Cough increased	1
Dyspnea	3
Epistaxis	3
Pharyngitis	1
Rhinitis	1
**Skin and appendages**	71
Erythema multiforme	1
Pruritus	4
Rash	46
Skin discolor	1
Sweat	3
Urticaria	16
**Urogenital system**	5
Oliguria	1
Urine abnormality	4
**Total for all body systems**	361

APPEARS THIS WAY ON ORIGINAL

for their Children's Motrin® Drops, 1 report was for their Children's Motrin® Chewable tablets, 17 were for their prescription Motrin® Suspension, and 1 was for their prescription Motrin® Drops.

Medical Reviewer's Comments: Review of the narratives of these 9 serious adverse event cases does not reveal any information that could signal any unforeseen adverse event associated with the use of OTC ibuprofen in children ≤ 2 years of age. Cases associated with Invasive Group A Streptococcal infections have been reviewed by the agency's epidemiologists in the past, and no association was found.

4. FDA Spontaneous Reporting System (SRS) for all ibuprofen products in children ≤ 2-years of age for the time period November 1, 1993 through August 25, 1997. (Note: Adverse events reported through McNeil Spontaneous Reporting System are not included here.)

A query of the FDA's SRS database yielded 20 serious adverse event reports in children < 2 years of age which were related to either the use of a prescription or OTC formulation of ibuprofen. Two out of the 20 cases were reports which describe the same fatal overdose case in a 23-month-old female who died due to aspiration pneumonia that were submitted by the sponsor's competitor. (Note: This was a case of an accidental overdose, a further description of which can be found in the following 6b. Overdose Section below.) The sponsor has prepared the following 2 tables, Tables 8-42 and 8-43, which list by COSTART body system terminology all 51 of the adverse events coded for these 19 serious cases (Table 8-42), and a tabular summary of the 19 cases themselves (Table 8-43). Four (4) out of these 19 serious cases resulted in the death of the child due to pulmonary hemorrhage (1), sepsis with cardiac arrest (2), and aspiration pneumonia (1). (Note: The last case is the case that was reported twice to the system.)

The next table, Table 8-44, lists the 145 nonserious adverse events generated from a total of 52 case reports in the FDA's SRS database by COSTART terminology.

Medical Reviewer's Comments: Review of these 19 serious cases does not reveal any information that could signal any unforeseen adverse event associated with the use of OTC ibuprofen in children ≤ 2 years of age. However, one must keep in mind that these cases occurred in situations where access to the drug was controlled by a health care provider (i.e., via a prescription). Thus, this reviewer is unable to predict if the occurrence of these events will increase in frequency when this product is available to a pediatric population ≤ 2 years of age.

5. Published randomized controlled clinical trials and human pharmacokinetic studies of ibuprofen products for the years 1966 through October 1997 that reported including children ≤ 2-years of age.

An extensive literature search of the worldwide literature by the sponsor yielded a total of 29 articles which discussed the data from 21 single-dose and multi-dose clinical studies with a total combined pediatric population of 3,006 subjects. (Note: More information about these studies can be found in the preceding efficacy section, and in the Sponsor's Tables 8-10 and 8-12, in Attachment I.) No serious adverse events were reported to have occurred in any of these studies. Two studies did not report any safety data and thus are excluded from this safety review. Nine out of the remaining 19 trials did report the occurrence of non-serious adverse events in ibuprofen-treated children which included: nausea, vomiting, diarrhea, rash, hypoglycemia, agitation, febrile seizures, exanthem, insomnia, hypothermia, epistaxis, sweating, GI complaints, discomfort, and hypothermia. Many of these adverse events were not considered by the authors of these published studies to be related to treatment with ibuprofen. Since these trials only used descriptive statistics in discussing their patient populations, it is impossible for this reviewer to determine if any of the above listed adverse events occurred in subjects < 2 years of age based on the data presented.

A total of 340 children between the ages of 3 months to 12 years were enrolled in the 5 pharmacokinetic studies submitted in support of this application. The investigators of these studies did not report the occurrence of any serious or non-serious adverse event during these trials. (Refer to the PK review of this NDA review for more information.)

Medical Reviewer's Comments: This reviewer agrees with the authors of these studies that most of the adverse events reported associated with these trials were probably related to the subjects underlying febrile illnesses (febrile seizures, discomfort, exanthem, nausea, vomiting, etc . . .). Although some events such as the GI complaints, epistaxis, and rash could be drug-related and are known to occur with this product they could also be due to the subjects' underlying illnesses. Since the sponsor did not submitted the case forms for these studies, it is impossible for this medical reviewer to draw any conclusions regarding ibuprofen's safety profile in the pediatric populations that participated in these studies.

6. Overdose Data: (a.) AAPCC TESS ibuprofen data from the years 1994 through 1996 for children ≤ 2-years of age. (The 1997 report was not yet available.) (b.) Reports from the FDA's Spontaneous Reporting System. (c.)Reports from McNeil's CPC Drug Safety Reporting System.

The American Association of Poison Control Centers (AAPCC) Toxic Exposure Surveillance System (TESS) collected a total of 2,726,446 reports of possible human poisonings due to therapeutic drugs during the time period of 1994-1996. The sponsor has provided in this submission the data pertaining to ibuprofen overdoses. A total of

118,841 reports (4.4%) out of all of the reports collected for this time period were due to an ibuprofen containing product. In children < 2 years of age, there was a total of 17,635 reports of exposures to ibuprofen for this time period, out of which 17,173 (97.4%) were classified as non-toxic, minor, minimal or no effect reported. Of the remaining 462 case reports, 433 (2.3%) reported an unrelated effect or were lost to follow up. Although a total of 29 cases in this age group were classified as having resulted in a moderate (25 cases) or major (4 cases) outcome, none resulted in a death of a child. Only 24 out of these 29 cases with a moderate or major outcome involved either unknown pediatric formulations or an adult formulation of ibuprofen. Table 8-46, at the end of this section prepared by the sponsor lists these cases by increasing chronological age.

In addition, the sponsor obtained data from the FDA's Spontaneous Reporting System (SRS) for the time period November 1,1993 through August 25, 1997 and also queried its own data base for any case reports of ibuprofen overdoses in children < 2 years of age. This search of the SRS database yielded 22 reports, out of which 4 were listed as having serious outcomes. The following attached sponsor's table, Table 8-47, lists these 4 cases. Two of the 4 cases (MR 970170176 and MR 897009001S) which resulted in the death of a 23-month-old female child appear to be the same case. Review of the associated case reports reveals that this case was confounded by some underlying unspecified enzyme deficiencies as well as other congenital abnormalities in the child. The child reportedly suffocated on her vomitus while in bed after receiving an overdose of a competitor's ibuprofen suspension for the treatment of a fever. The other 2 cases involved a 17-month-old male who accidently ingested 27-28 tablets of an OTC adult formulation of ibuprofen. He was hospitalized for observation following emergency treatment for the drug overdose and survived without any reported sequelae. The last case was a report from worldwide literature about a 21-month-old male with a history of hypocalcemia and hypomagnesemia who was hospitalized for the treatment of a metabolic acidosis associated with drowsiness and tachypnea after an overdose of 8 grams of ibuprofen. He subsequently developed acute tonic-clonic seizures and renal failure, but reportedly recovered.

Medical Reviewer's Comments: Since little information is provided regarding whether the 24 cases of non-serious overdoses involved pediatric or unknown adult formulations of ibuprofen, this reviewer at best recommends that the indicated labeled age ranges for the pediatric formulations be modified to improve clarity. As such, it may be prudent to not have overlapping age ranges as one such attempt at minimizing dosing misadventures.

**APPEARS THIS WAY
ON ORIGINAL**

The sponsor submitted reanalyses of data from clinical studies previously conducted. No new clinical trials were conducted in support of this efficacy supplement. Therefore, no debarment certification is required.

APPEARS THIS WAY
ON ORIGINAL

APPEARS THIS WAY
ON ORIGINAL

HFD-560 ActingDir/Bowen
HFD-560 Dep Dir/Katz
HFD-560 Team Leader/Lumpkins
HFD-550 Team Leader/Hyde
HFD-560 MO/Neuner
HFD-560 PM/KRothschild

APPEARS THIS WAY
ON ORIGINAL

Medical Reviewer's Overall Safety Comments: The sponsor has submitted an application in support of their request to lower the current approved age range from 2 to 3 years of age down to 2 months of age for their formulation of pediatric ibuprofen suspension. This product is currently available as a prescription drug for use in children 6 months to 2 years of age who are under a health care provider's care. Thus, the provider has made the determination as to the appropriateness of use of this product in this age group. This controlled access may account for the low incidence of reported post-marketing adverse events associated with ibuprofen suspension in children < 2 years of age. As noted above, most of the overdose safety data in the pediatric population was generated by inadvertent overdosing or accidental ingestion of adult ibuprofen products. At the September 18,1998 NDAC some of the committee members recommended that the age threshold for use of this product might be lowered down to 2 months based on the presentations of data at that meeting, but they also felt that additional warnings needed to appear on the label to safeguard against the use of the product in select populations where additional medical input was needed (i.e., preemies, children with significant fevers, fevers accompanied by lethargy, etc . . .) In face of the fact that the largest supporting source of safety data in a pediatric population < 6 months of age is heavily flawed, and the validity of some of its conclusions are questionable at best, this reviewer feels that there is insufficient safety data in the infant population < 6 months of age to support a lowering of the approved indicated age range to this level.

Recommendations: Based on the data contained in this submission Children's MOTRIN® (ibuprofen oral suspension) Drops, 50 mg/1.25 mL is safe to be used in an OTC pediatric population ≥ 6 months of age. There is insufficient data to currently support an age range lower than the above. Due to the possible threat of dosing misadventures due to consumer confusion, an overlap in dosing age ranges should be avoided for this product and its sister product, Children's MOTRIN® (ibuprofen) Suspension, 100 mg/5 mL. Thus, the concentrated drops should be labeled for use in children ≤ 2 years of age, and the less concentrated solution should be labeled for use in children ≥ 2 years of age. To further help prevent these incidents from happening in the future, the sponsor needs to re-label this product as "concentrate" as follows: Children's MOTRIN® (ibuprofen) Concentrated Drops, 50 mg/1.25 mL.

Rosemarie Neuner, MD

Rosemarie Neuner, MD, MPH
Medical Reviewer, HFD-560

Linda M. Katz, MD

Linda M. Katz, MD, MPH 3/29/99
Deputy Dir., HFD-560

CC: NDA 20-603 File
HFD-560 Div. File
HFD-550 Div. File

McNeil

McNeil Consumer Healthcare. 7050 Camp Hill Road. Fort Washington. PA 19034-2299 (215) 273-7000

Debra L. Bowen, MD
Acting Director
Division of OTC Drug Products (HFD-560)
Center for Drug Evaluation and Research
Document Control Room
Food and Drug Administration
9201 Corporate Boulevard, Room S-212
Rockville, MD 20850

APR 15 1999

Re: Infant's Motrin Concentrated Drops
NDA 20-603/S-003
Revised Commitment Letter

Dear Dr. Bowen:

We acknowledge your fax of 4/15/99 (copy attached) regarding S-003/NDA 20-603. As requested, we agree to the following:

- Labeling described in your fax of 4/15/99 will serve as a basis of approval for S-003;
- Submit Final Printed Labeling consistent with the above.

We trust we have adequately responded to your request. Should you have any questions, please call me at (215) 273-7115.

Sincerely,

McNEIL CONSUMER HEALTHCARE

Willie D. Pagsuyuin
Director, Regulatory Affairs

WDP:dtg
Attachment

cc: Kerry Rothschild (HFD-560)
p:\nda\corresp\bowen3.doc

McNeil

McNeil Consumer Healthcare, 7050 Camp Hill Road, Fort Washington, PA 19034-2299 (215) 273-7000

Debra L. Bowen, MD
Acting Director
Division of OTC Drug Products (HFD-560)
Center for Drug Evaluation and Research
Document Control Room
Food and Drug Administration
9201 Corporate Boulevard, Room S-212
Rockville, MD 20850

APR 14 1999

Re: NDA 20-603/S-003
Children's MOTRIN® Oral Drops

Dear Dr. Bowen:

We acknowledge your fax of 4/14/99 (copy attached) regarding S-003/NDA 20-603. As requested, we commit to the following:

1. Labeling outlined in your fax will serve as a basis of approval for S-003/NDA 20-603. with the following revisions:

   Elimination of the word "OTC" from the instructions to not use the product if the child "has ever had an allergic reaction to any OTC pain reliever/fever reducer".

   Addition of the word "is" to the end of the following subheading: "Ask a doctor or pharmacist before use if the child"

   Under the above subheading, elimination of the word "OTC" in the phrase, "taking any other product that contains ibuprofen, or any other OTC pain reliever/fever reducer."

2. Final printed labeling identical to the labeling described herein will be submitted to FDA.

We trust we have adequately responded to your request.

Sincerely,

McNEIL CONSUMER HEALTHCARE

Paula Oliver For
Willie D. Pagsuyuin
Director, Regulatory Affairs

WDP:dtg
Attachment
p:\nda\corresp\bowen4.doc

cc: Kerry Rothschild (HFD-560)

McNeil

McNeil Consumer Healthcare, 7050 Camp Hill Road, Fort Washington, PA 19034-2299 (215) 273-7000

Debra L. Bowen, MD
Acting Director
Division of OTC Drug Products (HFD-560)
Center for Drug Evaluation and Research
Document Control Room
Food and Drug Administration
9201 Corporate Boulevard, Room S-212
Rockville, MD 20850

APR 13 1999

Re: Children's Motrin Drops
NDA 20-603/S-003
Response to FDA Comments

Dear Dr. Bowen:

We refer to your fax of 4/12/99 (copy attached) which outlined FDA proposed changes to our draft labeling for Children's Motrin Drops. We agree to the changes outlined by FDA in your fax of 4/12/99, as modified below (changes in italics):

A. We acknowledge FDA's recommended alternate name, Baby Motrin Concentrated Drops; however, our preference is to market this product with our original proposed name of Infant's Motrin Concentrated Drops.

B. For clarity, we propose the following change under Important section:

From: **Read all product information before using. Keep this box for important information. This product is intended for use in ages 6 months to 23 months of age.**

To: **Read all product information before using. Keep this box for important information. This product is intended for use *in children* ages 6 months to 23 months.**

C. <u>For clarity, we propose the following changes Under Warnings:</u>

From: **Sore throat warning:** severe or persistent sore throat or sore throat accompanied by high fever, headache, nausea, and vomiting may be serious. Consult physician promptly. Do not use more than 2 days or administer to children under 3 years of age unless directed by a physician.

To*: **Sore throat warning:** severe or persistent sore throat or sore throat accompanied by high fever, headache, nausea, and vomiting may be serious. Consult *a doctor* promptly. Do not use more than 2 days or administer to children under 3 years of age *for sore throat* unless directed by a *doctor.*

*Please note that we have changed any reference from "physician" to "doctor".

From: **Ask a doctor before us if the child has**...not been drinking

To: **Ask a doctor before use if the child has**...not been drinking *fluids*

D. We have included the warning if stomach upset lasts or gets worse in the Stop use and ask a doctor section, as follows:

From: **Stop use and ask a doctor if**...stomach pain gets worse or lasts

To: **Stop use and ask a doctor if**...stomach pain *or upset* gets worse or lasts

E. <u>For clarity, we propose the following changes Under Directions:</u>

From: **Directions**...do not take more than directed

To: **Directions**...do not *give* more than directed

From: **Directions**...use only the enclosed dropper. Do not use any other dosing device. Fill to prescribed level.

To: **Directions**...use only *with* enclosed dropper. Fill to *dose* level. Do not use any other dosing device.

To emphasize the importance of using only the appropriate device to dose the product, we wish to include this information under Directions, as well as retain the current information following the dosing chart, i.e., "**Attention: Specifically designed for use with enclosed dropper. Use only enclosed dropper to dose this product. Do not use any other dosing device.**"

F. To be consistent with the 3/17/99 Final Rule on OTC labeling requirements for human drugs, we propose moving information concerning action to take in the event of stomach upset with use of the product to the appropriate subheading: "**When using this product**, give with food or milk if stomach upset occurs."

Therefore, this information is deleted from the Directions section (as last bullet: "if stomach upset occurs while taking this product; give with food or milk").

For your convenience, we have attached revised, draft labeling with the above changes. We trust we have adequately responded to your request. Should you have any questions, please call me at (215) 273-7115.

Sincerely,

McNEIL CONSUMER HEALTHCARE

Willie D. Pagsuyuin
Director, Regulatory Affairs

WDP:dtg
Attachment

cc: Kerry Rothschild (HFD-560)

p:\nda\corresp\bowen3.doc

APPEARS THIS WAY
ON ORIGINAL

DEPARTMENT OF HEALTH AND HUMAN SERVICES
FOOD AND DRUG ADMINISTRATION

APPLICATION TO MARKET A NEW DRUG, BIOLOGIC, OR AN ANTIBIOTIC DRUG FOR HUMAN USE

(Title 21, Code of Federal Regulations, 314 & 601)

Form Approved: OMB No. 0910-0338
Expiration Date: April 30, 2000
See OMB Statement on page 2.

FOR FDA USE ONLY

APPLICATION NUMBER

APPLICANT INFORMATION

NAME OF APPLICANT: McNeil CONSUMER HEALTHCARE

DATE OF SUBMISSION: APR 13 1999

TELEPHONE NO. *(Include Area Code)*: (215) 273-7115

FACSIMILE (FAX) Number *(Include Area Code)*: (215) 273-4049

APPLICANT ADDRESS *(Number, Street, City, State, Country, ZIP Code or Mail Code, and U.S. License number if previously issued)*: Camp Hill Road, Fort Washington PA 19034

AUTHORIZED U.S. AGENT NAME & ADDRESS *(Number, Street, City, State, ZIP Code, telephone & FAX number)* IF APPLICABLE

PRODUCT DESCRIPTION

NEW DRUG OR ANTIBIOTIC APPLICATION NUMBER, OR BIOLOGICS LICENSE APPLICATION NUMBER (If previously issued) 20-603

ESTABLISHED NAME *(e.g., Proper name, USP/USAN name)* Ibuprofen

PROPRIETARY NAME *(trade name)* IF ANY Children's MOTRIN Oral Drops

CHEMICAL/BIOCHEMICAL/BLOOD PRODUCT NAME *(If any)*

CODE NAME *(If any)*

DOSAGE FORM: Drops

STRENGTHS: 40mg/mL

ROUTE OF ADMINISTRATION: Oral

(PROPOSED) INDICATION(S) FOR USE: For the temporary relief of fever and minor aches and pains due to colds, flu, sore throat, headaches and toothaches

APPLICATION INFORMATION

APPLICATION TYPE (check one)
☒ NEW DRUG APPLICATION (21 CFR 314.50) ☐ ABBREVIATED APPLICATION (ANDA, AADA, 21 CFR 314.94)
☐ BIOLOGICS LICENSE APPLICATION (21 CFR part 601)

IF AN NDA, IDENTIFY THE APPROPRIATE TYPE ☒ 505 (b) (1) ☐ 505 (b) (2) ☐ 507

IF AN ANDA, OR AADA, IDENTIFY THE REFERENCE LISTED DRUG PRODUCT THAT IS THE BASIS FOR THE SUBMISSION
Name of Drug Holder of Approved Application

TYPE OF SUBMISSION (check one)
☐ ORIGINAL APPLICATION ☐ AMENDMENT TO A PENDING APPLICATION ☐ RESUBMISSION
☐ PRESUBMISSION ☐ ANNUAL REPORT ☐ ESTABLISHMENT DESCRIPTION SUPPLEMENT ☐ SUPAC SUPPLEMENT
☐ EFFICACY SUPPLEMENT ☐ LABELING SUPPLEMENT ☐ CHEMISTRY MANUFACTURING AND CONTROLS SUPPLEMENT ☒ OTHER

REASON FOR SUBMISSION
Corres: Response to FDA Comments

PROPOSED MARKETING STATUS (check one) ☐ PRESCRIPTION PRODUCT (Rx) ☒ OVER-THE-COUNTER PRODUCT (OTC)

NUMBER OF VOLUMES SUBMITTED ______

THIS APPLICATION IS ☒ PAPER ☐ PAPER AND ELECTRONIC ☐ ELECTRONIC

ESTABLISHMENT INFORMATION

Provide locations of all manufacturing, packaging and control sites for drug substance and drug product (continuation sheets may be used if necessary). Include name, address, contact, telephone number, registration number (CFN), DMF number, and manufacturing steps and/or type of testing (e.g. Final dosage form, Stability testing) conducted at the site. Please indicate whether the site is ready for inspection or, if not, when it will be ready.

Cross References (list related License Applications, INDs, NDAs, PMAs, 510(k)s, IDEs, BMFs and DMFs referenced in the current application)

CENTER FOR DRUG EVALUATION AND RESEARCH

APPLICATION NUMBER:

20-603/S-001, S-002, S-003

MEDICAL REVIEW

APR 1 4 1999

MEDICAL OFFICER REVIEW
Division of Over-The-Counter Drug Products

NDA #: 20-812, SE5-003
NAME: Pediatric Advil® (ibuprofen suspension) Drops100 mg/2.5 mL
SPONSOR: Whitehall-Robins
Five Giralda Farms
Madison, NJ 07940
Tel.:(973)660-5753
TYPE OF SUBMISSION: Commercial Pharmaceutical
DATE OF SUBMISSION: June 15, 1998 **CDER:** June 15, 1998
DATE OF REVIEW: March 22, 1999
REVIEWER: Rosemarie Neuner, MD, MPH
CSO: Mr. Kerry Rothschild, JD

Introduction

Ibuprofen is a propionic acid derivative that belongs to the nonsteroical anti-inflammatory class of drugs (NSAIDs). A suspension formulation of ibuprofen has been available as a prescription drug for use in children since 1989. On January 30, 1998 Pediatric Advil® (ibuprofen suspension) Drops, 100 mg/2.5 mL was approved by the U.S. Food and Drug Administration for marketing as an over-the-counter (OTC) drug product for the temporary relief of fever and pain in children 2-3 years of age. In June 1998, the sponsor of this product, Whitehall-Robins, submitted a request to the agency for a pediatric exclusivity claim which was subsequently granted. The sponsor has now submitted this efficacy supplement for agency review in which they request the lowering of the currently approved group age range from two to three years of age down to six months of age for this product.

In support of this change in the product's dosing age range, the sponsor has re-submitted the data generated from 41,810 children who participated in the actual use drug safety trial, the Children's Analgesic Medicine Project (CAMP), which evaluated Children's Advil as an antipyretic and analgesic agent. [Note: This was the pivotal safety study that supported the approval for the sponsor's Pediatric Advil® (ibuprofen) Suspension 100 mg/5 mL (NDA 20-589) in 1995, and for the sponsor's Pediatric Advil® (ibuprofen) Drops100 mg/2.5 mL (NDA 20-812) in 1998.] The sponsor has included additional new safety information in this review regarding the occurrence of anaphylaxis, gastrointestinal (GI) bleeding, renal failure, and Reye syndrome in this study's population. In addition, the sponsor has submitted in support of this SNDA a published article from a peer reviewed journal which describes the results from the actual use Boston Fever Study in which the safety profile of pediatric ibuprofen was compared to that of acetaminophen in over 84,000 children with fever, and as well as the overheads used by Dr. Larry Lesko to present the new subcohort analysis of children ≤ 2 years of age from that study at the September 18,1998 NDAC. They have also included for review 1 article which describes a pharmacokinetic (PK) study in febrile children between the ages 3 months through 12 years, and resubmitted the

results of the PK Study AF-95-04 which demonstrated the bioequivalency of the sponsor's 2 pediatric formulations in adult volunteer's [Children's Advil® Suspension (NDA 20-289) to Pediatric Advil® Drops (NDA 20-812)]. Since the latter study was reviewed by the agency in support of the sponsor's bioequivalency claim, it will not be re-reviewed at this time, but the results from the published PK study are discussed in the PK section of this SNDA review by Dr. E. Dennis Bashaw, FDA Division of Pharmacokinetics (HFD-880).

Since prescription ibuprofen is currently approved for use in infants age 6 months and older, the major regulatory issue to be answered by this application is the safety of OTC ibuprofen for use in the pediatric age group between 6 months and 2 years of age, as the other doses proposed by the sponsor of this supplement have been previously approved for OTC use. This review will therefore concentrate on the drug's safety profile in this targeted age group since the sponsor did not submit any additional inform regarding ibuprofen's efficacy.

Safety

The focus of this review is to determine whether ibuprofen is safe to be used as an OTC agent in the sponsor's requested targeted pediatric age group of 6-months to 2-years. In support of this product's safety profile the sponsor has submitted for review the following safety data for children less than 2-years of age:

1. A new analysis of serious adverse events (i.e., ≥ 1%) as well as acute gastrointestinal bleeding, renal failure, and Reye syndrome that occurred during the Children's Analgesic Medicine Project (CAMP).
2. A copy of the original article from a peer-reviewed journal of the Boston University Fever Study.
3. The overheads used at the September 18,1998 NDAC presentation by Dr. Larry Lesko of the subgroup analysis of children less than 2-years of age from the Boston University Fever Study.

The sponsor did not submit any postmarketing adverse event information, overdose case reports, or other data from the worldwide literature as part of the global safety update for this product. The analysis of serious adverse events from the CAMP study will be discussed first followed by a brief discussion of the Boston University Fever Study, and the new subcohort analysis in children ≤ 2 years of age which was presented at the September 18, 1998 NDAC meeting.

1. Analysis of serious adverse events (i.e., death, anaphylaxis, gastrointestinal bleeding, renal failure, Reye syndrome, and any event with a frequency ≥ 1%) that occurred during the Children's Analgesic Medicine Project (CAMP).

(Note: This study has been reviewed and discussed in detail previously by agency reviewers in support of regulatory actions taken on NDA 20-589 and NDA 20-812. The

following is a brief overview of the study with a discussion of serious adverse events ≥ 1%, as well as any episodes of acute gastrointestinal bleeding, renal failure, and Reye syndrome that occurred in children < 2 years of age while participating in the trial.)

The Children's Analgesic Medicine Project (CAMP) study was a multi-center, multi-dose, open-label, nonrandomized, acetaminophen-controlled study conducted by [redacted], an affiliate of the University of Utah in an office-based pediatric population from the continental United States. The study's objective was to evaluate the actual clinical experience with Children's Advil® as compared to acetaminophen. Children who required treatment with either an antipyretic or analgesic were entered into the study by 424 health care providers from 69 pediatric centers during the time period from March 1993 through July 1995. A total of 41,810 children were entered, out of which 14,291 children < 2 years of age were treated with at least 1 dose of medicine and had follow-up information. Of these 14,291 children < 2 years of age, 7,381 children were treated with ibuprofen while the remaining 6,900 children were treated with acetaminophen. (Note: Treatment assignments of the study participants were made by their health care providers, and were to have reflected the latters usual therapeutic preferences.) Information regarding study drug exposure was collected at two time points during the trial: at the time of enrollment and at 1-2 weeks into the study via a telephone interview with the child's care giver.

The only demographic information that this study collected from patients enrolled into the trial was for the child's age and reason for enrollment (i.e., sick or well child visit). "Sick child" visits were for a variety of reasons. Some of the more frequently occurring reasons are listed as follows: otitis media, pharyngitis, viral illness, pain, upper respiratory tract infections, and bronchitis. "Well child" visits included prophylactic treatment following routine immunizations. Drug exposure ranged from a single dose (which occurred in approximately 7% of the total population entered) to more than 21 doses (which occurred in approximately 4% of the total population studied.)

The new safety analysis looked at the numbers of serious adverse events that occurred during the study which included: deaths, anaphylaxis, GI bleeding, renal failure, Reye syndrome, and any serious adverse event that occurred in ≥ 1% of the treated study population.

1.A. Deaths - Four (4) children < 2 years of age died while enrolled in the CAMP study. Two (2) of the 4 children were treated with ibuprofen, 1 received acetaminophen and 1 was assigned to take acetaminophen but never did take the study drug. The first case (Case Number 034141Y) involved a 1-year-old female evaluated for a viral syndrome associated with a fever of 104°. The child was assigned to treatment with 1 teaspoon of ibuprofen every 6 hours which was alternated with acetaminophen. (Note: Reason for administering both study medications was not stated.) She was treated with this regimen over a 3-day period of time following which she developed seizures, was hospitalized and placed on mechanical ventilation. She was diagnosed as having herpetic encephalitis and died 48 hours after admission to the hospital. An autopsy was not performed.

The second-death (Case Number 011410Y) occurred in a 11-month-old male patient evaluated earlier for otitis media associated with a fever. The patient was assigned to receive treatment with ibuprofen and an unknown antibiotic. Over the next 48-hour period of time, the child deteriorated and was admitted to the hospital with a diagnosis of bacterial *Strep. pneumoniae* meningitis. He recovered and was discharged home 10 days later, only to be readmitted approximately 10 days post discharge with septicemia due to the same organism. The child died 48 hours following the second admission and was found on autopsy to have congenital asplenia.

Of the remaining 2 deaths, one occurred (Case Number 032524) in a 23-month-old female seen for a viral illness associated with a headache. She was assigned to treatment with acetaminophen, but according to the information supplied by the sponsor, never received the study medication. A few months later she presented for follow-up with a history of constitutional complaints which included drowsiness and sluggishness. A CT scan of the patient's head demonstrated a large cerebellar medulloblastoma which hemorrhaged before resection could be performed. The child subsequently died due to complications from her brain tumor.

The fourth death occurred (Case Number 005035Y) in a 3-month-old male who was assigned to receive treatment with acetaminophen for prophylaxis following immunization. This patient received 7-10 doses over a 7-day period of time of the study medication. The child died 2 ½ weeks after finishing treatment with the study medication. The cause of death at autopsy was noted to be Sudden Infant Death Syndrome (SIDS).

B. Anaphylaxis - There were no cases of anaphylaxis reported to have occurred in children < 2 years of age in the CAMP Study.

C. Gastrointestinal (GI) Bleeding - There were no cases of GI bleeding reported to have occurred in children < 2 years of age in the CAMP Study.

D. Renal Failure - There were no cases of renal failure reported to have occurred in children < 2 years of age in the CAMP Study.

E. Reye syndrome - There were no cases of Reye syndrome reported to have occurred in children < 2 years of age in the CAMP Study.

F. Serious Adverse Experiences Occurring in ≥ 1% of Treated Subjects in the CAMP Study.

There were no serious adverse events reported to have occurred in ≥ 1% of treated patients enrolled in the CAMP Study. In Attachment I, at the end of this review, are tables prepared by the sponsor which summarize the number of serious adverse events as related to study treatment which occurred in children < 2 years of age who participated in this study. The most commonly reported serious adverse event in children < 2 years of age treated with ibuprofen were elective hospitalizations (0.46%) for the insertion of pressure equalizing tubes in the Eustachian tubes of some of the

children while being treated for otitis media.

<u>Medical Reviewer's Comments:</u> *This limited post hoc analysis of serious adverse events in children between the ages of 6 months to 2 years enrolled in the CAMP study is plagued by a multitude of methodological flaws, thus raising questions regarding the validity of its findings. At best this reviewer can say that based on the paucity of drug related serious adverse events which occurred in this large pediatric study, ibuprofen suspension is safe to be used in an OTC pediatric population between the ages of 6 months to 2 years of age.*

2. Lesko SL, Mitchell AA: An Assessment of the Safety of Pediatric Ibuprofen: A Practitioner-Based Randomized Clinical Trial. JAMA, 273(12):929-933, 1995.

In support of their request, the sponsor sent in a copy of the above article published in a peer-reviewed journal which describes the original Boston Fever Study. (Note: A full synopsis of this study by this medical reviewer can be found at the end of this review in Attachment II.) This was a 4-week, multi center, double-blind, randomized, acetaminophen-controlled antipyretic study in an office-based pediatric population from the continental United States. The study's objective was to assess the risk of hospitalization due to serious adverse events such as gastrointestinal bleeding, acute renal failure, anaphylaxis and Reye syndrome associated with the use of ibuprofen in febrile children. A total of 84,192 children between the ages of 6 months to 12 years were enrolled in the study, but data was available for final analysis on only 83,915 children who had been randomized into 1 of the 3 treatment groups as follows: 5 mg/kg ibuprofen (n=27,948), 10 mg/kg ibuprofen (n=27,837), and 12 mg/kg acetaminophen (n=28,130). Although all 3 treatment groups were similar for various demographic parameters, the article did not give the number of children < 2 years of age who participated in the study.

During the trial there were only 4 reported cases of GI bleeding, and no cases of acute renal failure, anaphylaxis, or Reye Syndrome. All 4 cases of GI bleeding occurred in children treated with ibuprofen (2 from the high-dose group and 2 from the low dose group). (Note: The article did not state the ages of these 4 children.) The observed risk for developing GI bleeding due to treatment with ibuprofen in this study was calculated to be 7.2 per 100,000 (95% confidence interval, 2 to 18 per 100,000). On comparison analysis, this risk was not found to be significantly different (p=0.31) from the risk for developing GI bleeding in the acetaminophen treated group (0 per 100,000; 95% confidence interval, 0 to 11 per 100,000). The observed risk for developing acute renal failure, anaphylaxis, or Reye's syndrome was calculated for all of the children (n=55,785) treated with ibuprofen in the study and was found to be 0 per 100,000 (95% confidence interval, 0 to 5.4 per 100,000 ibuprofen-treated children).

Based on these findings, the authors conclude that the short-term risk for hospitalization due to GI bleeding, acute renal failure, anaphylaxis and Reye's syndrome associated with the use of high and low dose ibuprofen in children in this trial was not any different from the risk in children treated with acetaminophen.

Medical Reviewer's Comments: Unfortunately, this published pediatric study does not list the number of children < 2 years of age who participated in the trial. This information would have been very helpful since the sponsor has requested that their pediatric formulation be labeled for use in children 6 months of age and older. Thus, this study can provide only supportive evidence of the safety profile of ibuprofen in children < 2 years of age.

3. The overheads used at the September 18,1998 NDAC presentation by Dr. Larry Lesko of the subgroup analysis of children less than 2-years of age from the Boston University Fever Study.

Dr. Larry Lesko, the first author of the published article submitted by the sponsor which was reviewed and commented on in the preceding section of this review, presented a subcohort analysis of children < 2 years of age who participated in the Boston Fever Study at the September 18, 1998 NDAC meeting. (Refer to the above Safety Section 2 and the study synopsis in Attachment II found at the end of this review for further information regarding this study. Reproductions of these overheads can be found in Attachment III at the end of this review.)

This post hoc subcohort analysis revealed that there were 27,065 children < 2 years of age who participated in the Boston Fever Study, out of which 17,938 were treated with ibuprofen and 9,127 received acetaminophen. A total of 261 children out of the 17,938 children in the ibuprofen treatment group were hospitalized during the study [observed risk for hospitalization due to any cause: 1.5 per 100,000; 95% confidence interval (1.3 to 1.6 per 100,000)] as compared to 124 out of 9,127 from the acetaminophen group [observed risk for hospitalization due to any cause: 1.4 per 100,000; 95% confidence interval (1.1 to 1.6 per 100,000)]. The risk for hospitalization in children <2 years of age was not significantly greater when compared to the corresponding risk for hospitalization in children > 2 years of age (n=56,850) who participated in the study (observed risk: 2.6 per 100, 000; confidence interval not given).

There were 3 cases of GI bleeding reported to have occurred in the subcohort population < 2 years of age. (Note: The overheads do not provide any additional information regarding these 3 cases.) The observed risk for hospitalization due to GI bleeding in children < 2 years of age in the subcohort was found to be 17 per 100,000 with a 95% confidence interval (CI) of 3.5 to 49 per 100,000 as compared to an observed risk of 0 per 100,000 with 95% CI of 0 to 33 per 100,000 for children < 2 years of age treated with acetaminophen.

Although there were no cases reported of renal failure, anaphylaxis or Reye syndrome in children < 2 years of age who participated in this study, the observed risk for hospitalization due to any of these 3 other primary outcomes were calculated for both treatment groups and shown to be as follows: ibuprofen treatment group: [0 per 100,000 (95% CI: 0 to 17 cases per 100,000)]; acetaminophen treatment group: [0 per 100,000 (95% CI: 0 to 33 cases per 100,000)]. Due to additional safety concerns for hospitalizations due to asthma, bronchiolitis and vomiting/gastritis in this age group, Dr.

Lesko also presented the calculated observed risks for hospitalizations that occurred during the study due to these 3 illnesses which were similar for both treatment groups as shown in the following table, Table 1:

Table 1 - Risk for Hospitalization Due to Secondary Outcomes in Children < 2 Years of Age in the Boston Fever Study

Diagnosis	Ibuprofen	Acetaminophen
**Total Number**	17,938	9,127
**Asthma, No.**<br>**Risk/100,000**<br>**(95% CI)**	20<br>110<br>(68-172)	12<br>130<br>(70-230)
**Bronchiolitis, No.**<br>**Risk/100,000**<br>**(95% CI)**	21<br>120<br>(72-180)	12<br>130<br>(70-230)
**Vomiting/Gastritis, No.**<br>**Risk/100,000**<br>**(95% CI)**	7<br>39<br>(16-80)	2<br>22<br>(2.6-79)

Since there were no cases of acute renal failure reported to have occurred during this study, Dr. Lesko presented additional data from 112 children < 2 years of age who had serum creatinines drawn during study hospitalizations in an attempt to see if this surrogate marker for renal function would show any treatment associated nephrotoxicity in this subcohort age group. Table 2, shown below, shows the limited data presented at the NDAC meeting by the author regarding elevated serum creatinines in children < 2 years of age. (Note: No statistical analysis of the serum creatinine data was submitted for review by the presenter or the sponsor.)

Table 2 - Elevated Serum Creatinines in Hospitalized Children < 2 Years of Age in the Boston Fever Study

	Ibuprofen	Acetaminophen
**Total Number**	83	29
**Serum creatinine, mg/dL**<br>**Mean**<br>**(SEM)**<br>**Range**	 0.42<br>(0.023) 	 0.34<br>(0.025)
**Serum creatine**<br>**> 0.7mg/dL**<br>**No.**<br>**(%)**	 <br>5<br>(6)	 <br>0<br>(0)

Based on the data that he presented, Dr. Lesko concluded that in the Boston Fever Study subcohort of children < 2 years of age (n=27,065), the use of ibuprofen suspension was not associated with an increased risk of hospitalization overall or for acute GI bleeding, acute renal failure, anaphylaxis or Reye syndrome compared to acetaminophen (n=9,127).

<u>Medical Reviewer's Comments:</u> *The above subcohort analysis provides the safety data in children < 2 years of age which was missing from the original published article by Lesko et al, that was submitted by the sponsor and discussed and commented in the above Section 2 of this review. Since not all of the children who were hospitalized had serum creatinines drawn, this medical reviewer feels it would be inappropriate to comment on the significance of the serum creatinine data especially since no background information was provide regarding the 5 cases involved.*

[illegible]

APPEARS THIS WAY
ON ORIGINAL

APPEARS THIS WAY
ON ORIGINAL

Recommendations: Based on the data contained in this submission generated from a major clinical pediatric study performed by the sponsor, as well as data from another published major pediatric study, Pediatric Advil® (ibuprofen suspension) Drops100 mg/2.5 mL appears to be safe to be used in an OTC pediatric population > 6 months of age. According to agency regulations regarding the contents of efficacy submissions, the sponsor has failed to submit complete safety data for this product which should include the following missing data: Whitehall-Robins adverse event monitoring system controlled clinical trial data on subjects ≤ 2 years of age enrolled in studies treated with ibuprofen since the completion of the CAMP study, reports of serious adverse events collected by Whitehall-Robins adverse event monitoring system which have occurred in children ≤ 2 years of age from the time of the sponsor's last submission for their pediatric ibuprofen products to the present, reports of serious adverse events collected by the FDA's Spontaneous Reporting System (SRS) for all ibuprofen products in children ≤ 2 years of age for the same time period, overdose data from the American Association of Poison Control Centers (AAPCC) for ibuprofen containing products in children ≤ 2 years of age, and information regarding foreign marketing of this product for the requested age-related indication. Thus, this medical officer can only recommend that this application be designated as approvable pending the sponsor submission and review of the missing required safety data.

If and when this application receives agency approval for the marketing of this indication, an overlap in dosing age ranges should be avoided for this product and its sister product, Children's Advil® (ibuprofen suspension)100 mg/5 mL because of the possible threat of dosing misadventures due to consumer confusion. Thus, the concentrated drops should be labeled for use in children ≤ 2 years of age, and the less concentrated solution should be labeled for use in children ≥ 2 years of age. To further help prevent these incidents from happening in the future, the sponsor needs to re-label this product as "concentrate" as follows: Pediatric Advil® (ibuprofen suspension) Concentrated Drops100 mg/2.5 mL

/S/

Rosemarie Neuner, MD, MPH
Medical Reviewer, HFD-560

/S/

Linda M. Katz, MD, MPH 4/14/99
Deputy Dir., HFD-560

CC: NDA 20-603 File
HFD-560 Div. File
HFD-550 Div. File
HFD-560 ActingDir/Bowen
HFD-560 Dep Dir/Katz
HFD-560 Team Leader/Lumpkins
HFD-550 Team Leader/Hyde
HFD-560 MO/Neuner
HFD-560 PM/KRothschild

Appendix I

DATA ON THE USE OF IBUPROFEN SUSPENSION
AND ORAL DROPS IN CHILDREN AGES
6 MONTHS THROUGH 2 YEARS

Attachment B

Summary of Serious Adverse Experiences (AEs) by Relationship of AE to Study Medication

APPEARS THIS WAY
ON ORIGINAL

APPEARS THIS WAY
ON ORIGINAL

APPEARS THIS WAY
ON ORIGINAL

Whitehall-Robins Healthcare
Protocol #: CAMP I
Investigator: Pooled

CAMP I Study

Table B.3a.1

Summary of Serious Adverse Experiences (AEs) by Relationship of AE to Study Medication

For All Children under 2 Years

Who Took at Least One Dose of Study Medication

(Took Ibuprofen or Acetaminophen Only)

Adverse Experience(AEs) (by Body System and Event Term)	Ibuprofen (n=7381) No. of AEs(%)	Ibuprofen RELATIONSHIP Drug Related +	Ibuprofen RELATIONSHIP Not Related	Ibuprofen RELATIONSHIP Missing	Ibuprofen RELATIONSHIP Overdose	Acetaminophen (n=6900) No. of AEs(%)	Acetaminophen RELATIONSHIP Drug Related +	Acetaminophen RELATIONSHIP Not Related	Acetaminophen RELATIONSHIP Missing	Acetaminophen RELATIONSHIP Overdose
Any Body System										
No. of AEs**	231	10	196	23	2	118	6	98	10	4
No. of subjects**	129 (1.75)	7	118	2	2	68 (0.99)	4	59	1	4
Body as a Whole										
SURGICAL PROCEDR	52 (0.70)	0	52	0	0	24 (0.35)	0	24	0	0
FEVER	7 (0.09)	0	7	0	0	2 (0.03)	0	2	0	0
SEPSIS	4 (0.05)	0	4	0	0	1 (0.01)	0	1	0	0
CELLULITIS	2 (0.03)	0	2	0	0	0 (0.00)	0	0	0	0
INFECT	2 (0.03)	0	2	0	0	2 (0.03)	0	2	0	0
OVERDOSE	2 (0.03)	0	0	0	2	3 (0.04)	0	0	0	3
PAIN	2 (0.03)	0	2	0	0	0 (0.00)	0	0	0	0
PAIN BACK	2 (0.03)	0	2	0	0	0 (0.00)	0	0	0	0
ABSCESS	1 (0.01)	0	1	0	0	0 (0.00)	0	0	0	0
DEATH	1 (0.01)	0	1	0	0	0 (0.00)	0	0	0	0
HEADACHE	1 (0.01)	0	1	0	0	0 (0.00)	0	0	0	0
HERNIA	1 (0.01)	0	1	0	0	0 (0.00)	0	0	0	0
INJURY ACCID	1 (0.01)	0	1	0	0	0 (0.00)	0	0	0	0
OVERDOSE ACCID	1 (0.01)	0	1	0	0	4 (0.06)	0	3	0	1
No. of AEs**	79	0	77	0	2	36	0	32	0	4
No. of subjects**	70 (0.95)	0	68	0	2	36 (0.52)	0	32	0	4
Cardiovascular										
CARDIAC SURGERY	0 (0.00)	0	0	0	0	1 (0.01)	0	1	0	0
No. of AEs**	0	0	0	0	0	1	0	1	0	0
No. of subjects**	0 (0.00)	0	0	0	0	1 (0.01)	0	1	0	0

See last page of the table for footnotes.

Whitehall-Robins Healthcare
Protocol #: CAMP I
Investigator: Pooled

CAMP I Study

Table B.3a.1 (Cont'd)

Summary of Serious Adverse Experiences (AEs) by Relationship of AE to Study Medication

For All Children under 2 Years

Who Took at Least One Dose of Study Medication

(Took Ibuprofen or Acetaminophen Only)

Adverse Experience (AEs) (by Body System and Event Term)	Ibuprofen (n=7381)					Acetaminophen (n=6900)				
		RELATIONSHIP					RELATIONSHIP			
	No. of AEs(%)	Drug Related +	Not Related	Missing	Overdose	No. of AEs(%)	Drug Related +	Not Related	Missing	Overdose
**Digestive**										
VOMIT	8 (0.11)	3	5	0	0	7 (0.10)	0	7	0	0
DIARRHEA	5 (0.07)	1	3	1	0	4 (0.06)	0	4	0	0
ANOREXIA	1 (0.01)	0	1	0	0	0 (0.00)	0	0	0	0
DIARRHEA BLOODY	1 (0.01)	0	1	0	0	0 (0.00)	0	0	0	0
STOMATITIS	1 (0.01)	0	1	0	0	0 (0.00)	0	0	0	0
CONSTIP	0 (0.00)	0	0	0	0	1 (0.01)	0	1	0	0
DYSPHAGIA	0 (0.00)	0	0	0	0	1 (0.01)	0	1	0	0
GASTROENTERITIS	0 (0.00)	0	0	0	0	1 (0.01)	0	1	0	0
PAIN ABDO	0 (0.00)	0	0	0	0	1 (0.01)	0	1	0	0
No. of AEs**	16	4	11	1	0	15	0	15	0	0
No. of subjects**	13 (0.18)	4	8	1	0	9 (0.13)	0	9	0	0
**Hematic and Lymphatic**										
CYANOSIS	1 (0.01)	0	1	0	0	0 (0.00)	0	0	0	0
No. of AEs**	1	0	1	0	0	0	0	0	0	0
No. of subjects**	1 (0.01)	0	1	0	0	0 (0.00)	0	0	0	0
**Metabolic and Nutritional**										
DEHYDRAT	28 (0.38)	2	24	2	0	6 (0.09)	1	5	0	0
No. of AEs**	28	2	24	2	0	6	1	5	0	0
No. of subjects**	28 (0.38)	2	24	2	0	6 (0.09)	1	5	0	0

See last page of the table for footnotes.

Whitehall-Robins Healthcare
Protocol #: CAMP I
Investigator: Pooled

CAMP I Study

Table B.3a.1 (Cont'd)

Summary of Serious Adverse Experiences (AEs) by Relationship of AE to Study Medication

For All Children under 2 Years

Who Took at Least One Dose of Study Medication

(Took Ibuprofen or Acetaminophen Only)

Adverse Experience (AEs) (by Body System and Event Term)	Ibuprofen (n=7381)					Acetaminophen (n=6900)				
		RELATIONSHIP					RELATIONSHIP			
	No. of AEs(%)	Drug Related +	Not Related	Missing	Overdose	No. of AEs(%)	Drug Related +	Not Related	Missing	Overdose
Musculoskeletal										
OSTEOMYELITIS	2 (0.03)	0	2	0	0	1 (0.01)	0	1	0	0
SYNOVITIS	0 (0.00)	0	0	0	0	1 (0.01)	0	1	0	0
No. of AEs**	2	0	2	0	0	2	0	2	0	0
No. of subjects**	2 (0.03)	0	2	0	0	2 (0.03)	0	2	0	0
Nervous System										
CONVULS	4 (0.05)	0	4	0	0	1 (0.01)	1	0	0	0
MENINGITIS	3 (0.04)	0	3	0	0	1 (0.01)	0	1	0	0
NERVOUSNESS	1 (0.01)	0	1	0	0	0 (0.00)	0	0	0	0
SOMNOLENCE	1 (0.01)	0	0	1	0	0 (0.00)	0	0	0	0
Unknown Event	1 (0.01)	0	1	0	0	0 (0.00)	0	0	0	0
No. of AEs**	10	0	9	1	0	2	1	1	0	0
No. of subjects**	9 (0.12)	0	9	0	0	2 (0.03)	1	1	0	0

See last page of the table for footnotes.

Whitehall-Robins Healthcare
Protocol #: CAMP I
Investigator: Pooled

CAMP I Study

Table B.3a.1 (Cont'd)

Summary of Serious Adverse Experiences (AEs) by Relationship of AE to Study Medication

For All Children under 2 Years

Who Took at Least One Dose of Study Medication

(Took Ibuprofen or Acetaminophen Only)

Adverse Experience (AEs) (by Body System and Event Term)	Ibuprofen (n=7381)					Acetaminophen (n=6900)				
		RELATIONSHIP					RELATIONSHIP			
	No. of AEs(%)	Drug Related +	Not Related	Missing	Overdose	No. of AEs(%)	Drug Related +	Not Related	Missing	Overdose
Respiratory										
PNEUMONIA	19 (0.26)	0	19	0	0	11 (0.16)	0	9	2	0
ASTHMA	8 (0.11)	1	7	0	0	12 (0.17)	1	10	1	0
BRONCHIOLITIS	7 (0.09)	1	6	0	0	4 (0.06)	0	4	0	0
LUNG DIS	7 (0.09)	0	7	0	0	5 (0.07)	0	5	0	0
DYSPNEA	6 (0.08)	1	5	0	0	4 (0.06)	0	4	0	0
LARYNGITIS	3 (0.04)	1	2	0	0	2 (0.03)	0	2	0	0
COUGH INC	2 (0.03)	0	2	0	0	2 (0.03)	0	2	0	0
BRONCHITIS	1 (0.01)	0	1	0	0	1 (0.01)	0	1	0	0
HYPOVENTIL	1 (0.01)	0	0	1	0	0 (0.00)	0	0	0	0
PHARYNGITIS	1 (0.01)	0	1	0	0	0 (0.00)	0	0	0	0
SINUSITIS	1 (0.01)	0	0	1	0	0 (0.00)	0	0	0	0
STRIDOR	1 (0.01)	0	1	0	0	1 (0.01)	0	0	1	0
ATELECTASIS	0 (0.00)	0	0	0	0	2 (0.03)	1	0	1	0
HYPERVENTIL	0 (0.00)	0	0	0	0	1 (0.01)	1	0	0	0
HYPOXIA	0 (0.00)	0	0	0	0	1 (0.01)	1	0	0	0
No. of AEs**	57	4	51	2	0	46	4	37	5	0
No. of subjects**	37 (0.50)	2	33	2	0	24 (0.35)	2	21	1	0
Skin										
RASH	1 (0.01)	0	1	0	0	0 (0.00)	0	0	0	0
No. of AEs**	1	0	1	0	0	0	0	0	0	0
No. of subjects**	1 (0.01)	0	1	0	0	0 (0.00)	0	0	0	0

See last page of the table for footnotes.

Whitehall-Robins Healthcare
Protocol #: CAMP I
Investigator: Pooled

CAMP I Study

Table B.3a.1 (Cont'd)

Summary of Serious Adverse Experiences (AEs) by Relationship of AE to Study Medication

For All Children under 2 Years

Who Took at Least One Dose of Study Medication

(Took Ibuprofen or Acetaminophen Only)

Adverse Experience (AEs) (by Body System and Event Term)	Ibuprofen (n=7381)					Acetaminophen (n=6900)				
		RELATIONSHIP					RELATIONSHIP			
	No. of AEs(%)	Drug Related +	Not Related	Missing	Overdose	No. of AEs(%)	Drug Related +	Not Related	Missing	Overdose
Special Senses										
OTITIS MED	34 (0.46)	0	17	17	0	9 (0.13)	0	4	5	0
No. of AEs**	34	0	17	17	0	9	0	4	5	0
No. of subjects**	34 (0.46)	0	17	17	0	9 (0.13)	0	4	5	0
Uro-genital										
INFCT URIN TRACT	2 (0.03)	0	2	0	0	1 (0.01)	0	1	0	0
PYELONEPHRITIS	1 (0.01)	0	1	0	0	0 (0.00)	0	0	0	0
No. of AEs**	3	0	3	0	0	1	0	1	0	0
No. of subjects**	3 (0.04)	0	3	0	0	1 (0.01)	0	1	0	0

** A subject may have multiple AEs for each COSTART term. The number of AEs includes ALL Events. The number of subjects, however, counts a subject only ONCE within each body system. AEs are classified by the worst relationship to study medication.
+: Possibly, Probably, or Definitely.
Note: 'Not Related' includes AE-REMOTE, NO AE-PRI ILLNES, NO AE-CHILD ILL, NO AE-MED SURG, NO AE-SX>5 DAYS, NO AE-SX BEF TX, NO AE-MISCOMM, NO F-UP INFO, and NO AE-ANY REASON.

Whitehall-Robins Healthcare
Protocol #: CAMP I
Investigator: Pooled

CAMP I Study

Table B.3a.1 (Cont'd)

Summary of Serious Adverse Experiences (AEs) by Relationship of AE to Study Medication

For All Children under 2 Years

Who Took at Least One Dose of Study Medication

(Took Ibuprofen or Acetaminophen Only)

Adverse Experience (AEs) (by Body System and Event Term)	Ibuprofen (n=7381)					Acetaminophen (n=6900)				
	RELATIONSHIP					RELATIONSHIP				
	No. of AEs(%)	Drug Related +	Not Related	Missing	Overdose	No. of AEs(%)	Drug Related +	Not Related	Missing	Overdose
Special Senses										
OTITIS MED	34 (0.46)	0	17	17	0	9 (0.13)	0	4	5	0
No. of AEs**	34	0	17	17	0	9	0	4	5	0
No. of subjects**	34 (0.46)	0	17	17	0	9 (0.13)	0	4	5	0
Uro-genital										
INFCT URIN TRACT	2 (0.03)	0	2	0	0	1 (0.01)	0	1	0	0
PYELONEPHRITIS	1 (0.01)	0	1	0	0	0 (0.00)	0	0	0	0
No. of AEs**	3	0	3	0	0	1	0	1	0	0
No. of subjects**	3 (0.04)	0	3	0	0	1 (0.01)	0	1	0	0

** A subject may have multiple AEs for each COSTART term. The number of AEs includes ALL Events. The number of subjects, however, counts a subject only ONCE within each body system. AEs are classified by the worst relationship to study medication.
+: Possibly, Probably, or Definitely.
Note: 'Not Related' includes AE-REMOTE, NO AE-PRI ILLNES, NO AE-CHILD ILL, NO AE-MED SURG, NO AE-SX>5 DAYS, NO AE-SX BEF TX, NO AE-MISCOMM, NO F-UP INFO, and NO AE-ANY REASON.

Appendix II

Lesko SL, Mitchell AA: An Assessment of the Safety of Pediatric Ibuprofen: A Practitioner-Based Randomized Clinical Trial. JAMA, 273(12):929-933, 1995.

This was a 4-week, multi center, double-blind, randomized, acetaminophen-controlled antipyretic study in an office-based pediatric population from the continental United States. The study's objective was to assess the risk of hospitalization due to serious adverse events such as gastrointestinal bleeding, acute renal failure, anaphylaxis and Reye syndrome associated with the use of ibuprofen in febrile children. A total of 84,192 children between the ages of 6 months to 12 years weighing 7-50 kg were recruited after presenting for a pediatric evaluation of an acute febrile illness to any one of the 1,735 pediatricians or family practitioners participating in the trial. In order to be eligible for study entry, the children had to be able to take the study medication by mouth, and have a parent/guardian administer the study medication while observing and caring for them. Children who were dehydrated, unable to take medication by mouth, or with histories of hypersensitivity to acetaminophen or NSAIDS, renal or hepatic diseases, bleeding disorders, anemia, neoplasia, endocrine or metabolic problems, or peptic ulcer disease were ineligible for study entry. Participants were randomized to receive 1 of the following 3 study treatments: 5 mg/kg ibuprofen, 10 mg/kg ibuprofen, or 12 mg/kg acetaminophen.

Results: Two hundred seventy-seven (277, 0.3%) of the 84,192 children enrolled in the study were lost to follow-up and were not included in the final data analysis. Of the 83,915 children in which there was data available for study analysis, 27,948 were treated with 5mg/kg ibuprofen, 27,837 were treated with 10mg/kg ibuprofen, and 28,130 were treated with acetaminophen. All 3 treatment groups were found to be demographically similar with respect to age, weight, sex, and race. The most frequently reported causes of the presenting fever for all 3 treatment groups are as follows: upper respiratory tract infection, otitis media, pharyngitis, lower respiratory tract and gastrointestinal tract infections. A total of 795 (1%) children were hospitalized during the study. The hospitalization rates were found to be similar for all 3 treatment groups as follows: 0.9% for the 5 mg/kg ibuprofen group, 1% for the 10 mg/kg ibuprofen group, and 1% for the 12 mg/kg acetaminophen group. During the study there were only 4 reported cases of GI bleeding, and no cases of acute renal failure, anaphylaxis, or Reye Syndrome. All 4 cases of GI bleeding occurred in children treated with ibuprofen (2 from the high-dose group and 2 from the low dose group). The observed risk for developing GI bleeding due to treatment with ibuprofen in this study was calculated to be 7.2 per 100,000 (95% confidence interval, 2 to 18 per 100,000). On comparison analysis, this risk was not found to be significantly different (p=0.31) from the risk in the acetaminophen treated group (0 per 100,000; 95% confidence interval, 0 to 11 per 100,000). The observed risk for developing acute renal failure, anaphylaxis, or Reye's syndrome was calculated for all of the children treated with ibuprofen (n=55,785) in the study and found to be 0 per 100,000 (95% confidence interval, 0 to 5.4 per 100,000 ibuprofen-treated children). The remaining 112 serious hospitalizations were for a variety of adverse events as follows: asthma (n=68), vomiting or gastritis (n=26),

neutropenia (n=9), erythema multiforme (n=4), abdominal pain (n=4), serum sickness (n=1). Of these 6 conditions, only the risk for developing neutropenia in the ibuprofen group (14 per 100,000; 95% confidence interval, 6.2 to 28 per 100,000) was found to be significantly different (p=0.4) from the risk in the acetaminophen treated group (0 per 100,000; 95% confidence interval, 0 to 11 per 100,000). The authors state that this unexpected association was noted after multiple comparisons and that they do not know what to make of it since pretreatment white blood cell counts were not obtained.

Conclusion: The short-term risk for hospitalization due to GI bleeding, acute renal failure, anaphylaxis and Reye's syndrome associated with the use of high and low dose ibuprofen in children in this trial was not shown to be any different from the risk in children treated with acetaminophen. Although children treated with ibuprofen in this study were shown to have an unexpected increased risk for hospitalization due to neutropenia as compared to children treated with acetaminophen, the significance of this finding is unclear since pretreatment white cell counts were not obtained for comparison.

APPEARS THIS WAY
ON ORIGINAL

APPEARS THIS WAY
ON ORIGINAL

16 page(s) have been removed because it contains trade secret and/or confidential information that is not disclosable.

MAR 2 9 1999

MEDICAL OFFICER REVIEW
Division of Over-The-Counter Drug Products

NDA #: 20-603, SE3-003
NAME: Children's MOTRIN® (ibuprofen oral suspension) Drops, 50 mg/1.25 mL
SPONSOR: McNeil Consumer Products Company
7050 Camp Hill Road
Fort Washington, PA 19034-2299
Tel.:(215)233-7000
TYPE OF SUBMISSION: Commercial Pharmaceutical
DATE OF SUBMISSION: June 15, 1998 **CDER:** June 15, 1998
DATE OF REVIEW: March 15, 1999
REVIEWER: Rosemarie Neuner, MD, MPH
CSO: Mr. Kerry Rothschild, JD

Introduction

Ibuprofen is a propionic acid derivative that belongs to the nonsteroidal anti-inflammatory class of drugs (NSAIDs). A suspension formulation of ibuprofen (100 mg/5 mL) has been marketed in the United States since 1989 by McNeil Consumer Products for use in children (age 6 months and older) as a prescription drug, under the trade names, Pedia-Profen and Children's MOTRIN® Suspension. On June 10, 1995 Children's MOTRIN® (ibuprofen oral suspension) Drops, 50 mg/1.25 mL was approved by the U.S. Food and Drug Administration for marketing as an over-the-counter (OTC) drug product for the temporary relief of fever and pain in children 2-3 years of age. In June 1998, the sponsor of this product, McNeil Consumer Products, submitted a request to the agency for a pediatric exclusivity claim which was subsequently granted. The sponsor has now submitted this efficacy supplement for agency review in which they request the lowering of the currently approved group age range from two to three years of age down to two months of age for this product.

In support of this change in the product's dosing age range, the sponsor has submitted the results of a new subgroup analysis of data generated from 27,000 children less than 2 years of age who participated in the actual use drug -safety trial, the Boston University Fever Study, which evaluated the safety profile of Children's MOTRIN® as an antipyretic agent. (Note: This was the pivotal safety study that supported the approval for the sponsor's NDA 20-516 Children's MOTRIN® Ibuprofen Oral Suspension 100 mg/5 mL in 1995. It also served as the supportive safety study in the approval of the sponsor's other NDA 20-603 Children's MOTRIN® Ibuprofen Drops 50 mg/1.25 mL in 1996.) In addition, the sponsor has included the results of 21 clinical trials where children 2 years old and younger participated as study subjects, in addition to 4 published pharmacokinetic (PK) studies involving children ages 2 months to 2 years. The results from these PK studies are discussed in the PK section of this SNDA review by Dr. E. Dennis Bashaw, FDA Division of Pharmacokinetics (HFD-880).

Since prescription ibuprofen is currently approved for use in infants age 6 months and older, the major regulatory issue to be answered by this application is it

safe for OTC ibuprofen to be used in the pediatric age group 2 months and older at the doses proposed by the sponsor of this supplement. This review will therefore concentrate on the drug's safety profile in this targeted age group.

Efficacy

In support of ibuprofen's efficacy in the targeted pediatric age group of 2 months to 2 years, the sponsor performed an extensive search of the worldwide literature. This search yielded 23 articles and 6 abstracts which described the results of 21 randomized, controlled antipyretic (16) and analgesia (5) trials which evaluated ibuprofen in children ages less than 2 years of age. A complete listing of these articles and abstracts, and their trial summaries written by the sponsor can be found in the following sponsor's tables, Tables 8-10 and 8-12, in Attachment I.

A total of 2,032 febrile children between the ages of 2 months to 13 years participated in the antipyretic studies. (See the following sponsor's table, Table 8-10, found in Appendix I.) Of these 16 studies, 2 were placebo-controlled trials. The other 14 studies compared ibuprofen to active controls such as acetaminophen or aspirin. Five (5) out of the 16 studies were single-dose studies while the remaining 11 trials were multi-dose studies of ibuprofen. These 16 trials tested doses of ibuprofen in the range of 0.5 mg/kg to 10 mg/kg. All 16 studies showed that ibuprofen at the doses tested, with the exception of the lowest dosing range, was an efficacicus antipyretic agent in the populations tested. (Refer to Table 8-10 found in Attachment I at the end of this review.)

A total of 504 children between the ages of 6 months to 14 years participated in the 4 postoperative and 1 otitis media analgesic studies. (See the following sponsor's table, Table 8-12, located in Attachment I.) Two out of the 5 trials were placebo-controlled studies, 2 were placebo- and active- controlled studies, and 1 study evaluated ibuprofen as a single-agent with codeine used for rescue pain. Three of the 5 studies evaluated multi-doses while 2 were single-dose trials. The dose range of ibuprofen used in these analgesia studies ranged from 5 mg/kg to 13 mg/kg. All 5 studies showed that ibuprofen at the doses tested was comparable to acetaminophen or more efficacious than placebo in the control of pain in the patients studied.

The sponsor created the following 2 tables, Sponsor's Tables 1 and 2, below, to show how many studies in this collection included study subjects from the targeted pediatric age group. With the exception of one randomized, double-blind, actively controlled antipyretic trial which compared ibuprofen 7.5 mg/kg to acetaminophen 10 mg/kg in 154 children aged 6-months to 5-years, all of the remaining studies used descriptive statistics (i.e., mean, standard deviation, and range) in discussing the age of the subjects who participated in the studies. Thus, it is impossible to know how many children less than 2 years of age actually participated in these studies.

Sponsor's Table 1 - Age of the Youngest Patient Included in Published Efficacy Antipyretic Trials

Age	Number of Studies	Literature Reference [one study]
**2-3 months**	6	[8] [19,49] [20] [25] [26] [27]
**4-11 months**	9	[6] [7] [10,13] [11,17] [12] [15,22] [14,16,24] [18,21] [23]
**12-23 months**	1	[9]

Sponsor's Table 2 - Age of the Youngest Patient Included in Published Efficacy Analgesic Trials

Age	Number of Studies	Literature Reference [one study]
**2-3 months**	0	None
**4-11 months**	1	[30]
**12-23 months**	4	[28] [29,33] [31] [32]

Since these studies are not proprietary studies, and their actual case reports and data sets were not included in this submission for review, they can only be considered supportive of the already established efficacy profile of ibuprofen in children ≤ 2 years old. As previously discussed, ibuprofen as a prescription drug is considered to be efficacious in the sponsor's requested pediatric target age group for this submission. Thus, these studies are being included in this review at this time for completeness and reference.

Medical Reviewer's Comments: All 21 studies showed that ibuprofen was an efficacious agent for the indications studied when compared to placebo and other recognized antipyretic and analgesic agents. Thus, these studies can be used in support of ibuprofen's already recognized effectiveness as an antipyretic and analgesic agent in the sponsor's requested targeted age group.

Safety

As discussed in the preceding introduction, the focus of this review is to determine whether ibuprofen is safe to be used as an OTC agent in the sponsor's requested targeted pediatric age group of 2-months to 2-years. In support of this product's safety profile the sponsor has submitted for review the following safety data for children less than 2-years of age:

1. The Boston University Fever Study subgroup analysis of children less than 2-years of age.
2. McNeil CPC controlled clinical trial data on subjects ≤ 2-years of age enrolled on or after November 17, 1993 and treated with ibuprofen.
3. McNeil CPC Spontaneous Reporting System for McNeil CPC ibuprofen products in children ≤ 2-years of age for the time period November 17, 1993 through October 2, 1997, including serious reports in the published literature.
4. FDA Spontaneous Reporting System for all ibuprofen products in children ≤ 2-years of age for the time period November 1, 1993 through August 25, 1997. (Note: Adverse events reported through McNeil Spontaneous Reporting System are not included here.)
5. Published randomized controlled clinical trials and human pharmacokinetic studies of ibuprofen products for the years 1966 through October 1997 that reported including children ≤ 2-years of age.
6. AAPCC TESS ibuprofen data from the years 1994 through 1996 for children ≤ 2-years of age. (The 1997 report was not yet available.)

The sponsor has compiled the following summary table, Table 8-13, which outlines the total number of serious adverse events that have been reported to have occurred in children < 2-years of age since November 1993 from the above submitted safety data base. (See sponsor's table, Table 8-13, below.)

The cornerstone of this safety data base for children (see sponsor's table, Table 8-13, below) is generated from the actual use safety study, the Boston Fever Study. Although this study was reviewed by the agency in support of a regulatory action for NDA 20-516 Children's MOTRIN® Ibuprofen Oral Suspension 100 mg/5 mL in 1995, the sponsor has submitted for review a new subcohort analysis of the 27,065 children < 2 years of age who participated in this study which compares the incidence of adverse events that occurred during the trial in this group to that of subjects age ≥ 2 years. This study will be discussed first followed by reviews of the other safety data as listed above.

Table 8-13. Summary of Ibuprofen Safety Data For Children Less Than Two Years of Age (data since November 1993, except where noted otherwise)

**Boston University Fever Study (children < two years of age)**	(Total Patients)	27,065
Total Ibuprofen Exposures		17,938
Hospitalizations for events of primary interest		3
Other hospitalizations (excluding deaths)		258
Deaths		0
**McNeil CPC Controlled Clinical Studies (children < two years of age)**		
Antipyretic Study	(Total Patients)	1
Total Ibuprofen Exposures[1]		1
Reports of AEs with serious outcomes (excluding deaths)[2]		0
Deaths		0
**Published Randomized Controlled Studies (inclusive of, but not limited to, children < two years of age[4]) (first study published in 1976)**		
Antipyretic Studies	(Total Patients)	2032
Total Ibuprofen Exposures		974
Serious AEs (excluding deaths)[5]		0
Deaths		0
Analgesic Studies	(Total Patients)	504
Total Ibuprofen Exposures		213
Serious AEs (excluding deaths)[5]		0
Deaths		0
Pharmacokinetic/Pharmacodynamic Studies	(Total Patients)	340
Total Ibuprofen Exposures		194
Serious AEs (excluding deaths)[5]		0
Deaths		0

1 One child less than two years of age was unintentionally enrolled in a McNeil CPC controlled trial of ibuprofen for which enrollment was planned for children two to 11 years of age (Protocol No. 95-516).

2 A serious outcome is defined as an adverse event that is life threatening (immediate risk of death from the reaction), requires inpatient hospitalization, prolonged hospitalization, or is permanently or severely disabling. Outcomes of death, congenital anomaly, or cancer are also considered serious.

3 Generally, a moderate outcome involves a patient who exhibits signs or symptoms as a result of the exposure which are pronounced, prolonged, or of a systemic nature; usually some form of treatment is required. Symptoms are not life-threatening and the patient has no residual disability or disfigurement. A major outcome generally involves a patient who exhibits signs or symptoms as a result of the exposure which are life-threatening or result in significant residual disability or disfigurement.

4 While overall safety information was reported, such information for children less than two years of age was not specified.

5 Serious as defined by the investigator.

1. The Boston Fever Study Subcohort Analysis of Children Less Than 2 Years of Age.

This was a 4-week, multicenter, double-blind, randomized, acetaminophen-controlled antipyretic study conducted by the Slone Epidemiology Unit of Boston University in office-based pediatric population from the continental United States. The study's objective was to assess the risk of serious adverse events such as gastrointestinal bleeding, acute renal failure, anaphylaxis and Reye syndrome associated with the use of ibuprofen in febrile children. Children between 6 months to 12 years of age weighing between 7-50 kg were recruited after presenting for a pediatric evaluation of an acute febrile illness to any one of the 1,735 pediatricians or family practitioners who participated in the trial. In order to be eligible for study entry, the children had to be able to take the study medication by mouth, and have a parent/guardian able to administer the study medication while observing and caring for them. Children who were dehydrated, unable to take medication by mouth, or with histories of hypersensitivity to acetaminophen or NSAIDS, renal or hepatic diseases, bleeding disorders, anemia, neoplasia, endocrine or metabolic problems, or peptic ulcer disease were ineligible for study entry. A total of 84,192 patients were entered into the trial out of which 83,915 patients were randomized and received 1 of the following 3 treatments: 5 mg/kg ibuprofen, 10 mg/kg ibuprofen, or 12 mg/kg acetaminophen.

Out of the total of 83,915 children entered into the study, 27,065 were < 2 years and 56,850 were ≥ 2 years of age. Demographically, the 2 age groups on comparison as well as the 3 randomized treatment groups were very similar in make up as shown in the following 2 tables, Sponsor's Tables 3 and 4, below.

Sponsor's Table 3 - Demographic Characteristics of All Participants

	Age	
**Characteristic**	**< 2 years (n =27,065)**	**≥ 2 years (n = 56,850)**
**Median Age (Months)**	13	59
**Median Weight (kg)**	10	18
**Sex, %Male**<br>**% Female**	54<br>46	50<br>50
**Race, %White**<br>**% African-American**<br>**%Hispanic**	81<br>7.2<br>7.2	82<br>7.3<br>6.6

Sponsor's Table 4 - Demographic Characteristics of 27,065 Participants ≤ 2 Years Old According to Treatment Group

Characteristic	Acetaminophen	Ibuprofen (5 mg/kg)	Ibuprofen (10 mg/kg)
Total Number	9,127	9,159	8,779
Median Age (Months)	14	13	13
Median Weight (kg)	10	10	10
Sex, % Male	54	54	55
Race, % White	82	81	80
% African-American	7.3	6.8	7.4
% Hispanic	6.7	7.2	7.7

Three-hundred nineteen (319) children (1.1%) ≤ 6 months of age were entered into the study despite an entry age requirement of being at least 6 months or older. Table 5 below lists the numbers of infants ≤ 6 months who participated in the study. (Note: In the official study report the sponsor states that because age was not routinely confirmed, children ≤ 6 months of age were only included in the analysis of the study data if their reported weight was between the 5th and 95th percentile for month of reported age.).

Table 5 - Age Distribution For 319 Children Younger than 6-Months of the 27,065 Participants ≤ 2 Years Old at Enrollment

Age in Months	Number	Percent
1	4	0.015%
2	13	0.048%
3	27	0.010%
4	76	0.281%
5	199	0.735%

The 2 age groups differed in the reported causes of their fevers as shown in Sponsor's Table 6. Although upper respiratory tract infection was the most commonly reported cause of fever for both age groups, in children < 2 years of age, otitis media was more common ($p<0.001$) as compared to children ≥ 2 years of age, who were more commonly afflicted with pharyngitis and lower respiratory tract infections ($p<0.001$ for

both comparisons).

Sponsor's Table 6 - Cause of Fever Among All Participants

Illness (%)	Age: < 2 years (n =27,065)	Age: ≥ 2 years (n = 56,850)
Upper Respiratory Infection	43	42
Otitis Media	48[1]	27
Pharyngitis	19	40[2]
Lower Respiratory Infection	6.3	8.8[3]
Gastrointestinal Infection	3.0	3.2

[1]Statistically significant difference at ($p<0.001$).
[2]Statistically significant difference at ($p<0.001$).
[3]Statistically significant difference at ($p<0.001$).

Sponsor's Table 7 shown immediately below demonstrates that there were no differences in the causes of fever in the 27,065 participants ≤ 2 years of age when examined by randomized antipyretic treatment group.

Sponsor's Table 7 - Cause of Fever Among 27,065 Participants ≤ 2 Years Old According to Treatment Group

Illness (%)	Acetaminophen	Ibuprofen (5 mg/kg)	Ibuprofen (10 mg/kg)
Upper Respiratory Infection	43	43	43
Otitis Media	48	48	48
Pharyngitis	20	19	20
Lower Respiratory Infection	6.5	6.2	6.2
Gastrointestinal Infection	3.0	3.3	2.8

The following 2 tables, Sponsor's Tables 8 and 9, show by age and randomized treatment group the numbers and percentages of children who were randomized, but did not receive study medications. The tables also show that the median number of doses and the median duration of treatment by those who did receive medications was very similar for the subcohort and the original cohort groups, as well as all 3 treatment groups ≤ 2 years of age.

Sponsor's Table 8 - Study Medication Use Among All Participants

Exposure	Age: < 2 years (n =27,065)	Age: ≥ 2 years (n = 56,850)
Treated, %	96.1	95.1
Not Treated, %	3.9	4.9
Doses Received (Median)	6-10	6-10
Duration in Days (Median)	3	3

Sponsor's Table 9 - Study Medication Use Among 27,065 Participants ≤ 2 Years Old According to Treatment Group

Exposure	Acetaminophen	Ibuprofen (5 mg/kg)	Ibuprofen (10 mg/kg)
Treated, %	96.1	96.1	96.0
Not Treated, %	3.9	3.9	4.0
Doses Received (Median)	6-10	6-10	6-10
Duration in Days (Median)	3	3	3
Dose (mg/kg) (Median)	12	4.8	9.6

Study Outcomes:

Although no deaths were reported to have occurred during the duration of the study, 2 children did die during the follow-up period. Both deaths were unrelated to the study medications. The first case involved a 15-month-old black male randomized to the acetaminophen treatment group who died as a result of injuries sustained in a motor vehicle accident. The second case involved an 11-year-old male randomized to ibuprofen 5 mg/kg who died due to complications of meningitis.

The original objective of this study was to assess the risk associated with the use of ibuprofen in febrile children for the occurrence of serious adverse events. The objective of the subcohort analysis was to describe the risk of serious adverse clinical events following the use of ibuprofen in a study subcohort of children < 2 years of age. The original analysis of the entire study cohort found that only 795 (1%) participants out of the 83,915 randomized to receive study medications were hospitalized for any reason during the 4 weeks following study entry. In the subcohort analysis, 385 out of the 27,065 children < 2 years of age and 410 out of 56,850 children ≥ 2 years of age were hospitalized for any reason. (See Sponsor's Table 10, below.)

As part of the statistical analysis of this new subcohort examination, absolute risk and relative risk for the development of serious outcomes were designated to be calculated for comparison purposes by both age and treatment groups for "any" as well as for specifically predesignated adverse events that are of a safety concern in pediatric populations exposed to ibuprofen (i.e., GI bleeding, acute renal failure, anaphylaxis, or Reye Syndrome.) In the < 2 years of age subcohort, the absolute risk for hospitalization due to any reason was found to be 1.4% (95% confidence interval, 1.3-1.6%) vs 0.72% (95% CI, 0.65-0.79%) for children ≥ 2 years of age. (Refer to Sponsor's Table 10 below.) The relative risk for hospitalization due to any reason in the < 2 years of age subcohort as compared to the subcohort ≥ 2 years was found to be 2.0 (95% CI, 1.7-2.3). (See Sponsor's Table 10.)

Sponsor's Table 10 - Risk of Hospitalization for Any Reason According to Age

Age	Total Number	No.Hospitalized	Absolute Risk (95% CI[1])	Relative Risk[2] (95% CI)
<2 yrs.	27,065	385	1.4% (1.3-1.6%)	2.0 (1.7-2.3)
≥2 yrs.	56,850	410	0.72% (0.65-0.79%)	1.0 (--)

[1]Confidence interval.
[2]Risk of hospitalization among children < 2 years of age compared to the risk of hospitalization among children ≥ 2 years of age.
[3]Reference category.

Only 2 out of the 319 infants < 6 months of age who were included in the study were hospitalized. The first case involved an infant hospitalized for the treatment of a viral infection who had been assigned to ibuprofen 5 mg/kg. The other case involved an infant hospitalized with pneumonia who had been assigned to the ibuprofen 10 mg/kg treatment group.

As part of the new "sub" subcohort analysis, the absolute risk of hospitalization for any reason for the 319 infants < 6 months old regardless of antipyretic treatment was 0.63% (95% CI, 0.08-2.2%). When compared to the risk of hospitalization in children ≥ 6 months of age, no significant difference was shown (p=0.8) between these 2 age groups. No significant difference (p=0.5) was also found when comparing the risk of hospitalization for any reason according to assigned antipyretic treatment in infants < 6 months of age.

The following table, Sponsor's Table 11, shows that when comparing the risk for hospitalization for any reason by treatment group assignment according to age, children < 2 years of age treated with ibuprofen (relative risk: 2.1 [95% CI, 1.8-2.5]) and acetaminophen (relative risk - 1.7 [95% CI, 1.8-2.5]) were at a significantly higher risk than children ≥ 2 years old (ibuprofen - relative risk: 1.0 [95% CI]; acetaminophen - relative risk - 1.0 [95%, CI]). (Refer to Sponsor's Table 11 below.) No increase in the risk for hospitalization was noted on comparison of within age groups according to treatment as shown in the next table, Sponsor's Table 12, as shown below. (See the

following table, Sponsor's Table 12.)

Sponsor's Table 11 - Risk of Hospitalization for Any Reason According to Antipyretic Assignment and Age

Antipyretic	Age	Total Number	No. Hospitalized	Absolute Risk/100,000 (95% CI[1])	Rel. Risk[2] 95% CI
**Ibuprofen**	<2 yrs.	17,938	261	1.5% (1.3-1.6%)	2.1 (1.8-2.5)
	≥2yrs.	37,847	262	0.69% (0.61-0.78%)	1.0[3] (---)
**Acetaminophen**	<2 yrs.	9,127	124	1.4% (1.1-1.6%)	1.7 (1.4-2.2)
	≥2yrs.	19,003	148	0.78 (0.66-0.91%)	1.0[3] (---)

[1]Confidence Interval.
[2]Risk of hospitalization among children < 2 years of age compared to the risk of hospitalization with among children randomized to ≥ 2 years of age.
[3]Reference category.

Sponsor's Table 12 - Risk of Hospitalization for Any Reason According to Age and Antipyretic Assignment

Age	Antipyretic	Total Number	Number Hospitalized	Absolute Risk (95% CI[1])	Relative Risk[2] (95% CI)
**<2 years**	Ibuprofen	17,938	261	1.5% (1.3-1.6%)	1.1 (0.87-1.3)
	Acetaminophen	9,127	124	1.4% (1.1-1.6%)	1.0[3] (---)
**≥2 years**	Ibuprofen	37,847	262	0.69% (0.61-0.78%)	0.89 (0.73-1.1)
	Acetaminophen	19,003	148	0.78 (0.66-0.91%)	1.0[3] (---)

[1]Confidence Interval.
[2]Risk of hospitalization among children randomized to ibuprofen compared to the risk of hospitalization among children randomized to acetaminophen.
[3]Reference category.

As stated earlier, one of the original aims of the Boston Fever Study was to assess the risk for the occurrence of GI bleeding, acute renal failure, anaphylaxis and Reye Syndrome in the pediatric population studied. In the original cohort of 83,915 patients that were entered into the study, there were only 4 reported cases of GI bleeding, and no cases of acute renal failure, anaphylaxis, or Reye Syndrome. Sponsor's Table 13 (see below) shows the distribution and the absolute risk by age group for a hospitalization due to acute GI bleeding in the subcohort analysis. In children < 2 years of age, this risk was found to be 11 per 100,000 (95% CI, 2.2 to 32 per 100,000). Since these numbers were so low, there was insufficient data to show a significant difference (Fisher's exact test, p=0.1) when compared with the risk for acute GI bleeding in children ≥ 2 years of age.

Sponsor's Table 13 - Risk of Hospitalization With Acute Gastrointestinal (GI) Bleeding According to Age

Age	Total Number	No.Hospitalized	Absolute Risk per 100,000	95% CI[1]
**<2 years**	27,065	3	11	2.2-32
**≥2 years**	56,850	1	1.8	0.05-9.8

[1]Confidence Interval

As seen in Sponsor's Table 14 (below), all of the GI bleeds occurred in children treated with ibuprofen. Although the highest absolute risk of hospitalization due to an acute GI bleed was found to be associated with children < 2 years of age treated with ibuprofen (17 per 100,000 [95% CI, 3.5-49 per 100,000]), the sponsor reported the risk for the two ibuprofen treatment groups within that age group was similar. However, it was not found to be significantly increased (p=0.6) when compared to the risk associated with children < 2 years of age who were treated with acetaminophen (0 per 9,127 [95% CI, 0-33 per 100,000]). (Refer to Sponsor's Table 14.) In children ≥ 2 years of age, the risk of a hospitalization due to acute GI bleeding in the ibuprofen treated group was 2.6 per 100,000 (95% CI, 0.05-15 per 100,000), and in the acetaminophen treated group it was 0 per 19,003 (95% CI, 0-16 per 100,000). On comparison of the 2 age groups, the risk for hospitalization due to an ibuprofen-induced acute GI bleed was not found to be significantly different (p=0.1).

Sponsor's Table 14 - Risk of Hospitalization with Acute GI bleeding According to Age and Antipyretic

Age	Antipyretic	Total Number	Number Hospitalized	Absolute Risk per 100,000	95% CI
<2 yrs.	Ibuprofen<br>Acetaminophen	17,938<br>9,127	3<br>0	17<br>--	3.5-49<br>0-33
≥2 yrs.	Ibuprofen<br>Acetaminophen	37,847<br>19,003	1<br>0	2.6<br>--	0.05-15<br>0-16

None of the 3 cases of acute GI bleed that occurred in the subcohort study population of < 2 years of age died. The first case (Subject ID 78468989) occurred in a 19-month-old male with a history of Hirschsprung's disease, status post colostomy and Swenson pull-through, and enterocolitis who was randomized to the ibuprofen 50 mg/5 mL treatment group when he presented with a fever due to otitis media. In addition, he also received a course of an unknown antibiotic. This subject received 3 doses of ibuprofen over the next 2-days. On the third day he was hospitalized for evaluation of abdominal pain and vomiting. Records state that his vomitus appeared to look like coffee grounds, and his stool was guaiac positive. He was treated with enemas and stool softeners for a possible bowel obstruction, and improved without further recurrence of GI bleeding during the 9 months of post-study follow up.

The second case (Subject ID 43135762) of acute GI bleed occurred in a 19-month-old male randomized to the ibuprofen 100 mg/5 mL treatment group who hospitalized the day after receiving just 1 dose of the study medication due to guaiac positive diarrhea associated with persistent vomiting. His stool assay was positive for rotavirus antigen. He improved after treatment with IV fluids, antibiotics, and acetaminophen without further episodes of bleeding during the 20 months of post-study follow up.

The last case (Subject ID 85496241) of acute GI bleeding occurred in a 8-month-old female randomized to the ibuprofen 100 mg/5 mL treatment group who had a fever due to a persistent case of otitis media which was treated with Augmentin. She was admitted on the third study day, 48 hours after receiving 2 doses of the study medication over a 24-hour period for evaluation of hematochezia and guaiac positive stools associated with dehydration, vomiting and otitis media. The subject improved with IV hydration and antibiotics and the treating physician attributed the hematochezia to the study medication. There were no reports of the hematochezia recurring during the 2 week post-study follow up.

Although there were no reported cases of acute renal failure, anaphylaxis or Reye syndrome which occurred during this study, the sponsor did calculate the observed risk for both the original study cohort population as well as that of the new subcohort analysis. Since there were no reported cases of these 3 specific adverse

events during the study, only the upper-bound of the 95% confidence interval (CI)could be calculated. In children < 2 years of age, the upper bound of the 95% CI for the risk of hospitalization due to acute renal failure, anaphylaxis, or Reye Syndrome was found regardless of the treatment group was 11 per 100,000; in children ≥ 2 years of age the upper bound for these events was 5.1 per 100,000. (Refer to Sponsor's Table 13.) In children < 2 years of age, the upper bound of the 95% CI for the risk of hospitalization due to these events treated with acetaminophen was found to be 0 per 9,127(95% CI, 0-33 per 100,000); in children < 2 years of age treated with ibuprofen the upper bound for these events was 0 per 17,938 (95% CI, 0-17 per 100,000). (Refer to Sponsor's Table 14.) In infants < 6 months of age, the observed risk of hospitalization for each of the above specific adverse events regardless treatment was 0 per 319 (95% CI, 0-0.94); among infants who received treatment with acetaminophen the observed risk was 0 per 112 (95% CI, 0 to 2.7%); among infants who received treatment with ibuprofen the observed risk was 0 per 207 (95% CI, 0 to 1.5%). (Note: The differences noted in the upper bound of the 95% CI for the infant population is due to its small sample size.)

In view of the fact that there were no cases of acute renal failure which occurred during this trial, the sponsor decided to look at changes in subjects' serum creatinine levels as another means of possibly determining the nephrotoxicity of ibuprofen in the pediatric population. Since the original protocol did not require the measurement and collection of entry and exit serum creatinines, they did a post hoc analysis from lab data collected from 222 (28%) out of the 795 children who were hospitalized while participating in the study. (Note: Only serum creatinines obtained within the first 24-hours of admission were used in this analysis.) The mean creatinine level on admission was 0.48 mg/dL, and 9% of them were higher than 0.7 mg/dL which is the upper limit of normal for children. No significant difference in mean serum creatinine levels was noted when compared by treatment group. Only 112 (29%) out of the 385 children < 2 years of age who were admitted during this study had serum creatinine levels available for analysis. The following table, Sponsor's Table 15 shown below, lists the distribution, mean and range for the serum creatinines collected for data analysis in this age group. (See Sponsor's Table 15.) On cross-treatment group comparison, the difference in mean serum creatinine between the acetaminophen group (0.34 mg/dL) and the ibuprofen treatment group (0.42 mg/dL) was found to be statistically significant (p=0.03) via calculation of an unpaired student's t-test, but when analysis of covariance is used to calculate the p-value taking into account subjects' ages, weight, sex and dehydration, no significant difference was found. Comparison of the prevalence of serum creatinines > 0.07 mg/dL in the acetaminophen and ibuprofen treatment groups, was not found to be significantly different (p=0.32). (See Sponsor's Table 15 below.) (Note: The sponsor reports that although they repeated this analysis with lower thresholds set for an "elevated" serum creatinine, the numbers of cases increased in both treatment groups but the difference was still not statistically significant. Although this data was not included in the submission for review, it needs to be mentioned to document the scope of the sponsor's post hoc analysis.)

Sponsor's Table 15 - Serum Creatinine Among Hospitalized Children < 2 Years Old

	Acetaminophen	Ibuprofen
**Total Number**	29	83
**Serum Creatinine (mg/dL)**<br>**Mean**<br>**(SEM)**<br>**Range**	0.34<br>(0.025)	0.42<br>(0.023)
**Serum Creatinine >0.7 mg/dL**<br>**Number**<br>**(%)**	0<br>(0)	5<br>(6)

The following table, Sponsor's Table 16, lists the mean serum creatinines by treatment group for the subcohort of children < 2 years of age. The sponsor states that they did not do a subanalysis of mean serum creatinines in the subgroup infant population < 6 months of age because too few of these subjects were hospitalized.

Sponsor's Table 16 - Mean Serum Creatinine Among Hospitalized Children < 2 Years Old By Age and Treatment Group

Age	Mean Creatinine	(No.)	Mean Creatinine	(No.)	Mean Creatinine	(No.)
**All**	0.34	(29)	0.43	(46)	0.40	(73)
**12-23 mos.**	0.37.	(17)	0.44	(25)	0.43	(21)
**<12 mos.**	0.32	(12)	0.43	(21)	0.36	(16)

The sponsor also looked at the risk for hospitalizations associated with other adverse events or conditions that may be of potential risk in this younger pediatric age group. They looked at asthma, bronchiolitis, and vomiting/gastritis since these occurred in at least 5 or more subjects in the subcohort population. Sponsor's Table 17, below, shows that there were 32 children < 2 years of age and 36 children ≥ 2 years of age who were hospitalized due to asthma while participating in the trial. The relative risk for hospitalization with asthma in children < 2 years of age was found to be 1.9 (95% CI 1.2 to 3.0) when compared to that in children ≥ 2 years of age.

Sponsor's Table 17 - Risk of Hospitalization with Asthma According to Age

Age	Total Number	No.Hospitalized	Absolute Risk/100,000 (95%CI)	Relative Risk[2] (95% CI)
<2 yrs.	27,065	32	120 (81-70)	1.9 (1.2-3.0)
≥2 yrs.	56,850	410	63 (44-88)	1.0[3] (--)

[1]Confidence interval.
[2]Risk of hospitalization with asthma among children < 2 years of age compared to the risk of hospitalization with asthma among children ≥ 2 years of age.
[3]Reference category.

The following table, Sponsor's Table 18 below, lists in a table the associated absolute and relative risks for the 2 age groups by treatment for hospitalization with asthma. This table shows that regardless of the antipyretic treatment, the risk of hospitalization is inversely related to the child's age.

Sponsor's Table 18 - Risk of Hospitalization with Asthma According to Antipyretic Assignment and Age

Age	Antipyretic	Total Number	Number Hospitalized	Absolute Risk/100,000 (95% CI[1])	Relative Risk[2] 95% CI
<2 yrs.	Ibuprofen	17,938	20	110 (68-170)	1.8 (1.0-3.2)
	Acetaminophen	9,127	24	63 (41-94)	1.0[3] (---)
≥2 yrs.	Ibuprofen	37,847	12	130 (70-230)	2.0 (0.9-4.6)
	Acetaminophen	19,003	12	63 (33-110)	1.0[3] (---)

[1]Confidence Interval.
[2]Risk of hospitalization with asthma among children randomized to ibuprofen compared to the risk of hospitalization with asthma among children randomized to acetaminophen.
[3]Reference category.

Sponsor's Table 19 below, shows the distribution of children hospitalized by age and treatment group for the risk of hospitalization due to asthma. The data in this table demonstrates that treatment with either antipyretic agent was not associated with the risk of hospitalization in either age group. (Refer to Sponsor's Table 19 shown below.)

Sponsor's Table 19 - Risk of Hospitalization with Asthma According to Age and Antipyretic Assignment.

Age	Antipyretic	Total Number	Number Hospitalized	Absolute Risk/100,000 (95% CI[1])	Relative Risk[2] 95% CI
<2 years	Ibuprofen	17,938	20	110 (68-170)	0.9 (0.4-1.7)
	Acetaminophen	9,127	12	130 (70-230)	1.0[3] (---)
≥2 years	Ibuprofen	37,847	24	63 (41-94)	1.0 (0.5-2.0)
	Acetaminophen	19,003	12	63 (33-110)	1.0[3] (---)

[1]Confidence Interval.
[2]Risk of hospitalization with asthma among children randomized to ibuprofen compared to the risk of hospitalization with asthma among children randomized to acetaminophen.
[3]Reference category.

Since it can be difficult to discern between asthma and bronchiolitis in very young children, the sponsor looked at the 37 hospitalized cases of bronchiolitis which occurred during the study. The following 2 tables, Sponsor's Tables 20 and 21, show the study data describing the risk associated with hospitalizations due to bronchiolitis in both subcohort age groups by age as well as treatment group.

Sponsor's Table 20 - Risk of Hospitalization With Bronchiolitis According to Age

Age	Total Number	No.Hospitalized	Absolute Risk per 100,000	95% CI[1]
<2 years	27,065	33	120	84-170
≥2 years	56,850	4	7	2-18

[1]Confidence Interval

Sponsor's Table 21, below, shows that on comparison of the 2 treatment groups, the risk for hospitalization due to bronchiolitis did not vary.

Sponsor's Table 21 - Risk of Hospitalization with Bronchiolitis Among Participants <2 Years of Age According to Antipyretic Assignment

Antipyretic	Total Number	Number Hospitalized	Absolute Risk/100,000 (95% CI[1])	Relative Risk[2] (95% CI)
Ibuprofen	17,938<br>9,127	21	120<br>(72-180)	0.9<br>(0.4-1.8)
Acetaminophen	9,127	21	130<br>(70-230)	1.0[3]<br>(---)

[1]Confidence Interval.
[2]Risk of hospitalization with bronchiolitis among children randomized to ibuprofen compared to the risk of hospitalization with bronchiolitis among children randomized to acetaminophen.
[3]Reference category.

The sponsor also looked at the number of cases who were hospitalized due to vomiting/gastritis during the study. Sponsor's Table 22, below, shows the numbers of children and the associated risks for hospitalization due to vomiting/gastritis for both subcohort populations. On comparison between age groups, the risk for hospitalization due to vomiting/gastritis did not vary.

Sponsor's Table 22 - Risk of Hospitalization With Vomiting/Gastritis According to Age

Age	Total Number	No.Hospitalized	Absolute Risk/100,000 (95% CI[1])	Relative Risk[2] (95% CI)
**<2 years**	27,065	9	33<br>(15-63)	1.1<br>(0.5-2.5)
**≥2 years**	56,850	17	30<br>(17-48)	1.0[3]<br>(---)

[1]Confidence Interval.
[2]Risk of hospitalization with vomiting/gastritis among children randomized to ibuprofen compared to the risk of hospitalization with vomiting/gastritis among children randomized to acetaminophen.
[3]Reference category.

The last table, Sponsor's Table 23, below, demonstrates that the risk for hospitalization due to vomiting/gastritis did not increase with treatment with either acetaminophen or ibuprofen, nor was it shown to vary with age or antipyretic treatment.

Sponsor's Table 23 - Risk of Hospitalization with Vomiting/Gastritis According to Antipyretic Assignment and Age

Antipyretic	Age	Total Number	No. Hospitalized	Absolute Risk/100,000 (95% CI[1])	Rel. Risk 95% CI
**Ibuprofen**	<2 yrs.	17,938	7	39 (16-80)	1.1 (0.5-2.9)
	≥2yrs.	37,847	13	34 (18-59)	1.0[3] (---)
**Acetaminophen**	<2 yrs.	9,127	2	22 (2.6-79)	NA[4]
	≥2yrs.	19,003	4	21 (5.8-54)	1.0[3] (---)

[1]Confidence Interval.
[2]Risk of hospitalization with vomiting/gastritis among children < 2 years of age compared to the risk of hospitalization with vomiting/gastritis among children randomized to ≥ 2 years of age.
[3]Reference category.
[4]Relative risk not calculated because the number hospitalized in at least one group was < 5.

Medical Reviewer's Comments: There are many methodological problems associated with this subcohort analysis of the Boston Fever Study. The original study was unable to accomplish one of its aims which was to assess the risk associated with the use of ibuprofen in a pediatric population for developing GI bleeds, acute renal failure, anaphylaxis and Reye syndrome. It is unclear if this was due to problems failing to measure or capture these adverse events or if the design introduced selection bias based on having health care providers "select" good candidates (i.e., children who were not too sick and had intelligent caretakers.) Since the new subcohort analysis was a post hoc analysis of the original trial data, the validity of its findings are subject to the same issue.

Some of the laboratory data subanalyses performed in this submission did not make good sense to this reviewer such as using the serum creatinines as surrogate markers for more significant problems were not validated.

The original protocol also had an age entry criteria of > 6 months, but the subanalysis reveals that 319 infants ≤ 5 months old were entered into the study. These enrollments constitute trial violations and thus, both the subcohort and "sub-subcohort" infant analysis which draw on this data for support technically should be discounted.

Despite these methodological flaws, the study's size does provide some useful information. Thus, based on the above study data reviewed, and the paucity of adverse events that actually occurred in such a large pediatric population (subcohort population

of n=27,065), it is fairly obvious that ibuprofen at the 2 doses tested is safe to be used in an OTC pediatric population < 2 years of age. The real question posed to this reviewer is at what age is it no longer safe to be used as an OTC product? Unfortunately, there is no answer to that question based on the data submitted in this SNDA. Sponsor's Table 5, demonstrates numerically how few infants between the ages of 2 and 5 months actually participated (as protocol violations no less) in the study (n=319), with the percentage of infants < 6 months of age enrolled in the study comes to only < 1.2% of the total subcohort population. Thus, it is the opinion of this medical reviewer that this study fails to generate sufficient support for a pediatric OTC claim in children < 6 months of age.

2. McNeil CPC controlled clinical trial data on subjects ≤ 2-years of age enrolled on or after November 17, 1993 and treated with ibuprofen.

Since the above listed date, sponsor states in this submission that they have not conducted any clinical trials in children ≤ 2 years of age. One 19-month-old child was inadvertently randomized to the ibuprofen suspension 7.5 mg/kg treatment group of a 2-arm, single-dose, randomized, investigator-blinded antipyresis trial that compared ibuprofen to acetaminophen 12.5 mg/kg. The child reportedly did not experience any adverse effects from this exposure.

Medical Reviewer's Comments: Noted.

3. McNeil CPC Spontaneous Reporting System for McNeil CPC ibuprofen products in children ≤ 2-years of age for the time period November 17, 1993 through October 2, 1997, including serious reports in the published literature.

A search of the sponsor's own CPC Spontaneous Reporting System (SRS) for both serious and nonserious adverse event reports in children ≤ 2 years of age who ingested either the prescription or OTC formulations of Children's Motrin® yielded 9 serious and 305 nonserious reports from health care professionals and consumers. A total of 18 and 361 adverse events were generated by COSTART terminology respectively for serious and nonserious adverse events. Two (2) out of the 9 serious cases resulted in the deaths of the children due to Invasive Group A streptococcal infection post-varicella infection (1) and renal failure (1). The 7 remaining serious cases resulted in the hospitalizations of the children involved due to the following adverse events: drug-induced anaphylaxis (1), dehydration (1), anemia (1), and sepsis syndrome secondary to varicella lesions (4). The sponsor has provided the following summary table, Table 8-40, which describes and lists these 9 serious cases in tabular format in children ≤ 2 years of age. Table 8-41, lists all of the 361 nonserious adverse event reports by body system. The sponsor reported in this submission that out of the original 305 nonserious reports received by them in this age group, 272 reports were associated with their (OTC) Children's Motrin® Suspension formulation, 14 reports were

Table 8-40. **AE Reports with Serious Outcomes in Children Less Than Two Years of Age Received by McNeil CPC from November 17, 1993 through October 2, 1997 for Motrin® Ibuprofen Products, Children's Motrin® Ibuprofen Products, and Unknown Pediatric Ibuprofen Products**

Case No.	Product Form[1]	Mfr. Control No.	Date Received	Age	Sex	AE	COSTART Term	Daily Dose	Duration of Drug	Outcome
1	MOS	0372236A	04/06/95	8 mo	Female	Cellulitis	Cellulitis.	Unknown	Unknown	Hospitalization
						Anemia	Anemia			
2	CMS	0724826A	01/27/97	11 mo	Male	Lips and eyes swelled	Edema face	Unknown	1 dose	Hospitalization
						Increase in number of hives	Urticaria			
						Trouble breathing	Dyspnea			
3	MOS	0867864A[2]	09/23/97	11 mo	Male	Renal failure	Kidney failure	10 mg/kg per dose	Unknown	Hospitalization[2]
4	MOS	0313900A	10/26/94	1 yr	Male	Creatine phosphokinase increased	Creatine phosphokinase increased	100 mg, q4h	6-8 months	Hospitalization
						Convulsion	Convulsion			
						Septic shock	Sepsis			
						Gastrointestinal hemorrhage	Gastrointestinal hemorrhage			
5	MOS	0356256A	02/23/95	1 yr	Female	Dehydration	Dehydration	Unknown	Unknown	Hospitalization
6	MOS	0356314A	02/23/95	1 yr	Male	Cellulitis face	Cellulitis	Unknown	Unknown	Hospitalization
						Bilateral otitis media	Otitis media			
7	MOS	0356366A	02/23/95	1 yr	Female	Infection	Infection	Unknown	Unknown	Hospitalization
8	CMS	0526725A	02/09/96	15 mo	Male	Hemoglobin and hematocrit decreased	Hypochromic anemia	10mg/kg, q8h	8 days	Hospitalization
9	MOS	0356831A	02/23/95	1.5 yr	Male	Sepsis	Sepsis	Unknown	Unknown	Death
						Meningitis	Meningitis			
						Cardiac arrest	Heart arrest			

[1] MOS = Prescription Motrin® ibuprofen suspension, CMS = OTC Children's Motrin® ibuprofen suspension.
[2] *The infant's renal function recovered. The physician reported that the infant died some time later due to unknown complications unrelated to the reported event.*

Table 8-41. **Body System Summary for AE Reports with Nonserious Outcomes For Children Less Than Two Years of Age Received by McNeil CPC from November 17, 1993 through October 2, 1997 for Motrin® Ibuprofen Products, Children's Motrin® Ibuprofen Products, and Unknown Pediatric Ibuprofen Products**

Body System<br>Adverse Event	Number
**Body as a whole**	87
Asthenia	2
Edema face	8
Hypothermia	3
Lab test abnormal	2
Malaise	2
No drug effect	21
Overdose	1
Accidental Overdose	40
Pain	1
Abdominal pain	7
**Cardiovascular system**	2
Tachycardia	1
Peripheral vascular disease	1
**Digestive system**	62
Anorexia	1
Constipation	4
Diarrhea	14
Dyspepsia	3
Dysphagia	2
Eructation	1
Flatulence	3
Hemorrhagic gastritis	1
Glossitis	2
Gastrointestinal hemorrhage	1
Nausea	1
Stomatitis ulcer	1
Abnormal stools	5
Vomiting	23
**Hemic and lymphatic system**	2
Ecchymosis	1
Eosinophilia	1
**Metabolic and nutritive disorder**	2
Peripheral edema	1
Hyperglycemia	1
**Musculoskeletal system**	1
Arthralgia	1
**Nervous system**	119
Confusion	1
Convulsion	1
Dizziness	2
Abnormal dreams	1
Emotional lability	2
Abnormal gait	1
Hallucinations	1
Hyperkinesia	13
Insomnia	30
Nervousness	36
Restlessness	12
Screaming syndrome	6
Somnolence	10
Stupor	1
Tremor	2

Table 8-41. Body System Summary for AE Reports with Nonserious Outcomes For Children Less Than Two Years of Age Received by McNeil CPC from November 17, 1993 through October 2, 1997 for Motrin® Ibuprofen Products, Children's Motrin® Ibuprofen Products, and Unknown Pediatric Ibuprofen Products

Body System<br>Adverse Event	Number
**Respiratory system**	10
Burning of the throat	1
Cough increased	1
Dyspnea	3
Epistaxis	3
Pharyngitis	1
Rhinitis	1
**Skin and appendages**	71
Erythema multiforme	1
Pruritus	4
Rash	46
Skin discolor	1
Sweat	3
Urticaria	16
**Urogenital system**	5
Oliguria	1
Urine abnormality	4
**Total for all body systems**	361

APPEARS THIS WAY
ON ORIGINAL

APPEARS THIS WAY
ON ORIGINAL

for their Children's Motrin® Drops, 1 report was for their Children's Motrin® Chewable tablets, 17 were for their prescription Motrin® Suspension, and 1 was for their prescription Motrin® Drops.

Medical Reviewer's Comments: Review of the narratives of these 9 serious adverse event cases does not reveal any information that could signal any unforeseen adverse event associated with the use of OTC ibuprofen in children ≤ 2 years of age. Cases associated with Invasive Group A Streptococcal infections have been reviewed by the agency's epidemiologists in the past, and no association was found.

4. FDA Spontaneous Reporting System (SRS) for all ibuprofen products in children ≤ 2-years of age for the time period November 1, 1993 through August 25, 1997. (Note: Adverse events reported through McNeil Spontaneous Reporting System are not included here.)

A query of the FDA's SRS database yielded 20 serious adverse event reports in children < 2 years of age which were related to either the use of a prescription or OTC formulation of ibuprofen. Two out of the 20 cases were reports which describe the same fatal overdose case in a 23-month-old female who died due to aspiration pneumonia that were submitted by the sponsor's competitor. (Note: This was a case of an accidental overdose, a further description of which can be found in the following 6b. Overdose Section below.) The sponsor has prepared the following 2 tables, Tables 8-42 and 8-43, which list by COSTART body system terminology all 51 of the adverse events coded for these 19 serious cases (Table 8-42), and a tabular summary of the 19 cases themselves (Table 8-43). Four (4) out of these 19 serious cases resulted in the death of the child due to pulmonary hemorrhage (1), sepsis with cardiac arrest (2), and aspiration pneumonia (1). (Note: The last case is the case that was reported twice to the system.)

The next table, Table 8-44, lists the 145 nonserious adverse events generated from a total of 52 case reports in the FDA's SRS database by COSTART terminology.

Medical Reviewer's Comments: Review of these 19 serious cases does not reveal any information that could signal any unforeseen adverse event associated with the use of OTC-ibuprofen in children ≤ 2 years of age. However, one must keep in mind that these cases occurred in situations where access to the drug was controlled by a health care provider (i.e., via a prescription). Thus, this reviewer is unable to predict if the occurrence of these events will increase in frequency when this product is available to a pediatric population ≤ 2 years of age.

Table 8-42. **Body System Summary for AE Reports with Serious Outcomes in Patients Less Than Two Years of Age. FDA Spontaneous Reporting System from November 1993 through August 1997, for Ibuprofen Products (Excluding AE Reports Received by McNeil CPC Presented in Section 8.6.5.1)**

Body System<br>Adverse event	Number
**Body as a whole**	19
Allergic reaction	1
Congenital anomaly	1
Asthenia	1
Fever	2
Flu syndrome	1
Infection	3
Necrosis	1
No drug effect	1
Overdose	2
Accidental Overdose	2
Perinatal disorder	2
Sepsis	2
**Cardiovascular system**	2
Heart arrest	2
**Digestive system**	5
Diarrhea	1
Hepatitis	1
Nausea and vomiting	1
Stomach ulcer hemorrhage	1
Vomiting	1
**Hemic and lymphatic system**	2
Hemolytic anemia	1
Purpura	1
**Metabolic and nutritive disorder**	4
Creatinine increased	1
Dehydration	1
Edema	1
Hyperkalemia	1
**Musculoskeletal system**	6
Pyogenic arthritis	1
Tendinous contracture	2
Myositis	1
Osteomyelitis	2
**Nervous system**	2
Grand mal convulsion	1
Meningitis	1
**Respiratory system**	2
Lung hemorrhage	1
Aspiration pneumonia	1
**Skin and appendages**	3
Epidermal necrolysis	1
Erythema multiforme	1
Rash	1
**Urogenital system**	5
Bacterial infection	3
Acute kidney failure	3
Total for all body systems	51

Table 8-43. Reports of Adverse Events with Serious Outcomes in Patients Less Than Two Years Old from FDA Spontaneous Reporting System from November 1993 through August 1997, for Ibuprofen Products (Excluding Children's Motrin® Data Presented in Section 8.6.5.1).

Case	Date	Age[1]	Sex[2]	Mfr Control	Manufacturer	Drug	Dosage[3]	Duration	COSTART	Outcome	Concomitant Drugs
1	Jun 94	M01	F	463617463	Upjohn	Motrin	800. Mg	10 Days	Perinatal disorder<br>Stomach ulcer hemorrhage	Hospitalized<br>Required Intervention<br>Life-Threatening<br>Other	
2	Jul 94	M01	F	IBU9410013	Boots	IBU	600. Mg	12 Days	Edema<br>Lung hemorrhage<br>Perinatal disorder<br>Purpura	Died<br>Required Intervention<br>Life-Threatening	
3	Feb 95	M01	M	95001	Wyeth<br>------	Effexor<br>Ibuprofen	37.5 Mg<br>-----	----<br>----	Congenital anomaly	Congenital Anomaly	Amitriptyline<br>Cefadroxil<br>Methadone
4	Aug 94	M08	F	SEPTRA2106	Whitehall<br>BW	Advil<br>Septra	-----<br>5. Ml	----<br>8 Days	Diarrhea<br>Fever<br>No Drug Effect<br>Rash	Hospitalized	Amoxicillin
5	Mar 95	M12	F	895073005A	Parke-Davis<br>Wyeth	Benadryl<br>Ibuprofen	-----<br>-----	----<br>----	Infection	Hospitalized	
6	Apr 95	M12	F	35622	Parke-Davis<br>------	Benadryl<br>Ibuprofen	-----<br>-----	----<br>----	Infection	Hospitalized	
7	Oct 95	M12	M	895289002A	Wyeth	Children's Advil	400. Mg	55 Days	Hemolytic anemia	Hospitalized<br>Life-Threatening<br>Recovered	
8	Mar 95	M13	F	35334	Parke-Davis<br>Wyeth<br>McNeil	Benadryl<br>Children's Advil<br>Pediaprofen	-----<br>75. Mg<br>100. Mg	----<br>2 Days<br>6 Days	Tendinous contracture<br>Bacterial infection<br>Necrosis	Hospitalized	
9	Mar 95	M13	F	895060001A	Parke-Davis<br>Wyeth<br>McNeil	Benadryl<br>Children's Advil<br>Pediaprofen	-----<br>75. Mg<br>100. Mg	----<br>2 Days<br>6 Days	Fever<br>Tendinous contracture<br>Bacterial infection<br>Myositis	Hospitalized<br>Required Intervention<br>Recovered	
10	Mar 95	M14	M	895032011A	------<br>Wyeth	Calamine<br>Children's Advil	-----<br>-----	----<br>----	Infection<br>Osteomyelitis	Hospitalized<br>Required Intervention<br>Recovered	

Table 8-43. Reports of Adverse Events with Serious Outcomes in Patients Less Than Two Years Old from FDA Spontaneous Reporting System from November 1993 through August 1997, for Ibuprofen Products (Excluding Children's Motrin® Data Presented in Section 8.6.5.1).

Case	Date	Age[1]	Sex[2]	Mfr Control	Manufacturer	Drug	Dosage[3]	Duration	COSTART	Outcome	Concomitant Drugs
11	Mar 95	M14	M	34903	Parke-Davis<br>------<br>------	Benadryl<br>Calamine<br>Ibuprofen	-----<br>-----<br>-----	----<br>----<br>----	Pyogenic arthritis<br>Bacterial infection<br>Osteomyelitis	Hospitalized	
12	Oct 95	M16	M	895139003A	Wyeth	Children's Advil	-----	1 Day	Acute kidney failure	Hospitalized	
13	Oct 95	M16	M	895139003A	Whitehall	Advil	-----	1 Day	Dehydration<br>Flu Syndrome<br>Acute kidney failure<br>Nausea and vomiting	Hospitalized	
14	Mar 96	M16	F	--------	------	Ibuprofen	-----	11 Day	Allergic reaction<br>Epidermal necrolysis<br>Erythema Multiforme<br>Hepatitis	Hospitalized<br>Required Intervention<br>Life-Threatening	Ativan<br>Fentanyl<br>Proventil<br>Tylenol
15	Mar 94	M17	M	894061001L	Wyeth	Children's Advil	1. Tp	3 Day	Asthenia<br>Creatinine increased<br>Heart Arrest<br>Sepsis	Died	Zantac
16	Jul 95	M17	M	94055	Whitehall	Advil	27-28 Tb		Accidental Overdose	Hospitalized<br>Required Intervention	
17	Mar 95	M18	M	895073003A	Wyeth<br>McNeil	Ibuprofen<br>Tylenol	-----<br>-----	----<br>----	Heart Arrest<br>Bacterial infection<br>Meningitis<br>Sepsis	Died<br>Hospitalized	
18	May 97	M21	M	515517463	Upjohn	Ibuprofen	8 Gm	----	Grand mal convulsion<br>Hyperkalemia<br>Acute kidney failure<br>Accidental Overdose	Hospitalized<br>Required Intervention<br>Other	
19	Jun 97	M23	F	970170176	Whitehall	Children's Advil	2. Tp	1 dose	Overdose<br>Aspiration pneumonia	Died	Cortef<br>Desmopressin Acet<br>Levoxyl
20	Feb 97	M23	F	897009001S	Whitehall	Advil	2. Tp	1 dose	Overdose<br>Vomiting	Died<br>Other	Cortef<br>Desmopressin Acet<br>Levoxyl

[1] Age in months (preceded by 'M'); [2] M = male, F = female; [3] Tb = tablets, Tp = teaspoon

Table 8-44. **Body System Summary for AE Reports with Non-Serious Outcomes. FDA Spontaneous Reporting System from November 1993 through August 1997, for Ibuprofen Product (Excluding McNeil CPC Data Presented in Section 8.6.5.1) in Patients Less Than Two Years of Age**

Body System<br>Adverse Event	Number
**Body as a whole**	**44**
Abdomen enlarged	2
Allergic reaction	1
Anaphylactoid reaction	1
Congenital anomaly	1
Chills	1
Chills fever	1
Edema face	4
Fever	3
Flu syndrome	1
Hypothermia	1
Infection	1
No drug effect	6
Overdose	1
Accidental Overdose	17
Pain	1
Perinatal disorder	1
Reaction unevaluable	1
**Cardiovascular system**	**4**
Hypotension	1
Vasodilation	3
**Digestive system**	**20**
Anomaly tooth	1
Constipation	1
Diarrhea	3
Dysphagia	1
Flatulence	1
Gastrointestinal hemorrhage	1
Liver function abnormal	2
Abnormal stools	1
Vomiting	9
**Hemic and lymphatic system**	**2**
Cyanosis	1
Leukopenia	1
**Metabolic and nutritive disorder**	**6**
Acidosis	1
Peripheral edema	2
Hypoglycemia	1
Alkaline phosphatase increased	2
**Nervous system**	**38**
Agitation	1
Chronic brain syndrome	1
Coma	1
Convulsion	2
Dizziness	2
Emotional lability	1
Hostility	1
Hyperkinesia	5
Insomnia	6
Manic react	1
Nervousness	6
Screaming syndrome	2

Table 8-44. Body System Summary for AE Reports with Non-Serious Outcomes. FDA Spontaneous Reporting System from November 1993 through August 1997, for Ibuprofen Product (Excluding McNeil CPC Data Presented in Section 8.6.5.1) in Patients Less Than Two Years of Age

Body System<br>Adverse Event	Number
**Nervous system (continued)**	
Somnolence	5
Stupor	1
Tremor	2
Twitch	1
**Respiratory system**	3
Apnea	1
Dyspnea	1
Hypoventilation	1
**Skin and appendages**	27
Alopecia	1
Erythema multiforme	1
Pruritus	1
Rash	15
Maculopapular rash	2
Skin dry	1
Urticaria	6
	1
**Urogenital system**	
Hematuria	1
**Total for all body systems**	145

APPEARS THIS WAY
ON ORIGINAL

APPEARS THIS WAY
ON ORIGINAL

5. Published randomized controlled clinical trials and human pharmacokinetic studies of ibuprofen products for the years 1966 through October 1997 that reported including children ≤ 2-years of age.

An extensive literature search of the worldwide literature by the sponsor yielded a total of 29 articles which discussed the data from 21 single-dose and multi-dose clinical studies with a total combined pediatric population of 3,006 subjects. (Note: More information about these studies can be found in the preceding efficacy section, and in the Sponsor's Tables 8-10 and 8-12, in Attachment I.) No serious adverse events were reported to have occurred in any of these studies. Two studies did not report any safety data and thus are excluded from this safety review. Nine out of the remaining 19 trials did report the occurrence of non-serious adverse events in ibuprofen-treated children which included: nausea, vomiting, diarrhea, rash, hypoglycemia, agitation, febrile seizures, exanthem, insomnia, hypothermia, epistaxis, sweating, GI complaints, discomfort, and hypothermia. Many of these adverse events were not considered by the authors of these published studies to be related to treatment with ibuprofen. Since these trials only used descriptive statistics in discussing their patient populations, it is impossible for this reviewer to determine if any of the above listed adverse events occurred in subjects < 2 years of age based on the data presented.

A total of 340 children between the ages of 3 months to 12 years were enrolled in the 5 pharmacokinetic studies submitted in support of this application. The investigators of these studies did not report the occurrence of any serious or non-serious adverse event during these trials. (Refer to the PK review of this NDA review for more information.)

Medical Reviewer's Comments: This reviewer agrees with the authors of these studies that most of the adverse events reported associated with these trials were probably related to the subjects underlying febrile illnesses (febrile seizures, discomfort, exanthem, nausea, vomiting, etc . . .). Although some events such as the GI complaints, epistaxis, and rash could be drug-related and are known to occur with this product they could also be due to the subjects' underlying illnesses. Since the sponsor did not submitted the case forms for these studies, it is impossible for this medical reviewer to draw any conclusions regarding ibuprofen's safety profile in the pediatric populations that participated in these studies.

6. Overdose Data: (a.) AAPCC TESS ibuprofen data from the years 1994 through 1996 for children ≤ 2-years of age. (The 1997 report was not yet available.) (b.) Reports from the FDA's Spontaneous Reporting System. (c.)Reports from McNeil's CPC Drug Safety Reporting System.

The American Association of Poison Control Centers (AAPCC) Toxic Exposure Surveillance System (TESS) collected a total of 2,726,446 reports of possible human poisonings due to therapeutic drugs during the time period of 1994-1996. The sponsor has provided in this submission the data pertaining to ibuprofen overdoses. A total of

118,841 reports (4.4%) out of all of the reports collected for this time period were due to an ibuprofen containing product. In children < 2 years of age, there was a total of 17,635 reports of exposures to ibuprofen for this time period, out of which 17,173 (97.4%) were classified as non-toxic, minor, minimal or no effect reported. Of the remaining 462 case reports, 433 (2.3%) reported an unrelated effect or were lost to follow up. Although a total of 29 cases in this age group were classified as having resulted in a moderate (25 cases) or major (4 cases) outcome, none resulted in a death of a child. Only 24 out of these 29 cases with a moderate or major outcome involved either unknown pediatric formulations or an adult formulation of ibuprofen. Table 8-46, at the end of this section prepared by the sponsor lists these cases by increasing chronological age.

In addition, the sponsor obtained data from the FDA's Spontaneous Reporting System (SRS) for the time period November 1,1993 through August 25, 1997 and also queried its own data base for any case reports of ibuprofen overdoses in children < 2 years of age. This search of the SRS database yielded 22 reports, out of which 4 were listed as having serious outcomes. The following attached sponsor's table, Table 8-47, lists these 4 cases. Two of the 4 cases (MR 970170176 and MR 897009001S) which resulted in the death of a 23-month-old female child appear to be the same case. Review of the associated case reports reveals that this case was confounded by some underlying unspecified enzyme deficiencies as well as other congenital abnormalities in the child. The child reportedly suffocated on her vomitus while in bed after receiving an overdose of a competitor's ibuprofen suspension for the treatment of a fever. The other 2 cases involved a 17-month-old male who accidently ingested 27-28 tablets of an OTC adult formulation of ibuprofen. He was hospitalized for observation following emergency treatment for the drug overdose and survived without any reported sequelae. The last case was a report from worldwide literature about a 21-month-old male with a history of hypocalcemia and hypomagnesemia who was hospitalized for the treatment of a metabolic acidosis associated with drowsiness and tachypnea after an overdose of 8 grams of ibuprofen. He subsequently developed acute tonic-clonic seizures and renal failure, but reportedly recovered.

Medical Reviewer's Comments: Since little information is provided regarding whether the 24 cases of non-serious overdoses involved pediatric or unknown adult formulations of ibuprofen, this reviewer at best recommends that the indicated labeled age ranges for the pediatric formulations be modified to improve clarity. As such, it may be prudent to not have overlapping age ranges as one such attempt at minimizing dosing misadventures.

Table 8-46. Moderate or Major Outcomes with Single-Ingredient Ibuprofen Exposure in Children Less Than Two Years of Age from AAPCC TESS[1] Database, 1994 through 1996

Case	Date Rec'd	Age[2]	Sex	AAPCC Serial No	IBU Form[3]	Reason for Exposure	Clinical Effect	Outcome
1	12/27/94	25 d	M	090-34621	P	Adverse Drug Reaction	Erythema/flushed<br>Diaphoresis<br>Hypothermia	Moderate
2	3/23/96	5 m	F	036-2922990	A/U	Accidental: Therapeutic Error	Vomiting<br>Acidosis<br>Bleeding	Moderate
3	2/20/95	7 m	M	007-18561567	A/U	Accidental: Therapeutic Error	Vomiting<br>Electrolyte abnormality	Moderate
4	9/03/94	1 y	F	072-18204590	A/U	Accidental: General	Vomiting<br>Drowsiness/lethargy	Moderate
5	10/29/96	1 y	M	005-48762	P	Accidental: General	Vomiting<br>ADR to treatment<br>Other	Moderate
6	8/03/96	12 m	F	040-30080175	A/U	Accidental: General	Hypotension<br>Coma<br>Drowsiness/lethargy<br>Miosis	Major
7	10/23/95	13 m	M	083-94031093	A/U	Accidental: General	Other	Moderate
8	12/01/95	13 m	M	091-141435	P	Accidental: General	Vomiting<br>Drowsiness/lethargy<br>Other	Moderate
9	4/06/96	13 m	Unk	040-30055849	A/U	Accidental: General	Vomiting<br>Drowsiness/lethargy<br>Acidosis	Moderate
10	4/21/95	14 m	F	030-1174497	A/U	Accidental: General	Drowsiness/lethargy	Moderate

[1]American Association of Poison Control Centers Toxic Exposure Surveillance System
[2]Patient age in days (d), months (m), or years (y)
[3]Ibuprofen formulation - pediatric (P) or adult/unknown (A/U)

Table 8-46. Moderate or Major Outcomes with Single-Ingredient Ibuprofen Exposure in Children Less Than Two Years of Age from AAPCC TESS[1] Database, 1994 through 1996

Case	Date Rec'd	Age[2]	Sex	AAPCC Serial No	IBU Form[3]	Reason for Exposure	Clinical Effect	Outcome
11	10/31/95	14 m	F	047-77931	A/U	Accidental: General	Coma	Moderate
12	2/17/96	14 m	M	031-581263	A/U	Accidental: General	Dehydration<br>Agitation/irritable	Moderate
13	9/19/94	15 m	M	011-17732282	A/U	Accidental: General	Other	Moderate
14	10/24/94	15 m	M	006-17917397	A/U	Accidental: General	Drowsiness/lethargy<br>Acidosis	Moderate
15	1/04/96	15 m	M	022-591800	A/U	Accidental: General	Other LFT abnormality	Moderate
16	11/20/94	16 m	F	009-17413922	A/U	Accidental: General	Ataxia<br>Drowsiness/lethargy	Moderate
17	11/13/96	16 m	M	084-220194	A/U	Accidental: General	Drowsiness/lethargy<br>Acidosis<br>Hypoglycemia	Moderate
18	6/30/94	17 m	M	027-18761159	P	Adverse Drug Reaction	Hypothermia	Moderate
19	7/03/94	17 m	F	007-18396169	A/U	Accidental: General	Vomiting<br>Coma<br>Increased creatinine<br>Acidosis	Major
20	5/01/94	18 m	M	025-18230392	A/U	Accidental: General	Coma<br>Drowsiness/lethargy<br>Acidosis	Major
21	6/20/94	18 m	F	008-17642442	A/U	Accidental: General	Ataxia<br>Drowsiness/ lethargy<br>Acidosis	Moderate

[1]American Association of Poison Control Centers Toxic Exposure Surveillance System
[2]Patient age in days (d), months (m), or years (y)
[3]Ibuprofen formulation - pediatric (P) or adult/unknown (A/U)

Table 8-46. Moderate or Major Outcomes with Single-Ingredient Ibuprofen Exposure in Children Less Than Two Years of Age from AAPCC TESS[1] Database, 1994 through 1996

Case	Date Rec'd	Age[2]	Sex	AAPCC Serial No	IBU Form[3]	Reason for Exposure	Clinical Effect	Outcome
22	11/10/95	18 m	F	027-2687703	A/U	Accidental: General	Bradycardia<br>Hypertension<br>Drowsiness/lethargy<br>Acidosis<br>Hypothermia	Moderate
23	11/05/96	18 m	M	009-38152	A/U	Accidental: Therapeutic Error	Hypotension<br>Electrolyte abnormality<br>Hyperglycemia<br>Other	Moderate
24	3/31/94	19 m	M	010-17556117	A/U	Accidental: General	Dizziness/vertigo	Moderate
25	6/26/94	20 m	M	003-20104700	A/U	Unknown	Dysphagia<br>Dystonia	Moderate
26	9/28/95	21 m	M	011-2616142	A/U	Accidental: General	Diarrhea	Moderate
27	10/05/96	22 m	F	027-376272	A/U	Accidental: General	Acidosis	Moderate
28	5/02/96	22 m	F	031-575945	P	Adverse Drug Reaction	Dyspnea	Moderate
29	3/09/96	22 m	F	011-2647872	A/U	Accidental: General	Tachycardia<br>Drowsiness/lethargy<br>Acidosis	Major

[1]American Association of Poison Control Centers Toxic Exposure Surveillance System
[2]Patient age in days (d), months (m), or years (y)
[3]Ibuprofen formulation - pediatric (P) or adult/unknown (A/U)

Table 8-47. Ibuprofen Overdoses with Serious Outcomes in Children Less Than Two Years of Age: FDA Spontaneous Reporting System from November 1993 Through August 1997

Case	Date	Age[1]	Sex[2]	Mfr Control	Manuf	Drug	Dosage[3]	Duration	COSTART	Outcome	Concomitant Drugs
1	Jul 95	M17	M	94055	Whitehall	Advil	27-28 Tb	----	Accidental overdose	Hospitalized<br>Required<br>Intervention	---
2	May 97	M21	M	515517463	Upjohn	Ibuprofen	8 Gm	----	Grand mal convulsion<br>Hyperkalemia<br>Acute kidney failure<br>Accidental overdose	Hospitalized<br>Required<br>Intervention<br>Other	---
3	Jun 97	M23	F	970170176	Whitehall	Children's Advil	2 Tp	1 dose	Overdose<br>Aspiration pneumonia	Died	Cortef<br>Desmopressin Acet<br>Levoxyl
4	Feb 97	M23	F	897009001S	Whitehall	Advil	2 Tp	1 dose	Overdose<br>Vomiting	Died<br>Other	Cortef<br>Desmopressin Acet<br>Levoxyl

[1]Age in months (preceded by 'M')
[2]M = male, F = female
[3]Tb = tablets, Tp = teaspoon

APPEARS THIS WAY
ON ORIGINAL

Medical Reviewer's Overall Safety Comments: The sponsor has submitted an application in support of their request to lower the current approved age range from 2 to 3 years of age down to 2 months of age for their formulation of pediatric ibuprofen suspension. This product is currently available as a prescription drug for use in children 6 months to 2 years of age who are under a health care provider's care. Thus, the provider has made the determination as to the appropriateness of use of this product in this age group. This controlled access may account for the low incidence of reported post-marketing adverse events associated with ibuprofen suspension in children < 2 years of age. As noted above, most of the overdose safety data in the pediatric population was generated by inadvertent overdosing or accidental ingestion of adult ibuprofen products. At the September 18,1998 NDAC some of the committee members recommended that the age threshold for use of this product might be lowered down to 2 months based on the presentations of data at that meeting, but they also felt that additional warnings needed to appear on the label to safeguard against the use of the product in select populations where additional medical input was needed (i.e., preemies, children with significant fevers, fevers accompanied by lethargy, etc . . .) In face of the fact that the largest supporting source of safety data in a pediatric population < 6 months of age is heavily flawed, and the validity of some of its conclusions are questionable at best, this reviewer feels that there is insufficient safety data in the infant population < 6 months of age to support a lowering of the approved indicated age range to this level.

Recommendations: Based on the data contained in this submission Children's MOTRIN® (ibuprofen oral suspension) Drops, 50 mg/1.25 mL is safe to be used in an OTC pediatric population ≥ 6 months of age. There is insufficient data to currently support an age range lower than the above. Due to the possible threat of dosing misadventures due to consumer confusion, an overlap in dosing age ranges should be avoided for this product and its sister product, Children's MOTRIN® (ibuprofen) Suspension, 100 mg/5 mL. Thus, the concentrated drops should be labeled for use in children ≤ 2 years of age, and the less concentrated solution should be labeled for use in children ≥ 2 years of age. To further help prevent these incidents from happening in the future, the sponsor needs to re-label this product as "concentrate" as follows: Children's MOTRIN® (ibuprofen) Concentrated Drops, 50 mg/1.25 mL.

/S/

Rosemarie Neuner, MD. MPH
Medical Reviewer, HFD-560

/S/

Linda M. Katz, MD, MPH 3/29/99
Deputy Dir., HFD-560

CC: NDA 20-603 File
HFD-560 Div. File
HFD-550 Div. File

3/29/99 – The sponsor may want to consider modifying the name of the concentrated suspension (drops) to denote a product commensurate with the infant age group targeted for treatment. Thus, consideration could be given to modifying the product name to "Baby" "Pediatric" or "Infant" Motrin (ibuprofen oral suspension) drops. [illegible] "Baby" or "Infant" is preferred. [illegible]

HFD-560 ActingDir/Bowen Dut for Dr Bowen
HFD-560 Dep Dir/Katz
HFD-560 Team Leader/Lumpkins
HFD-550 Team Leader/Hyde
HFD-560 MO/Neuner
HFD-560 PM/KRothschild
